# Structural bases of signal generation and transduction by the SPS amino acid sensor of *Saccharomyces cerevisiae*

**DOI:** 10.1093/g3journal/jkaf312

**Published:** 2025-12-24

**Authors:** Peter Scharff-Poulsen, Morten C Kielland-Brandt

**Affiliations:** Department of Biotechnology and Biomedicine, Technical University of Denmark, 223 Søltofts Plads, DK-2800 Kongens Lyngby, Denmark; Department of Biotechnology and Biomedicine, Technical University of Denmark, 223 Søltofts Plads, DK-2800 Kongens Lyngby, Denmark

**Keywords:** amino acid receptor, amino acid sensor, LeuT fold, APC superfamily, APC family, nutrient sensing, signal transduction, Ssy1, Ptr3, Ssy5, Stp1, Rts1, WD40, protein kinases Yck1 and Yck2, protein phosphatase 2A, intrinsic disorder, LxxIxE motif

## Abstract

Ssy1 in *Saccharomyces cerevisiae* is an amino acid receptor evolved from amino acid transporters. It is situated in the plasma membrane in the SPS complex, together with the WD40-repeat protein Ptr3 and the endoprotease Ssy5. Binding of extracellular amino acids to Ssy1 triggers liberation of the catalytic domain of Ssy5, which removes an inhibitory domain from the transcription factor Stp1, freeing it to activate genes encoding amino acid transporters. We mapped 7 constitutively signaling and hyper-responsive *SSY1* mutations onto AlphaFold and Phyre2-based 3D models of Ssy1 to inform conformational steps involved in signaling. The predictions suggest a model in which an occluded, inward-facing conformation of Ssy1 leads to signaling. The mutations suggest a hinge in TM12 which, combined with a C-terminal “latch,” offers a mechanism for signaling. AlphaFold 3 modeling suggests that conserved sequence boxes in the N-terminal cytoplasmic domain of Ssy1 serve as interaction faces for binding of Ptr3, Ssy5, and casein kinases Yck1 and Yck2 (Yck). In addition, interaction faces between Ptr3 and Ssy5 were predicted. Antagonism between phosphorylation and dephosphorylation of Ptr3 and Ssy5 by Yck and Protein Phosphatase 2A (PP2A) is key in signaling. We found Yck phosphorylation motifs as well as binding motifs for regulatory subunit Rts1 of PP2A in both Ptr3 and Ssy5. These motifs, together with sites of *PTR3* and *SSY5* gain-of-function mutations, were mapped onto AlphaFold models of Ptr3 and Ssy5. The results constitute a basis for predicting novel aspects of phosphorylation in the signaling mechanism.

## Graphical Abstract

**Graphical Abstract:**
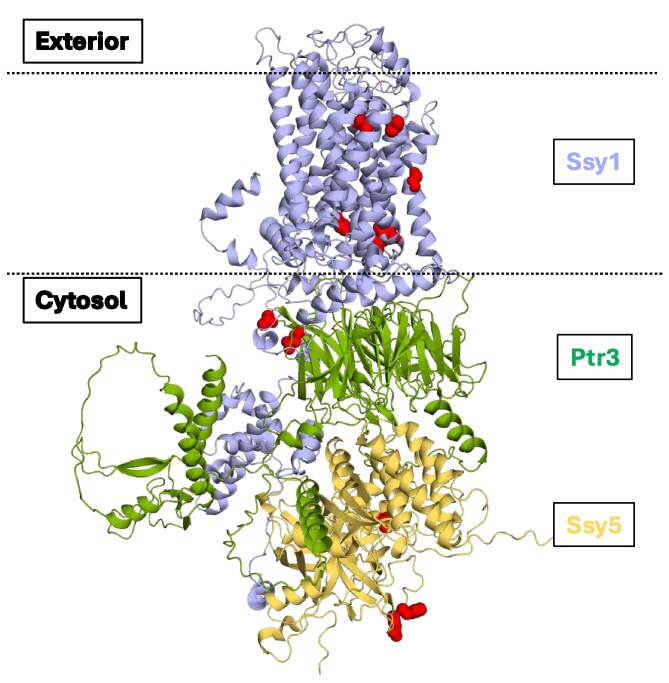

## Introduction

When *Saccharomyces cerevisiae* is exposed to amino acids, the SPS-sensor complex (Forsberg and Ljungdahl 2001), consisting of Ssy1, Ptr3, and Ssy5, generates a signal that leads to production of transporters of amino acids and dipeptides. Several laboratories have contributed to our current view of the mechanism of this sensor, beginning with the discovery of the sensing of micromolar concentrations of leucine (Island et al. 1987; Didion et al. 1996) and identification of the amino acid receptor Ssy1 (Didion et al. 1998; Jørgensen et al. 1998; Iraqui et al. 1999; Klasson et al. 1999). Signaling requires the WD40-repeat protein Ptr3 (Island et al. 1991; Barnes et al. 1998), known to be phosphorylated upon signaling by redundant casein kinases Yck1 and Yck2 (hereinafter designated Yck; Liu et al. 2008), which are anchored to the plasma membrane (Vancura et al. 1994; Roth et al. 2002; Babu et al. 2004; Pasula et al. 2010). Protein phosphatase 2A with the regulatory subunit Rts1 (PP2A^Rts1^) negatively regulates SPS sensing (Eckert-Boulet et al. 2006; Omnus and Ljungdahl 2013), initially by dephosphorylation of Ptr3, thereby opposing Yck activity (Liu et al. 2008). Ptr3 plays an essential role as an adaptor that transduces ligand-induced conformational changes of Ssy1, resulting in phosphorylation of Ssy5 (Omnus and Ljungdahl 2013), a chymotrypsin-like serine endoprotease indispensable for signaling. Already before signaling, Ssy5 is self-cleaved into an N-terminal prodomain and a catalytic domain, which persist together as an inactive protease component of the SPS sensor (Andréasson et al. 2006; Poulsen et al. 2006; Martins et al. 2018). In response to the binding of amino acids to Ssy1, Yck adds phosphate groups to the Ssy5 prodomain, leading to recruitment of the Skp1/Cullin/Grr1 (SCF^Grr1^) E3 ubiquitin ligase and subsequent ubiquitylation and proteasomal degradation of the prodomain (Pfirrmann et al. 2010; Abdel-Sater et al. 2011; Omnus et al. 2011; Omnus and Ljungdahl 2013). This cascade of events liberates the Ssy5 catalytic domain, which then activates the transcription factors Stp1 and Stp2 (Jørgensen et al. 1997; de Boer et al. 2000) by a specific cleavage (Andréasson and Ljungdahl 2002, 2004; Abdel-Sater et al. 2004; Wielemans et al. 2010; Tumusiime et al. 2011; Omnus and Ljungdahl 2014). The transporters that are induced are mainly those that import the amino acids that are most potent in initiating the signal (Iraqui et al. 1999; Regenberg et al. 1999; Gaber et al. 2003). While SPS sensing primarily controls amino acid and peptide import, it also directly regulates genes involved in a few other functions (Dubois and Wiame 1976; Klasson et al. 1999; Zekhnini et al. 2023).

The present report deals with early steps of signaling and exploits recent opportunities for modeling Ssy1 conformations based on structural homology. Experimental determination of a 3D structure of Ssy1 has not been achieved, so we developed 3D models of Ssy1 based on recently reported structures of amino acid transporters. We applied the models for a mechanistic interpretation of a collection of gain-of-function *SSY1* mutants to elucidate details of early steps in amino acid sensing.

In addition, we predict protein phosphatase 2A (PP2A^Rts1^) binding sites and Yck phosphorylation sites in Ptr3 and Ssy5. Combined with the positions of gain-of-function mutations in AlphaFold models of Ptr3 and Ssy5, our analysis suggests how phosphorylation and dephosphorylation of the 2 proteins are antagonistic in signaling. By coordinating these findings with AlphaFold 3 interaction studies of the SPS-sensor components, we provide a structure-based model of the SPS complex that suggests new details of the signal transduction process.

### Ssy1 is a transporter-like receptor

The amino acid receptor Ssy1 belongs to the family of yeast amino acid transporters (YATs), having robust sequence similarity with transporters in this family (Jørgensen et al. 1998; Gournas et al. 2016). This suggests a transporter as the evolutionary origin of Ssy1 and led to the ideas that amino acids interact with a centrally located binding site of Ssy1 and that Ssy1 can assume inward-facing and outward-facing conformations, like transporters (Wu et al. 2006). Because transport of amino acids by the receptor is undetectable (Didion et al. 1998; Iraqui et al. 1999), Wu et al. (2006) proposed that the presence of an amino acid molecule in the binding site inhibits shifts between outward-open and inward-open conformations of Ssy1. According to this proposal, Ssy1 can assume an outward-open conformation, allowing extracellular amino acids to enter reversibly into the binding site. Similarly, an inward-open conformation should allow amino acids to enter reversibly from the cytosol into the binding site, and indeed cytoplasmic leucine has an inhibitory effect on sensing of extracellular amino acids (Wu et al. 2006). The idea is that binding of extracellular amino acids stabilizes a signaling conformation, whereas binding of cytoplasmic amino acids stabilizes a nonsignaling conformation.

### The signaling conformation of Ssy1

On the basis of the results and considerations cited above, we (Wu et al. 2006) originally proposed a model for signaling by Ssy1 that was minimalistic in the sense that it required only 2 conformations, an outward-open and an inward-open conformation. This led to a model in which the signaling conformation was outward open. However, it has since become increasingly clear that transporters in general go through occluded conformations. Thus, as reviewed (Licht et al. 2024), 45 3D structures of amino acid transporters in the amino acid-polyamine-organo cation (APC) family are now available for modeling in several conformations, including occluded ones. This has contributed to our present revision of the model of Wu et al. (2006). Already van Zeebroeck et al. (2009) proposed the signaling conformation of Ssy1 to be occluded by analogy with their own study of sensing through the bifunctional transporter and receptor (transceptor) Gap1. In terms of evolution, it would also make a priori sense that a conformational change initiated by substrate-induced fit in an APC transporter (Kowalczyk et al. 2011) is reused in a receptor. We will below argue that the available data are consistent with an occluded, inward-facing conformation of Ssy1 being signaled. An additional motivation for the present work has been an analogous analysis of constitutively signaling mutant forms of the yeast transporter-like glucose sensor Rgt2 (Scharff-Poulsen et al. 2018), which suggested that the signaling conformation of Rgt2 is inward facing.

## Methods

Protein sequence alignments were carried out using Clustal Omega, applying the clustalW character counts output format (https://www.ebi.ac.uk/jdispatcher/msa/clustalo; Madeira et al. 2024). Subsequently, the aligned sequences were visualized with graphical enhancements using ESPript (https://espript.ibcp.fr/ESPript/ESPript/; Robert et al. 2025).

Modeling of Ssy1 structures was performed at the PHYRE2 Protein Fold Recognition Server (http://www.sbg.bio.ic.ac.uk/phyre2/html/page.cgi?id=index; Kelley et al. 2015) using “Normal” and “Intensive” modeling modes. The Fold and Function Assignment Server (FFAS) (https://ffas.godziklab.org/; Xu et al. 2014) was employed in some searches for Ssy1 model structures, using the Protein Data Bank (PDB) and the protein families (Pfam) databases.

Protein structures were retrieved from the PDB (https://www.rcsb.org/; Burley et al. 2025) and the AlphaFold Protein Structure Database (https://alphafold.ebi.ac.uk/; Fleming et al. 2025).

PyMOL (https://pymol.org/2/) was used for visualization, analysis, and preparation of figures of protein structures.

The Metapredict (v2.4) web server (https://metapredict.net/; Emenecker et al. 2021) and the Critical Assessment of protein Intrinsic Disorder prediction portal (https://caid.idpcentral.org/submit) were applied for prediction of intrinsically disordered regions (IDRs).

To predict positions and orientations of proteins in the SPS complex, we used the AlphaFold3 Server (https://alphafoldserver.com; Abramson et al. 2024).

Initial searches for known interactions with kinases and phosphatases were carried out with the *S. cerevisiae* kinase and phosphatase interactome resource (https://yeastkinome.org/index.php).

Amino acid sequences of *S. cerevisiae* proteins and other genomic information on *S. cerevisiae* were downloaded from the Saccharomyces Genome database (http://www.yeastgenome.org; Engel et al. 2025).

## Results and discussion

### Topology features of Ssy1

Ssy1 has the transporter classification number (TC#) 2.A.3.10.12 and belongs to the YAT family (TC# 2.A.3.10), which is a subset of the APC family (TC# 2.A.3), belonging to the larger APC superfamily (http://www.tcdb.org; Edwards et al. 2018; Saier et al. 2021). The APC superfamily includes Na^+^- and H^+^-coupled amino acid symporters and amino acid antiporters across all kingdoms of life. They transport a wide spectrum of amino acids and amino acid derivatives. The members vary greatly in their substrate preference, with some being specific for a single or a few amino acids and others having broad amino acid preferences. Such different patterns of preference are also observed within the YAT family of *S. cerevisiae* amino acid transporters (Regenberg et al. 1999; Gournas et al. 2016, 2018).

While bona fide *S. cerevisiae* amino acid transporters have lengths close to 600 amino acid residues, Ssy1 has a length of 854 residues (aligned in Supplementary Fig. 1). This is mainly due to a long cytoplasmic N-terminal domain of about 280 residues and 2 remarkable extracellular loops (ELs), EL5-6 and EL7-8, with lengths of ∼60 and 70 residues, which are about 20 and 30 amino acids longer, respectively, than the corresponding loops of transporters (Supplementary Fig. 1).

### Evolutionary relationships of Ssy1

To gain insight into functional and evolutionary aspects of *S. cerevisiae* Ssy1, we compared its amino acid sequence with sequences of orthologs. We aligned Ssy1 with 36 fungal homologs (Supplementary Fig. 2) retrieved from the UniProt portal (https://www.uniprot.org) using BLAST. The alignment reveals low sequence similarity between the N-terminal domains (residues 1 to 282). In contrast, the 12-helix TransMembrane bundle (TM bundle; residues 283 to 801) shows high similarity, with 35 residues fully conserved and many regions highly conserved. We used this alignment (Supplementary Fig. 2) to interpret gain-of-function mutants and reveal conserved interactions between helices and loops in Ssy1 orthologs. Since most of the Ssy1 orthologs have only been identified in genome sequencing projects, we cannot know which are amino acid receptors, but it seems reasonable to consider them functional homologs.

In an alignment of Ssy1 with the 15 members of the YAT family of amino acid transporters from *S. cerevisiae*, only 25 positions are fully conserved (Supplementary Fig. 1). A comparison of Ssy1 with each individual transporter shows sequence identities in the range from 20% to 27% and similarities from 32% to 42% (Supplementary Table 1). Supplementary Figure 3 and its legend present a Phyre2 structure model of Ssy1 highlighting the 25 conserved residues and literature describing the functional significance of some of them in transporters.

*Candida albicans*, an opportunistic pathogen to humans, has an Ssy1 homolog that is likewise responsible for sensing of extracellular amino acids, signaling through a pathway involving homologs of *S. cerevisiae* Ptr3, Ssy5, and Stp1/2. Remarkably, the preferences/specificities for amino acids are very different from those of *S. cerevisiae* (Martínez and Ljungdahl 2005; Silao et al. 2019). More complex peptidoglycan fragments are also sensed by *C. albicans* in an Ssy1-dependent manner, yet through a different signal pathway (Li et al. 2025). Any possible sensing of peptidoglycan fragments in *S. cerevisiae* through Ssy1 has, to our knowledge, not been studied.

### Structure models of Ssy1

We previously (Poulsen et al. 2008) proposed a 3D model of Ssy1 based on the structure of the distantly related bacterial Na^+^/leucine symporter LeuT (Yamashita et al. 2005). To improve the model and to generate models of several conformations, we searched the Phyre2 server (Kelley et al. 2015) for new suitable templates. In addition to amino acid transporters, the analysis yielded several Na^+^-K^+^-Cl^−^ transporters, also in the APC superfamily, but we decided to base Ssy1 models only on transporters of amino acids and similar compounds, since they are functionally related to Ssy1 through similar ligands and binding pockets (Supplementary Table 2). These templates comprise amino acid symporters and antiporters, a serotonin transporter, and a dopamine transporter, all of which have the canonical LeuT fold (Yamashita et al. 2005) and thus belong to the APC superfamily. A search on the FFAS (Xu et al. 2014) yielded essentially the same list of potential templates for Ssy1 (not shown).

**Fig. 1.**
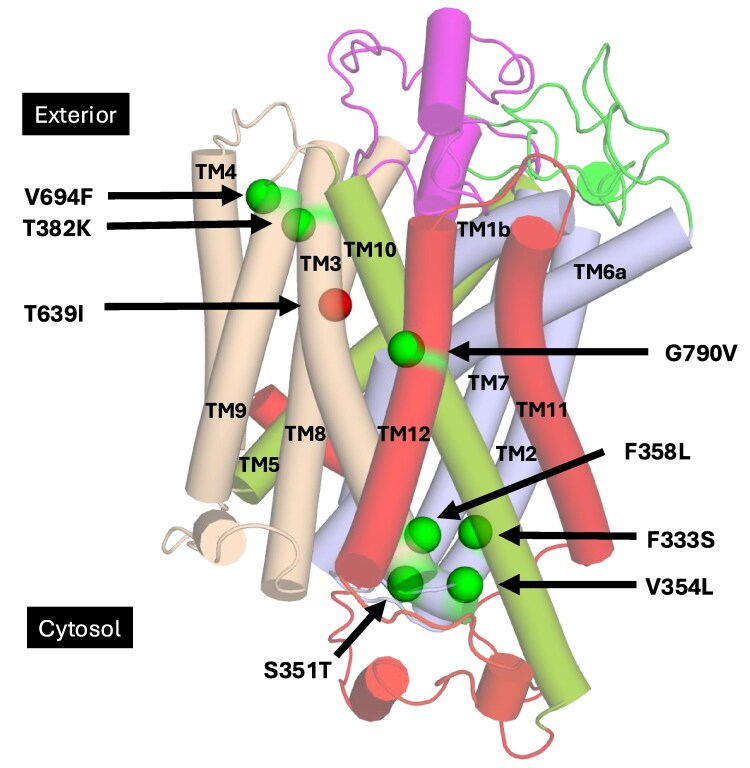
Positions of gain-of-function mutations in Ssy1. AlphaFold model of Ssy1 (UniProt accession number Q03770) shown with cylindrical, transparent helices. N-terminal domain residues 1 to 282 hidden for clarity. Bundle TMs 1, 2, 6, and 7 (light blue). Scaffold subdivided in Hash TMs 3, 4, 8, and 9 (wheat), Arms TM5 and TM10 (split pea). TM11, TM12, and C-terminal (red). Constitutive mutants (green spheres). Hypo-responsive mutant T639I (red sphere). EL5-6 (green) and EL7-8 (magenta).

**Fig. 2.**
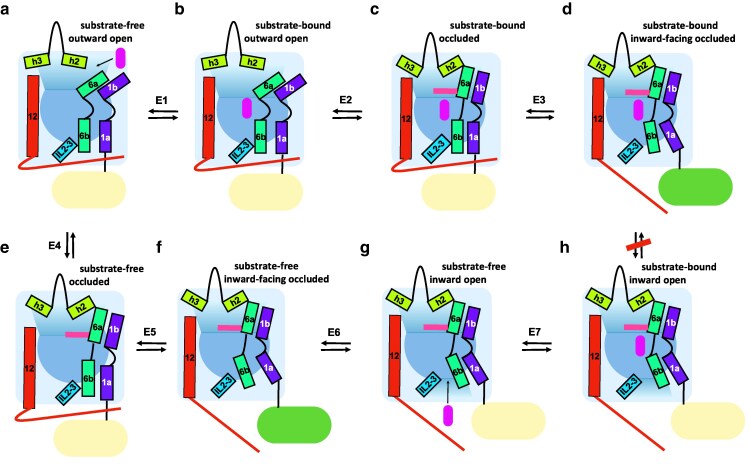
Equilibrium of proposed states of amino acid receptor Ssy1. Schematic side view of gates and most mobile transmembrane helices according to Phyre2 modeling of Ssy1 based on closest related transporters of the APC superfamily. Templates were as follows: substrate-free outward open (a) *Escherichia coli* AdiC (7O82); substrate-bound outward open (b) *E. coli* AdiC (3OB6); substrate-bound occluded (c) *Homo sapiens* LAT1 (7DSQ and 7DSL), *Aquifex aeolicus* LeuT (2A65) and *E. coli* AdiC (3L1L); substrate-bound inward-facing occluded (d) *Geobacillus kaustophilus* ApcT (5OQT and 6F34); substrate-free occluded (e) no template; substrate-free inward-facing occluded (f) *Methanocaldococcus jannaschii* ApcT (3GIA); substrate-free inward open (g) *H. sapiens* LAT1 (6JMQ) and *Carnobacterium* sp. BasC (6F2G); substrate-bound inward open (h) *H. sapiens* LAT1 (6IRT) and *Carnobacterium* sp. BasC (6F2W). Whereas direct interconversion between states d and h is needed in transporters, we propose it to be absent or of low efficacy in Ssy1. States d and f, with inward-facing occluded conformations, are proposed as the signaling states. The N-terminal domain is indicated with light yellow color, shifting to green in the signaling states, in which it has a swung-out position important for interactions with Yck1, Ptr3, and Ssy5. Magenta, filled, rounded bar indicates the ligand amino acid. Pink bar indicates the proximal thin gate, F512. Red thin line indicates the C-terminal domain. Reactions are proposed to be reversible and are numbered E1 through E7. Literature references for template structures and genus names of organisms are given in the text or Supplementary Table 2.

The LeuT fold is a common architecture in a variety of transporters despite their remarkably low sequence similarities (Forrest and Rudnick 2009; Boudker and Verdon 2010; Schweikhard and Ziegler 2012; Shi 2013; Ponzoni et al. 2018). Briefly, the LeuT fold comprises transmembrane helices TM1–TM5, which are structurally related to TM6–TM10 by a pseudo 2-fold symmetry axis located in the plane of the membrane. TM1 forms 2 alpha helices named TM1a and TM1b, connected by a short loop that contributes to the substrate-binding site. In symmetry with TM1, TM6 consists of helices TM6a and TM6b, connected by a short loop that also contributes to the substrate-binding site (Figs. 1 and 2). TMs 1–2 and TMs 6–7 together form a 4-helix bundle domain; 6 helices (TMs 3–5 and 8–10) form a scaffold domain folding around the bundle domain; the scaffold is subdivided into so-called hash (TMs 3, 4, 8, and 9) and arms (TMs 5 and 10; Yamashita et al. 2005; Forrest and Rudnick 2009; Krishnamurthy et al. 2009 ; Zhang et al. 2018). Two additional helices, TM11 and TM12, reside in the periphery of the transporter structure, where they form a V-shape at the external side of TM10. In the rocking-bundle model for transport, alternating access to a central substrate-binding site is allowed from either the extra- or intracellular side (Forrest and Rudnick 2009). This is achieved by a core bundle-hash rock motion conserved in all LeuT-fold superfamily members, yet with additional helix motions in individual transporters (Licht et al. 2024).

In the Phyre2-based Ssy1 models, structural information on parts of the N-terminus, EL5-6, EL7-8, and the C-terminus is not included. We therefore used a structure prediction of Ssy1 from *S. cerevisiae* included in the AlphaFold Protein Structure Database (UniProt accession number Q03770; Jumper et al. 2021) to predict the structure of these domains. In general, the AlphaFold model agrees well with the Phyre2 models but by itself obviously gives limited information on the outward-to-inward transitions of Ssy1.

### LeuT-fold transporters and their transport mechanisms suggest steps in the Ssy1 signaling mechanism

More than 300 LeuT-fold transporter structures now available in the PDB describe a rocking-bundle transport cycle including 8 general steps of the outward-to-inward transitions (Licht et al. 2024). Correspondingly, Phyre2-based Ssy1 models (Supplementary Table 2) can be applied for describing conformational changes of Ssy1 associated with signaling. We thus propose that Ssy1 can pass through/into all 8 states shown in Fig. 2 according to the 7 indicated reversible reactions. The 8 states represent 4 conformations with or without ligand amino acid. We use the terms “outward open,” “occluded,” “inward-facing occluded,” and “inward open” according to their use by the authors of the determinations of the template structures. In addition to its sensing function, Ssy1 must act differently from the transporters in a way that results in (i) its inability to transport at a detectable level (Didion et al. 1998; Iraqui et al. 1999) and (ii) the inhibition of signaling by intracellular leucine (Wu et al. 2006). A simple hypothesis that combines these 2 features is that the presence of an amino acid molecule in the ligand-binding site inhibits a step in the transitions of Ssy1 between an outward-open and an inward-open conformation. That can explain the lack of efficient transport, and it opens the possibility for leucine to act differently if it enters Ssy1 from the medium or from the cytosol. Figure 2 is based on the alternating-access rocking-bundle transport mechanism and depicts control of the extracellular gate by movements of TM1b, TM6a, and a plug structure, as well as control of the intracellular gate by movements of TM1a, TM6b, and a latch.

In the LeuT fold, access to or from the ligand binding site can be controlled by so-called “thin gates,” consisting of one or a few residues, and by more complex “thick gates” made up by several packed residues (Krishnamurthy et al. 2009; Beckstein and Naughton 2022). In Ssy1, the long EL7-8 may participate in a thick gate at the extracellular side. Similarly, the long cytoplasmic N-terminal and C-terminal domains may contribute to a thick intracellular gate.

There are 2 important differences between Fig. 2 and rocking-bundle transport. Firstly, the direct interconversion of the 2 ligand-bound states D (inward-facing occluded) and H (inward open), which is needed in a transporter, is proposed to be absent or of low efficacy in Ssy1. Secondly, the ligand-bound state D is proposed to be signaling. Since the ligand-free state F has a conformation similar to state D, we further hypothesize that state F is also signaling. This is in accordance with the observation that wild-type cells grown in the absence of extracellular amino acids have a low (basal) level of signaling, while a high basal level of signaling is observed in *SSY1* gain-of-function mutants (Poulsen et al. 2008). The hypothesis that a modest abundance of a ligand-free signaling state (F) of wild-type Ssy1 is responsible for most of the basal level of signaling also accords with the existence of an *ssy1* mutant (T639I) that can signal (Bernard and André 2001) but has an exceedingly low basal level of signaling (Poulsen et al. 2008). Our proposal that one of the occluded conformations of Ssy1 is signaling corresponds well with the proposal by Van Zeebroeck et al. (2009) that the signaling conformation of the *S. cerevisiae* Gap1 transceptor is occluded and the proposal by Scharff-Poulsen et al. (2018) that the signaling conformation of the glucose receptor Rgt2 is inward facing. As already pointed out, our proposal is different from our original model for signaling and nonsignaling conformations of Ssy1 (Wu et al. 2006).

The model (Fig. 2) explains how extracellular amino acids cause signaling by shifting the equilibrium between states A and B (E1) toward the right, thereby increasing the amount of Ssy1 in the signaling state D. Conversely, intracellular leucine inhibits signaling by shifting the equilibrium between states G and H (E7) to favor state H, thereby reducing the amounts of Ssy1 in the signaling states D and F.

The structural basis for the proposed lack of direct interconversion between states D and H of Ssy1 is unknown. However, blocks that hinder a complete transport cycle are common. Thus, as illustrated (Kowalczyk et al. 2011; Del Alamo et al. 2022), direct interconversion between states A and E (Fig. 2, reaction E4) of the antiporters AdiC and GadC is not possible. Instead, after extracellular arginine is bound to state A, the antiporter goes through states B, C, and D to state H, from which arginine is released to the cytosol. Cytosolic agmatine is then bound to the antiporter in state G, which returns to the outward-facing state A through states H, D, C, and B, allowing extracellular release of agmatine. This block of conformational change of a substrate-free transporter is a general and necessary feature of antiporters.

### Interpretation of hyper-responsive and constitutive *SSY1* mutants

The Phyre2-generated models of Ssy1 (Supplementary Table 2) provide structural frameworks for improved 3D localization of the residues altered in the 7 *SSY1* gain-of-function mutants. These mutants constitutively signal and are, in addition, hyper-responsive, i.e. low levels of amino acids give full signaling. An additional mutant requires very high amounts of extracellular amino acids to give signaling (hypo-responsive) and has a lower basal level of signaling than the wild type. The 3D models suggest the consequences of the mutations for interactions with spatially neighboring amino acid side chains.

We chose the AlphaFold model of Ssy1 (Q03770), which is in its outward-open conformation (Supplementary Fig. 5), for a spatial mapping of the constitutive and hyper-responsive *SSY1* mutants generated by Poulsen et al. (2008). Figure 1 shows locations of the mutated residues in this model. Most of them are located either at the extracellular face or the cytoplasmic face of Ssy1; only one is centrally located (in TM12). None of the mutations are placed in the ligand binding site, the long ELs, or the N- and C-terminal domains.

The influence of each gain-of-function mutation on the energy landscape of the 8 states of Ssy1 and the paths between them must be subtle enough that basic sensor function is not destroyed. It is also notable that none of these 7 mutations affect the 25 residues fully conserved in the YAT family (Supplementary Figs. 1 and 2), consistent with the hypothesis that most mutations changing the conserved residues would compromise basic functionality of YAT members, including Ssy1. This observation supports the idea that conformations and dynamics of Ssy1 have strong similarities to those of the transporters.

#### Mutations at the extracellular face of Ssy1

##### Replacement of T382 with bulky or charged residues promotes closure of the extracellular gate

The level of Stp1 processing is the most direct quantitative measure of SPS signaling available (Andréasson and Ljungdahl 2002; Poulsen et al. 2005a). The elevated basal Stp1 processing imposed by the T382K mutation is about half the fully induced level and is the highest basal level conferred by a single *SSY1* mutation (Poulsen et al. 2008), indicating that position 382 is particularly susceptible to mutations that push Ssy1 toward a signaling conformation. The T382K mutation presumably changes the equilibrium constants of reactions E2 and E4 (Fig. 2) to favor states C and E, respectively. In the presence of extracellular ligand, the resulting increased amount of signaling state D explains the hyper-responsive phenotype. In the absence of extracellular ligand, the increased amount of signaling state F explains the constitutive phenotype.

Residue T382 is placed in the extracellular end of TM3 and has been characterized by substitution with 16 different amino acid residues (Gaber et al. 2003). Mutations T382K, T382R, T382H, and T382L confer increased levels of amino acid-independent signaling, showing that replacement of the threonine residue with bulky and positively charged residues promotes a signaling Ssy1 conformation. T382 is only conserved in 35% of the aligned Ssy1 orthologs, but none of the Ssy1 orthologs contain lysine, arginine, histidine, or leucine residues in this position (Supplementary Fig. 2).

**Fig. 3.**
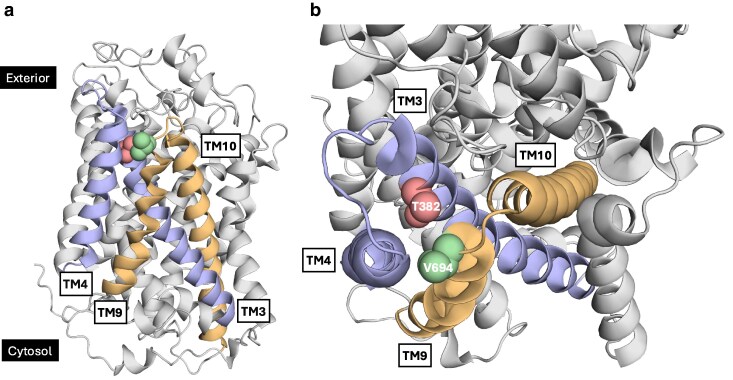
*SSY1* gain-of-function mutations T382K and V694F promote a signaling conformation by inserting larger side chains between TM3 and TM4. a) Side view of an Ssy1 AlphaFold model (Q03770), which has an outward-open conformation. The side chains of residues T382 in TM3 and V694 in TM9 are highlighted as spheres colored salmon and pale green, respectively. TMs 3 and 4 are colored light blue, TMs 9 and 10 are colored light orange, and the remaining helices are shown in gray. b) Extracellular view showing a close-up of the region containing residues T382 and V694.

In the AlphaFold model of Ssy1 (Q03770), the side chain of T382 is pointing toward TM4 and TM9 (Fig. 3). Presumably, replacement of T382 with the bulky or charged residues leads to clashing with neighboring residues in TM4 and TM9, resulting in local structural changes that promote the major conformational changes associated with closure of the extracellular gate.

##### Mutation V694F may promote a conformational shift of TM10 and EL9-10 leading to closure of the extracellular gate

In the gain-of-function mutation V694F, valine is substituted with the more bulky phenylalanine. While valine is highly conserved in Ssy1 orthologs, phenylalanine is absent in this position (Supplementary Fig. 2). In the Ssy1 AlphaFold model (Fig. 3), V694 is located in the extracellular end of TM9 with its side chain pointing toward TM4 and close to T382 in TM3. To understand the effect of this mutation, we made structural alignments of Ssy1 models based on AdiC in outward open and occluded conformations (Supplementary Fig. 4). They exhibit a considerable displacement of TM10 and of V694 during the transition from the outward open to the occluded state; V694 moves away from a position between TM3 and TM4 to a position where the side chain points toward the rim of the extracellular face of Ssy1. We suggest that a similar displacement is facilitated by the V694F mutation, because the introduction of the bulky phenylalanine side chain results in steric clashes with neighboring residues in TM3 and TM4, thus promoting inward-facing signaling conformations. This is in accordance with the observation that hash helices TM3 and TM4 in the APC superfamily form a rigid body and that movement of the arm helix TM10 participates in outward closing (Licht et al. 2024).

##### Mutations T382K and V694F may promote closure of the extracellular gate by inserting a “wedge” between TM3 and TM4

Since the bulky side chains introduced by T382K and V694F are expected to protrude into the same space between TM3 and TM4 (Fig. 3b), we suggest that both mutations affect Ssy1 function by inserting a “wedge” in this part of Ssy1 and thereby promote signaling conformations.

In the absence of ligands, mutations T382K and V694F may promote closure of the extracellular gate by changing the equilibrium constant of reaction E4 to favor states E and F (Fig. 2). This would result in an increased basal level of signaling, as we observed previously (Poulsen et al. 2008). Likewise, in the presence of ligands, mutations T382K and V694F may change the equilibrium constant E2 to favor ligand-bound states C and D with a closed extracellular gate (Fig. 2). This would cause a decrease in *EC*_50_ as we previously found (Poulsen et al. 2008).

##### The T382K and V694F mutations may promote closure of the extracellular gate by a “plug” formed by EL7-8

The AlphaFold model of Ssy1 (Q03770) predicts a 2-helix motif in EL7-8 (Supplementary Fig. 5), which is similar to 2-helix motifs found in the 3D structures of several amino acid transporters with a LeuT fold (Yamashita et al. 2005). Upon binding of substrate, the helical segments move inward to plug the extracellular vestibule (Krishnamurthy and Gouaux 2012). Analogous 2-helix motifs acting as lids that seal the extracellular gate are also found in the structures of LAT1 (6JMQ; Lee et al. 2019), LAT1 (6IRT; Yan et al. 2019), and LAT2 (7CMH; Yan et al. 2020) in their inward-open conformations. A similar closing mechanism for the extracellular gate has been described for GadC, captured in an outward-closed conformation (4DJI; Ma et al. 2012). In this case, EL7-8 interacts with EL3-4, EL9-10, and EL11-12 through an extensive network of H bonds and hydrophobic interactions that form a plug closing the extracellular gate.

EL7-8 in Ssy1 may well have a closing function similar to those described for LeuT, LATs, and GadC, but if so, its size of ∼70 residues opens the possibility of additional, unknown function(s). The AlphaFold interpretation of the outward-open Ssy1 sets the stage for a possible complex cap/lid structure involving the ELs (Supplementary Fig. 5) in which EL7-8 forms a plug that could dig into the extracellular gate. We suggest that mutations T382K and V694F facilitate sealing of the extracellular gate by the EL7-8 plug structure.

#### Interpretation of mutations located at the intracellular face of Ssy1

##### Mutation F333S likely disrupts an F333–F521 pi–pi interaction and facilitates a displacement of TM6b in the intracellular gate

The F333S mutation results in a rather high level of Stp1 processing in the absence of extracellular amino acids (Poulsen et al. 2008). In a model of Ssy1 based on AdiC (7O82; Ilgü et al. 2021) in its outward-open conformation (Supplementary Fig. 6a), the side chains of residues F333 in TM2 and F521 in TM6b are packed above each other, which suggest stabilization by pi–pi stacking between the 2 aromatic rings. Whereas F333 is conserved in the Ssy1 orthologs (Supplementary Fig. 2), F521 is conserved in only about half of them. Nevertheless, 14 orthologs have a cysteine residue in the position corresponding to F521. A phenylalanine–cysteine interaction is generally stronger than a phenylalanine–phenylalanine interaction (Gómez-Tamayo et al. 2016). We therefore suggest that both types of interaction attach TM2 to TM6b and thereby favor a closed intracellular gate.

Remarkably, the F333–F521 interaction connects the two 5-helix inverted repeats characteristic of the LeuT fold, suggesting a feature for closure of the intracellular gate that is specific for Ssy1 and its orthologs. Thus, bona fide YAT transporters do not contain corresponding residues for phenylalanine–phenylalanine and cysteine–phenylalanine interactions (Supplementary Fig. 1). In a broader perspective, F333 and F521 may contribute to a hydrophobic cluster with F346, Y366, L522, I716, W717, and L720, which form the bottom of the binding pocket and participate in closure of the intracellular gate (Supplementary Fig. 7a). These features could in some way contribute to the proposed inability of states D and H (Fig. 2) to directly interconvert.

In transporters, TM6b is fully displaced to allow substrate release into the cytosol during the transport cycle. However, for Ssy1 in its ligand-bound inward-facing occluded conformation, we propose that full displacement of TM6b is obstructed, i.e. direct interconversion of states D and H is blocked (Fig. 2). In contrast, we propose that full displacement of TM6b is possible for the ligand-free inward-facing occluded conformation of Ssy1, ie interconversion of states F and G is allowed. This full displacement of TM6b is illustrated by an Ssy1 model based on LAT1 (6IRT) in an inward-open conformation (Supplementary Fig. 6b), in which F521 is unavailable for pi–pi interaction with F333.

Since the pi–pi stacking is eliminated by the F333S mutation, reaction E3 may shift to favor the signaling state D in the presence of ligands (Fig. 2). This rationalizes the hyper-responsive phenotype of the mutant characterized by more than 4-fold drops of *EC*_50_ toward any of the 3 tested amino acids (leucine, phenylalanine, and norvaline; Poulsen et al. 2008). Similarly, the F333S mutation may shift reaction E5 to favor the signaling state F in the absence of ligands, rationalizing the constitutitve phenotype of the mutant (Poulsen et al. 2008).

##### A mutational hotspot in the intracellular loop IL2-3

The residues changed by the gain-of-function mutations S351T and V354L are located in the intracellular loop (IL2-3) between helices TM2 and TM3, while F358L is in the cytosolic end of TM3 (Fig. 1). S351, V354, and F358 are fully or almost fully conserved in Ssy1 orthologs (Supplementary Fig. 2), while none of these 3 residues are conserved in the 15 bona fide YAT transporters from *S. cerevisiae* (Supplementary Fig. 1). S351, V354, and F358 neighbor residues S347, G348, R352, G359, F360, and Y366, which are fully conserved in the fungal Ssy1 orthologs (Supplementary Fig. 2); this block of residues is either not conserved or only partially conserved in the alignment of YAT transporters (Supplementary Fig. 1).

IL2-3 is an evolutionarily conserved structural element in the LeuT fold and corresponds to EL7-8 by the pseudo 2-fold symmetry axis. As observed in the structure of MjApcT, loops IL2-3 and EL7-8 provide the “bottom” and “top,”, respectively, of the substrate-binding site (Shaffer et al. 2009). However, whereas EL7-8 undergoes large relative movements as discussed above, the position of IL2-3 is rather constant in LeuT (Krishnamurthy and Gouaux 2012). Nevertheless, because IL2-3 lies at the opening of the intracellular gate, it is likely involved in opening and closure of the gate.

The 4 constitutive and hyper-responsive mutations, F333S, S351T, V354L, and F358L, define a mutational hotspot at the intracellular face (Fig. 1) in which each of these substitutions favors a signaling conformation of Ssy1 (Poulsen et al. 2008). We propose that these mutations promote initial steps of opening the intracellular gate. This corresponds to a change of the equilibrium constant of reaction E3 to favor state D when ligand is bound and a similar change of E5 to favor state F when ligand is not bound (Fig. 2). Again, this rationalizes the constitutive and hyper-responsive phenotypes of these 4 mutations as found by Poulsen et al. (2008).

##### Interaction of the intracellular loop IL2-3 with a C-terminal latch

**Fig. 4.**
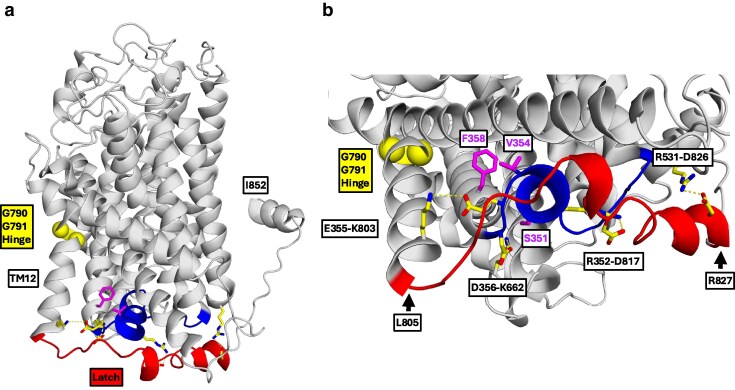
Interaction of a C-terminal latch with IL2-3 steered by a di-glycine hinge in TM12. a) Side view of an AlphaFold model of Ssy1 (Q03770) including residues 283 to 852 is shown in gray. The latch formed by the C-terminal residues L805 to R827 (red) is following TM12. The latch is adjacent to IL2-3 (L339 to D356) shown in blue. Residues S351 and V354 in IL2-3, and F358 in TM3, subject to gain-of-function mutations S351T, V354L, and F358L, are highlighted as magenta spheres. The G790-G791 hinge in the middle of TM12 is emphasized as yellow spheres. Residues potentially involved hydrogen bonds/salt bridges are shown as yellow sticks. The model has an outward-open conformation, in which the intracellular gate is closed. b) Close-up view highlighting potential hydrogen bonds/salt bridges (yellow dotted lines) between the latch and IL2-3. The involved residues are indicated in boxes. The side chains of S351, V354, and F358 are shown with magenta sticks. The model indicates the proximity of the gain-of-function mutations to the residues R352, E355, and D356, engaged in salt bridges/hydrogen bonds.

All Ssy1 orthologs (Supplementary Fig. 2) have a C-terminus of roughly 50 residues following TM12. The termini are evolutionarily constrained, with 6 fully conserved residues, D817, D819, G821, R822, D826, and W830 (Supplementary Fig. 2). The AlphaFold model of Ssy1 (Q03770), which is in an outward-open conformation (Supplementary Fig. 5), suggests that C-terminus residues D817 to W830 are positioned close to IL2-3 with the mutational hotspot (Fig. 4a). Importantly, AlphaFold has modeled most of these C-terminal residues with high confidence. The model suggests that the C-terminus interacts with the intracellular face of the Ssy1 TM bundle through 4 hydrogen bonds/salt bridges (Fig. 4b): (i) an E355–K803 salt bridge of 4.2 Å connecting IL2-3 and TM12; (ii) a D356–K662 H-bond of 2.7 Å connecting IL2-3 and IL8-9; (iii) an R352–D817 H-bond of 2.8 Å connecting IL2-3 and the C-terminus; (iv) an R531–D826 H-bond of 2.8 Å connecting TM7 and the C-terminus. The residues involved in the H-bonds/salt bridges are either functionally or fully conserved in the 37 Ssy1 orthologs (Supplementary Fig. 2), suggesting that the Ssy1 C-terminus is functionally important. In addition, the highly conserved I810 and L816 may interact with a hydrophobic cluster including L336, F353, V354, F358, L722, and F727 (not shown). We conclude that the outward-open conformation of Ssy1 is stabilized by attachment of the C-terminus to the TM bundle.

The constitutive mutations S351T, V354L, and F358L are neighbors of R352, E355, and D356, which are engaged in salt bridges/hydrogen bonds described above (Fig. 4b). We propose that the mutations weaken the R352–D817, E355–K803, and D356–K662 interactions and thereby favor a release of the C-terminus, resulting in a shift toward the inward-facing occluded, signaling conformation. We suggest that residues L805 to R827 form a latch (Fig. 4a) that tethers and untethers to the bottom of the TM bundle and that the release of the C-terminus triggers signaling. This would be reminiscent of a C-terminal latch in the *Drosophila* dopamine transporter dDAT and the human serotonin transporter hSERT which interacts with IL2-3 and modulates transporter activity (Penmatsa et al. 2013; Navratna and Gouaux 2019).

#### Mutation G790V likely modifies a hinge in the middle of TM12 involved in release of the C-terminal latch from the transmembrane bundle

The G790V mutation causes increased basal signaling and a lowered *EC*_50_ (Poulsen et al. 2008). G790 is located in the middle of TM12 (Figs. 1 and 4a). It is fully conserved in Ssy1 orthologs (Supplementary Fig. 2); in the 18 Ssy1 Saccharomycetaceae orthologs (Supplementary Fig. 8) both G790 and G791 are fully conserved. In contrast, neither of these 2 glycine residues are conserved in YAT family amino acid transporters (Supplementary Fig. 1). Glycine residues are often associated with kinks or bends in transmembrane helices, and the introduction of glycine residues in a transmembrane model helix induces local flexibility and H-bond destabilization, resulting in increased local bending and movements of the helix (Högel et al. 2018). We suggest that residues G790 and G791 provide a flexible hinge that helps keep Ssy1 in a nonsignaling conformation in the absence of extracellular amino acids.

A pronounced kink in the middle of TM12 was observed in the crystal structures of dDAT, hSERT, and hGlyT1 (Penmatsa et al. 2013; Coleman et al. 2016; Shahsavar et al. 2021) at 2 neighboring serine residues conserved in the neurotransmitter sodium symporter family (Cheng et al. 2017; Shahsavar et al. 2021). Models of Ssy1 based on the dDAT (4M48) and hSERT (5I6X) structures (Supplementary Table 2) show that G790-G791 in TM12 of Ssy1 aligns with the corresponding di-serine motif in dDAT and hSERT. Mutations in the hDAT di-serine motif, S567F and S568L, caused an increased dopamine transport activity (Cheng et al. 2017), reminiscent of the increased signaling of Ssy1-G790V. This is in accordance with the notion that the substitution of the helix-breaking glycine residue with the bulky, hydrophobic valine residue affects kinking at TM12 in Ssy1.

We propose that hinge-like movements around the G790-G791 motif in Ssy1 have a long-range effect on the position of the latch (Fig. 4). In conclusion, we suggest that the G790V mutation in Ssy1 moderates the kink feature and promotes disruption of interactions between the latch and the TM bundle, favoring a signaling conformation. This corresponds to a change of the equilibrium of reaction E3 to favor state D when ligand is bound, and similarly a change of E5 to favor state F when ligand is not bound (Fig. 2). This rationalizes the constitutive and hyper-responsive phenotypes of the G790V mutation, as found by Poulsen et al. (2008).

### The hypo-responsive mutation T639I likely affects conformational shifts of the TM1 loop

Amino acids have different potencies of signaling through Ssy1 (Bernard and André 2001; Gaber et al. 2003). Thus, the large neutral amino acids phenylalanine, isoleucine, and tryptophan are strong inducers. Leucine is by far the strongest, having an *EC*_50_ for Ssy1 of 12 µM (Poulsen et al. 2005a). We illustrate the binding of leucine in Ssy1 using a template structure of human LAT2 with bound leucine (7CMI; Yan et al. 2020). The model shows the well-established binding mode in amino acid transporters with a LeuT fold (Supplementary Fig. 9, Supplementary Table 3).

The T639I substitution on TM8 in Ssy1 (Bernard and André 2001) results in a hypo-responsive mutant with a strongly decreased apparent affinity for leucine, phenylalanine and l -norvaline (Poulsen et al. 2008). Thus, *EC*_50_ was increased by a factor of ca. 500 over that of the wild type for all 3 amino acids, suggesting that the T639I substitution does not markedly influence side chain binding but rather conformational equilibria leading to signaling. This may include the general binding mode of the α-amino and the α-carboxyl moieties of the sensed amino acid. Since T639I has the additional phenotype of a dramatically reduced basal level of signaling (Poulsen et al. 2008), the simplest a priori interpretation is that the mutant has unfavorable equilibria for shifts from an outward open to an occluded conformation of Ssy1, i.e. reactions E2 and E4 (Fig. 2).

To analyze how the T639I mutation might influence conformational changes or ligand binding, we used a ligand-bound outward-open Ssy1 model based on AdiC with bound arginine (3OB6; Kowalczyk et al. 2011; Supplementary Fig. 10). In AdiC, a substrate-induced fit of the TM1 loop appears to be required to promote the transition from an outward-open conformation to an occluded conformation (Kowalczyk et al. 2011), corresponding to reaction E2 (Fig. 2). According to the model (Supplementary Fig. 10), T639I does not affect hydrogen bonds to the ligand, but the isoleucine side chain is in contact with the TM1 loop. We propose that the hypo-responsive feature and the decreased basal level of signaling observed for the T639I mutant (Poulsen et al. 2008) are due to conformational constraints of the TM1 loop. This is likely inflicted by clashing between the side chains of I639 in TM8 and V297 in the TM1 loop, which is involved in substrate binding (Supplementary Fig. 10b).

### Interpretation of hyper-responsive and constitutive *PTR3* and *SSY5* mutants

Structures of Ptr3 and Ssy5 predicted by AlphaFold (Jumper et al. 2021) and evaluation of their evolutionary conservation among fungal species offer new opportunities to interpret how hyper-responsive and constitutive *PTR3* and *SSY5* mutations isolated by Poulsen et al. (2005b) and Poulsen et al. (2006) influence signaling, thereby suggesting signaling mechanisms.

#### Structural features of Ptr3

An analysis of disorder and structure of Ptr3 carried out using Metapredict and an AlphaFold model of Ptr3 (AF-P43606; Jumper et al. 2021) predicted confident structures of the N-terminal (R10 to T111) and C-terminal (E264 to K678) domains (Supplementary Fig. 11). The region G112 to D263 is predicted to be an IDR, except for an α-helix (L165 to A179) known as the LFA motif, involved in interaction between Ptr3 and Ssy5 (Omnus and Ljungdahl 2013). IDRs can bind to protein partners via coupled folding and binding (reviewed in Holehouse and Kragelund 2024). IDRs often function as platforms that interact with downstream signaling proteins, allowing their co-assembly and posttranslational modifications (Holehouse and Kragelund 2024).

An alignment of 32 Ptr3 orthologs shows 2 blocks of conserved residues in their N-termini corresponding to *S. cerevisiae* Ptr3 residues V49 through F66 and C80 through A101 (Supplementary Fig. 12). The AlphaFold structure of residues R10 to T111 reveals 4 highly conserved cysteine residues (C57, C59, C80, and C83) that are spatially clustered with a distance of ∼3.5 Å between their sulfur atoms, which would allow binding of a metal ion (Supplementary Fig. 11c).

**Fig. 5.**
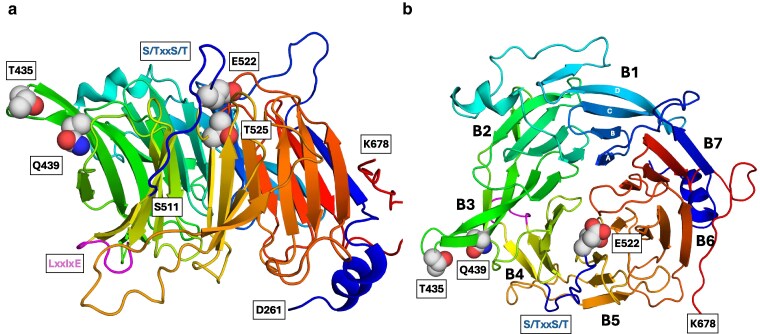
AlphaFold model of Ptr3 showing positions of mutations and the Rts1 interaction motif and the Yck phosphorylation motifs. a) AlphaFold model of Ptr3 (P43606) showing residues from D261 to K678, which fold into a WD40-repeat structure consisting of a 7-bladed β-propeller. Helices and loops are colored blue to red starting with residue D261. Residues T435 and Q439, subject to gain-of-function mutations T435K and Q439R, are indicated as gray spheres. They are surface exposed in blade B3 on the top side of Ptr3; T435 is in the loop between β-sheets B and C, and Q439 in β-sheet C. Residues E522 and T525 (cyan spheres), subject to loss-of-function mutations E522K and T525A are also surface exposed on the top side. E522 is in the loop between β-sheet D in blade 4 and β-sheet A in blade 5, and T525 is the first residue in β-sheet A of blade 5. The potential phosphorylation sites 511-SVRS-514, 514-SIDT-517, and 518-SLES-521 in the loop between blade B4 and blade B5 are highlighted in blue. The proposed Rts1-binding LxxIxE motif (L499-E504, magenta) is in the bottom of the WD40 repeat in the loop between β-sheets C and D in blade B4. b) Top-view showing blades numbering (B1 through B7) and β-sheet nomenclature (A through D).

The AlphaFold model of Ptr3 shows the C-terminal part of Ptr3 (E264 to K678) with a structure characteristic of WD40-repeat proteins (Xu and Min 2011; Jain and Pandey 2018), consisting of a 7-bladed propeller with each blade comprising a 4-stranded antiparallel β-sheet (Fig. 5).

#### Ptr3 gain-of-function and loss-of-function mutations located on the top side of the WD40 repeat

Hyperphosphorylation of Ptr3 is induced by extracellular amino acids in an Ssy1- and Yck-dependent manner, but independently of Ssy5 (Liu et al. 2008). The gain-of-function mutation *PTR3*-Q439R (Poulsen et al. 2005b) causes hyperphosphorylation of Ptr3 in the absence of inducer, whereas hyperphosphorylation is blocked in a loss-of-function mutant, *ptr3*-T525A (Liu et al. 2008).

Residues T435 and Q439, subject to the gain-of-function mutations T435K and Q439R (Poulsen et al. 2005b), are surface exposed in blade 3 on the top side (convention of Xu and Min 2011) of the WD40 repeat (Fig. 5). Residues corresponding to T435 are conserved as either threonine or serine in Ptr3 orthologs (Supplementary Fig. 13), and Q439 is strictly conserved. The strictly conserved residues E522 and T525 (Supplementary Fig. 13), subject to loss-of-function mutations E522K (*ptr3-35*, Abdel-Sater et al. 2004, B. André, personal communication) and T525A (Liu et al. 2008), are also located at the top face of the WD40 structure, but in blade 5 (Fig. 5).

#### Phosphorylation of Ptr3 by protein kinase Yck

Casein kinases Yck1 and Yck2 are positive, redundant factors in SPS sensing (Abdel-Sater et al. 2004; Spielewoy et al. 2004), required for hyperphosphorylation of Ptr3 (Liu et al. 2008). The role of phosphorylation of Ptr3 in signaling is poorly understood, and the phosphorylation sites (phosphosites) in Ptr3 are unknown. We therefore looked for conservation of the Yck substrate motif (S/T)XX(S/T) (Mok et al. 2010) in a collection of 46 fungal orthologs of Ptr3 (Supplementary Fig. 13). We identified 3 rather conserved phosphorylation motifs, 511-SVR**S**-514, 514-SID**T**-517, and 518-SLE**S**-521 (proposed phosphorylated residues indicated in bold, *S. cerevisiae* Ptr3 numbering). These phosphosites are placed in the loop between β-sheet D in blade 4 and β-sheet A in blade 5 on the top face of the WD40-repeat structure (Fig. 5). The phosphosites neighbor T525, subject to the T525A mutation, which results in the inability of Ptr3 to become hyperphosphorylated. This closeness accords with this phenotype, and E522K could well have the same effect.

Yck is tethered to the plasma membrane through 2 palmitoyl moieties attached to residues C537 and C538 in the C-terminal domain of Yck by the Golgi-localized palmitoyl-transferase Akr1 (Vancura et al. 1994; Roth et al. 2002, 2011; Babu et al. 2004; Pasula et al. 2010). *S. cerevisiae*  Yck1 has a typical casein kinase 1 structure (Xu et al. 1995) according to its X-ray structure (5X18; Shinohara et al. 2017) and the AlphaFold structure (P23291). A structured ∼300 residues part close to the N-terminus thus comprises 2 subdomains that bind ATP and the protein substrate, respectively (Supplementary Fig. 14b). This catalytic domain and the C-terminal palmitoylation motif are separated by a 176-residue-long, poorly conserved, partly disordered, glutamine-rich sequence (Roth et al. 2011). This long, flexible linker may facilitate attachment of a substrate protein to the kinase domain within a suitable distance from the plasma membrane. The AlphaFold structure and a Metapredict analysis of Yck1 predict that the glutamine-rich stretches form 2 long α-helices (Supplementary Fig. 14a and -b); such helices mediate protein–protein interactions in protein complexes (Schaefer et al. 2012). Two-hybrid analysis has shown that the Ssy1 N-terminal domain interacts with Yck, Ptr3, and Ssy5, whereas interaction between Ptr3 and Yck1 failed detection (Liu et al. 2008). It is believed that binding of a ligand to Ssy1 triggers a physical interaction between Ssy1-bound Ptr3 and Yck (Liu et al. 2008). We therefore suggest that the plasma membrane-anchored linker region of Yck1 acts as a long, flexible arm that enables binding of the kinase domain to the Ssy1 N-terminus and allows phosphorylation of Ptr3 and Ssy5.

#### Dephosphorylation of Ptr3 by protein phosphatase 2A

Rts1 is a regulatory subunit of protein phosphatase PP2A and is needed to downregulate SPS signaling in the absence of extracellular amino acids (Eckert-Boulet et al. 2006; Liu et al. 2008; Omnus and Ljungdahl 2013). Deletion of *RTS1* leads to hyperphosphorylation of Ptr3 (Liu et al. 2008).

Because Rts1 interacts with Ptr3 (Breitkreutz et al. 2010), we looked in Ptr3 for the LxxIxE motif, which has been identified in proteins that interact with the regulatory subunit B56 of the human protein phosphatase 2A (PP2A)–B56 complex (Hertz et al. 2016; Wang et al. 2016). The LxxIxE motifs, which are present throughout Eukarya, bind to a conserved surface-exposed pocket on B56 subunits (Hertz et al. 2016; Wang et al. 2016). Remarkably, human B56α interacts with *S. cerevisiae* Mad3, which has an LxxIxE motif (Hertz et al. 2016). Vice versa, Rts1, which is an ortholog of human B56, also binds human proteins having LxxIxE motifs (Hertz et al. 2016). We found the (L/F/M)xx(I/V/L)xE consensus motif (Hertz et al. 2016) at 4 places in Ptr3, of which 3 are highly conserved. Two of these, 309-FALITE-314 and 570-LIRVDE-575 (Supplementary Fig. 13), appear in structured parts and are not very exposed. The 499-LQKINE-504 Rts1 motif, however, is placed in a surface displayed loop on the bottom face of the WD40 repeat between β-sheets C and D in blade 4 (Fig. 5). We therefore propose that Rts1 and Ptr3 interact through this motif.

Although AlphaFold 3 could not confidently model binding between Ptr3 and PP2A, we suggest that in the absence of extracellular amino acids the regulatory subunit Rts1 of PP2A^Rts1^ is kept bound to the 499-LQKINE-504 motif of Ptr3. This would allow Pph21, the catalytic subunit of PP2A, to keep the S511-S521 phosphosites of Ptr3 (Fig. 5) de-phosphorylated. We suggest that binding of amino acids to Ssy1 results in a swing-out of its N-terminal domain, facilitating access of Yck to Ptr3, resulting in hyperphosphorylated Ptr3. This scenario is consistent with the pattern of interactions suggested by Omnus and Ljungdahl (2013), including 2-hybrid Rts1–Ssy5 interaction and their evidence that Ptr3 mediates regulated contact between Yck and Ssy5.

#### Structural features of Ssy5

The cleavage site between the prodomain and the catalytic domain of Ssy5 is between residues A381 and A382 (Poulsen et al. 2006). Metapredict and AlphaFold predict that a large part of the prodomain, including residues 1 to 164, is disordered, whereas residues 165 to 699, comprising 119 residues of the prodomain and the full catalytic domain, are structured (Supplementary Fig. 15a). The catalytic triad conserved in chymotrypsin-like serine proteases (H465, D545, and S640) is placed close to the A381/A382 cleavage site (Supplementary Fig. 15b).

#### Phosphorylation and dephosphorylation of Ssy5

A region of the Ssy5 prodomain (residues 60 to 90) contains potential phosphosites important for triggering degradation of the prodomain (Abdel-Sater et al. 2011; Omnus et al. 2011). To identify possible phosphosites, we analyzed the prodomain for Yck substrate motifs, (S/T)xx(S/T) (Mok et al. 2010). We found 15 phosphorylation motifs in the *S. cerevisiae* sequence, 5 of which, 67-SIQ**S**-70, 72-SIF**S**-75, 80-TYG**T**-83, 83-TGA**S**-86, and 88-SMA**T**-91 (*S. cerevisiae* numbering, phosphorylated residue bold) are in the important phosphosites region and highly conserved in an alignment of 18 Ssy5 orthologs (Supplementary Fig. 16). It is therefore likely that a number of these serine and threonine residues are phosphorylated by Yck.

We looked for conserved Rts1-binding (L/F/M)xx(I/V/L)xE motifs in Ssy5 (Supplementary Fig. 16). One conserved sequence, 177-LSELEE-182, is buried in a structured part and is unlikely to bind Rts1; the other motif, 126-LSPVKE-131 in *S. cerevisiae*, is in the disordered part of the prodomain and is located in identical or similar positions in 17 of 18 Ssy5 orthologs examined. That this is most likely a binding site for Rts1 is supported by constitutive *SSY5* mutations in positions L126, V129, and E131 identified in 2 independent screens (Poulsen et al. 2006; Pfirrmann et al. 2010). The fact that these mutations affect the exact positions of the LxxIxE motif that are important for its interaction with Rts1 supports our interpretation that they prevent or reduce Rts1 interaction.

**Fig. 6.**
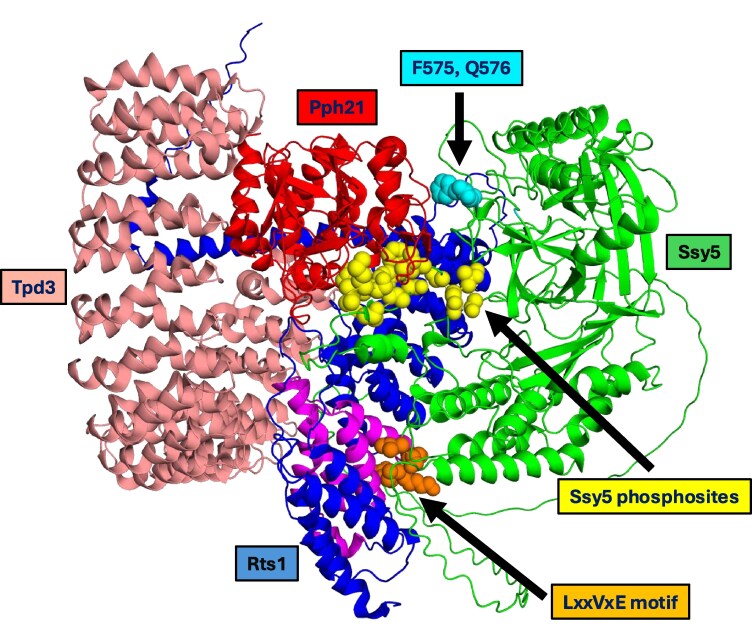
Interactions between Ssy5 and PP2A. AlphaFold 3 analysis of full-length Ssy5 and structured parts of yeast PP2A (ipTM = 0.58, pTM = 0.63). Full-length Ssy5 is colored green. Putative phosphosites in Ssy5 (67-SIQS-70, 72-SIFS-75, 80-TYGT-83, 83-TGAS-86, and 88-SMAT-91) and the LxxVxE motif (125-LSPVKE-131) are highlighted as yellow and orange spheres, respectively. Residues F575 and Q576, subject to gain-of-function mutations F575V and Q576P, in the catalytic domain of Ssy5 are shown as cyan spheres. The structured parts of the PP2A subunits are shown colored as follows: catalytic subunit Pph21 (N63-Q348, red), scaffold subunit Tpd3 (S30-N635, rosa), and regulatory subunit Rts1 (N281-Q757, blue). The HEAT3-HEAT4 region of Rts1 (H401-A485) is emphasized in magenta.

We applied AlphaFold 3 (Abramson et al. 2024) to study interactions between Ssy5 and the yeast PP2A complex. Using PP2A subunits (Rts1, Pph21, and Tpd3) lacking the IDRs, a confident PP2A model was obtained (ipTM = 0.86, pTM = 0.86). We used this model to analyze PP2A interaction with full-length Ssy5, containing the phosphosites and the Rts1-binding site in its disordered N-terminal; i.e. residues 1 to 164 (Supplementary Fig. 15a). AlphaFold 3 yielded a low-confidence interaction model (ipTM = 0.58, pTM = 0.63), most likely due to the disordered N-terminal region of the Ssy5 prodomain. Nevertheless, the Rts1-binding motif in Ssy5 (126-LSPVKE-131) was placed adjacent to the groove formed by HEAT3-HEAT4 helices (R426-G440 and E472-G483) in Rts1 (Fig. 6), known to be involved in binding LxxIxE interactors (Hertz et al. 2016; Wang et al. 2016). The interactions between the LxxVxE sequence and the HEAT3-HEAT4 region fit perfectly with the binding pattern found for a crystal structure of B56γ and BubR1 interactions (Wang et al. 2016), with full conservation of the 12 interacting residues when comparing yeast Rts1 to human B56γ (Supplementary Fig. 17).

In addition, AlphaFold 3 places the suggested 5 phosphosites in Ssy5 into the catalytic cleft of Pph21 (Fig. 6). The phosphosites 80-TYGT-83 and 83-TGAS-86 are placed along the hydrophobic and acidic groove of Pph21 (Supplementary Fig. 18). This positioning resembles overall the binding of a Cdc37 peptide to human PP5 (5HPE) and Inhibitor-2 to rodent PP1 (2O8G), as well as the binding of various toxin inhibitors to phosphoprotein phosphatases (Oberoi et al. 2016; Lemaire and Bollen 2020). The side chain of Ssy5-T83 is docked close to the Pph21 active site residues D117, H119, D145, N177, H178, H227, and H301. They are highly conserved within the phosphoprotein phosphatase (PPP) family of serine/threonine phosphatases and known to coordinate 2 metal ions (Mn^2+^ and Fe^2+^) involved in the catalytic mechanism (Shi 2009; Oberoi et al. 2016; Supplementary Fig. 18b). In addition, residue Ssy5-T83 is flanked by residues R149 and R274, which are strictly conserved in the PPP superfamily and involved in the dephosphorylation mechanism (Oberoi et al. 2016).

Despite the low overall confidence metrics of the AlphaFold 3 PP2A–Ssy5 model, the interactions of the phosphosites and of the LxxVxE motif with PP2A described above are consistent with known PP2A–substrate interactions (Supplementary Figs. 17 and 18). In addition, the local per-atom confidence estimates (pIDDT; Supplementary Fig. 19) indicate confident structures of the interacting parts. We conclude that this tentative Ssy5–PP2A model is a useful basis for future experimental studies.

Moreover, Ssy5 residues F575 and Q576, subject to the gain-of-function mutations F575V and Q576P, are adjacent to a hydrophobic face in the catalytic subunit Pph21; i.e. residues W269 and F278 in antiparallel β-sheets β8 and β9, respectively, and to V284 in the neighboring α9-helix (nomenclature according to Xu et al. 2006; Supplementary Fig. 17c). An obvious possibility is that these mutants are constitutive by preventing proper binding of Ssy5 to PP2A, thereby preventing sufficient dephosphorylation. In addition, the F575V and Q576P mutants are hyper-responsive, suggesting that they affect the global conformational dynamics of the SPS complex in a way that changes one or both of the equilibrium constants of reactions E2 and E3 (Fig. 2), favoring the signaling Ssy1 state D. We therefore propose that PP2A is an integral part of an SPS–PP2A complex, at least in the nonsignaling state.

### Features of the cytoplasmic N-terminal domain of Ssy1

The N-terminal domain of Ssy1 interacts directly with Yck, Ptr3, and Ssy5 to execute and control signal transduction, and important aspects of these interactions have been revealed (Liu et al. 2008; Omnus and Ljungdahl 2013). Below, we use bioinformatic tools to explore structural features of the N-terminal domain and its protein interactions.

#### Intrinsic disorder

**Fig. 7.**
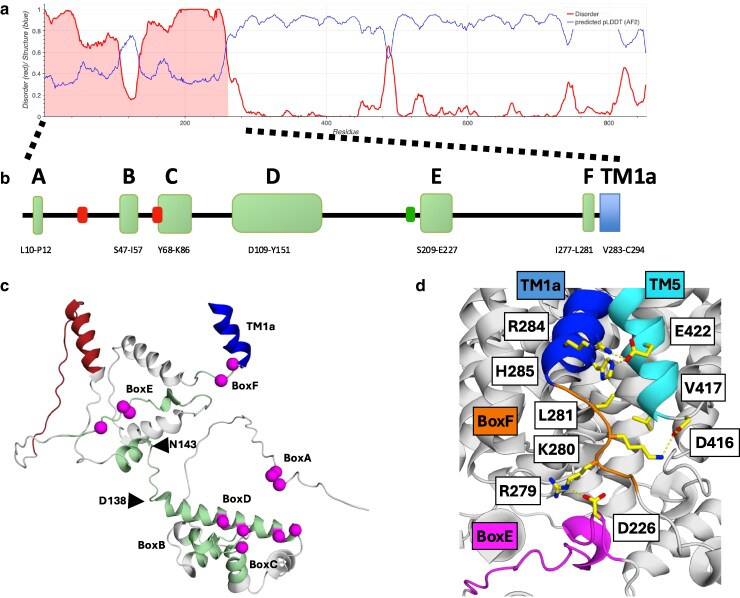
The cytoplasmic N-terminal domain of Ssy1. a) Intrinsic disorder (red curve) and per-residue model confidence score (pLDDT; blue curve) were analyzed using the Metapredict (v2.4) web server (Emenecker et al. 2021). b) Conserved regions in the cytoplasmic N-terminal domain of Ssy1 identified in Saccharomycetaceae Ssy1 orthologs (Supplementary Fig. 7). Regions with conserved residues and residues having similar physico-chemical properties are shown schematically as green boxes designated A through F. TM1a is shown as a blue box. Sequence coordinates in *S. cerevisiae* Ssy1 numbering are shown below the boxes. Insertion of hemagglutinin epitopes at peptide bond 34/35 or at 68/69 yielded nonfunctional Ssy1s (red squares), whereas insertion at 206/207 yielded a functional Ssy1 (green square; Klasson et al. 1999). c) Overall structure of the N-terminal domain of Ssy1 predicted by AlphaFold (Q03770), showing partially disordered subdomain 1 (M1 to N137) and subdomain 2 (E144 to K282), connected by a short loop (D138-N143). BoxA (L10-P12), BoxB (S47-I57), BoxC (Y68-K86), BoxD (D109-Y151), BoxE (S209-E227), and BoxF (I277-L281) are highlighted in green. Fully conserved residues are indicated by magenta spheres: (i) L10, F11, and P12 in Box A; (ii) F77 in Box C; (ii) F110, Y113, E120, and R124 in BoxD; (iii) T212, I215, and D216 in BoxE; (iv) R279 and L281 in BoxF. A dispensable region (Ring et al. 2019; S167-N196, firebrick) and TM1a (V283 to C294, blue) are also indicated. d) Interactions of the BoxF-TM1a region with TM5 and BoxE shown in a close-up view of the AlphaFold Ssy1 model. Residues in BoxF (orange) and TM1a (blue) interacting with residues in IL4-5, TM5 (cyan) and BoxE (magenta) are highlighted: (i) K280 and D416 form a 3.5 Å salt bridge; (ii) L281 and V417 form a hydrophobic interaction; (iii) E422 forms hydrogen bonds to R284 and H285 of 2.6 and 2.7 Å, respectively; (iv) D226 and R279 form a 3.1 Å hydrogen bond. Interacting side chains are shown as yellow sticks.

Metapredict analysis of the Ssy1 sequence identified IDRs comprising residues 1 to 108 and 135 to 260 in the N-terminal domain (Fig. 7a). They may well be key to the interactions with Yck, Ptr3, and Ssy5, due to the ability of IDRs to function as platforms for interaction with downstream signaling proteins (Holehouse and Kragelund 2024).

#### Conserved sequence boxes

To look for sequence elements within the N-terminal domain of Ssy1 that may mediate interactions with Yck, Ptr3 and Ssy5, a set of 18 closely related Saccharomycetaceae Ssy1 orthologs were aligned (Supplementary Fig. 20). The alignment revealed sequences with conserved and partially conserved residues, designated BoxA through BoxF (Fig. 7b). Thirteen residues within the boxes are fully conserved, suggesting that they are critical for function (Fig. 7c and Supplementary Fig. 20).

#### Structural features of the N-terminus

AlphaFold proposes a model of the Ssy1 N-terminal domain (UniProt ID: Q03770; Supplementary Fig. 21) containing 2 subdomains connected by a short loop, D138-N143 (Fig. 7c). Subdomain 1 includes residues from M1 to N137, where the structure of residues S47 to N137 is predicted with confidence. It contains BoxB, BoxC, and BoxD, which have 5 fully conserved residues (F77, F110, Y113, E120, and R124) potentially involved in protein interactions (Fig. 7c). Subdomain 2 includes residues from E144 to K282, for which most of the structure is predicted with low confidence. BoxE and BoxF are part of this unit, but only BoxF, which neighbors TM1a, has a confident structure.

Although intrinsic disorder dominates the N-terminal domain (Fig. 7a), its overall architecture is clearly important for function. Thus, Klasson et al. (1999) found that insertion of a hemagglutinin epitope reiterated 3 times (HA3) into the Ssy1 N-terminal between residues 34 and 35 or between residues 68 and 69 yielded nonfunctional versions of Ssy1 (Fig. 7b). Perhaps this reflects interference with proper binding of Ptr3, Ssy5, and/or Yck1 to subdomain 1 (M1 to N137; Fig. 7c). Insertion of HA3 between residues 206 and 207, on the other hand, yielded a functional Ssy1. This agrees with the variability of the region between P156 and S209 in fungal Ssy1 orthologs (Supplementary Figs. 2 and 20) and the finding that deletion of a region between BoxD and BoxE (ΔS167-N196) did not affect SPS-sensor function (Ring et al. 2019).

The AlphaFold model suggests that the BoxF-TM1a region (K280 to R284) interacts with IL4-5 and TM5 (D416, V417, and E422) at several places (Fig. 7d): (i) K280 and D416, which form a hydrogen bond, are highly conserved in Ssy1 orthologs (Supplementary Fig. 2), but absent in bona fide YAT transporters (Supplementary Fig. 1); (ii) residues L281 and V417, which form a hydrophobic interaction, are highly conserved in both Ssy1 orthologs and YATs; (iii) R284 and H285 form hydrogen bonds to E422 and are fully conserved in both Ssy1 orthologs and YATs. We suggest that the overall function of these interactions in Ssy1 is to prevent a swing-out of TM1a in nonsignaling conformations. Binding a ligand to the substrate pocket of Ssy1 would expectedly cause disruption of these interactions and facilitate a swing-out of TM1a leading to disruption of the D226-R279 salt bridge (Fig. 7d), which connects BoxE to the TM bundle. In addition, substrate-induced TM5 movement away from the inner portion of the TM bundle is broadly important to open the intracellular gate (Licht et al. 2024). This series of events may allow contact between Yck bound to the plasma membrane and Ptr3 and Ssy5 attached to the Ssy1 N-terminal.

### Interactions in the SPSsensor complex

Several interactions between SPS-sensor components have been revealed by yeast 2-hybrid analysis (Bernard and André 2001; Liu et al. 2008; Omnus and Ljungdahl 2013). To obtain likely details on the interactions, we made structure predictions using AlphaFold 3 (Abramson et al. 2024). The AlphaFold Server recommends various confidence metrics to check the accuracy of the predicted structures. In short, a predicted template-modeling (pTM) score above 0.5 means that the overall predicted fold for the complex might be similar to the true structure. The interface pTM (ipTM) score measures the accuracy of the predicted relative positions of the subunits within the complex. ipTM values >0.8 represent confident high-quality predictions, while values below 0.6 suggest likely a failed prediction. ipTM values between 0.6 and 0.8 are a gray zone where predictions could be correct or incorrect.

#### Interaction between the LFA motif in Ptr3 and the prodomain of Ssy5

Interactions between residues 151 to 678 of Ptr3 and residues 160 to 699 of Ssy5 were analyzed, thereby disregarding disordered regions, yielding a higher confidence of prediction (ipTM = 0.8, pTM = 0.79; Supplementary Fig. 22a). AlphaFold 3 places the Ssy5 prodomain adjacent to the bottom face of the WD40 domain of Ptr3. Remarkably, the α-helix (L165-A179) in Ptr3 (Supplementary Fig. 11a and b) containing the LFA motif (Omnus and Ljungdahl 2013) protrudes from the disordered N-terminal region of Ptr3 and makes interactions with the Ssy5 prodomain. Thus, Ptr3 residues L165, L166, **L**168, **F**169, V172, and **A**173 within the LFA motif (bold characters) are predicted to interact with Ssy5 residues I308, L309, I319, I320, A326, I328, I344, F346, and L348 (Supplementary Fig. 22b). The per-atom confidence estimate (pIDDT) of the interacting Ptr3 and Ssy5 residues is confident (plDDT between 70 and 90) or very high (plDDT > 90), suggesting local correctness of the model (Supplementary Fig. 22c).

AlphaFold 3 places F575 and Q576 at the surface of the catalytic subunit of Ssy5 (Supplementary Fig. 22a). This agrees with the suggestion that the gain-of-function mutations F575V and Q576P prevent proper binding of Ssy5 to PP2A and thus lower the level of dephosphorylation (Fig. 6).

#### Interactions between blade 3 in Ptr3 and BoxD and BoxE in Ssy1

AlphaFold 3 analysis of structured Ptr3 residues (260 to 678) and the Ssy1 N-terminal domain (residues 1 to 283) yielded a model (Supplementary Fig. 23) with reliable confidence results (ipTM = 0.81 and pTM = 0.65; Supplementary Fig. 23e and f). According to the model, gain-of-function *PTR3* mutations T435K and Q439R neighbor highly conserved Ssy1 residues L142, L147, and Y151 in BoxD as well as the strictly conserved T212, I215, and D216 in BoxE (Supplementary Fig. 23b). The BoxD residues may form hydrophobic interactions with conserved Ptr3 residues I428 and L441 in β-sheet B and β-sheet C, respectively, of blade 3 (Supplementary Fig. 23c). The BoxE residues are aligned with Ptr3 residues in the loop connecting blade 3 and blade 4 (Supplementary Fig. 23d). Thus, conserved BoxE residues (V214, I215, and D216) and conserved Ptr3 residues (P467, Y468, and R469) in the loop following β-sheet D in blade 3 may participate in a partially hydrophobic cluster. Combined with a hydrogen bond between Ssy1-D216 and Ptr3-Y468, a tight interaction between BoxE and Ptr3 may be formed. The pIDDT estimates of the interacting Ssy1 and Ptr3 residues are confident or highly confident, suggesting local correctness of the model (Supplementary Fig. 23e).

Mutations T435K and Q439R in blade 3 of Ptr3 are close to the proposed Ssy1–Ptr3 interaction face. This supports the notion that substitutions of residues T435 and Q439 with lysine and arginine, respectively, introduce repulsion with the neighboring R469 side chain, distorting local Ptr3 structure (Supplementary Fig. 23d), thereby facilitating release of Ptr3 from Ssy1 and promotion of the signaling process.

#### Interaction between BoxA in Ssy1 and the catalytic domain of Ssy5

AlphaFold 3 analysis of structured Ssy5 residues (160 to 699) and the Ssy1 N-terminal domain (residues 1 to 283; Supplementary Fig. 24) yielded a reliable model (ipTM = 0.79 and pTM = 0.67). It suggests that strictly conserved BoxA residues in Ssy1 (Fig. 7c and Supplementary Fig. 20) interact with the catalytic domain of Ssy5. Thus, BoxA residues L10, F11, and P12 may insert into a hydrophobic pocket formed by Ssy5 residues V532, W533, I548, I689, and W691 (Supplementary Fig. 24). The pIDDT estimates of the interacting Ssy1 and Ssy5 residues are confident or highly confident, suggesting local correctness of the model (Supplementary Fig. 24c).

#### SPS-sensor model in a nonsignaling state

**Fig. 8.**
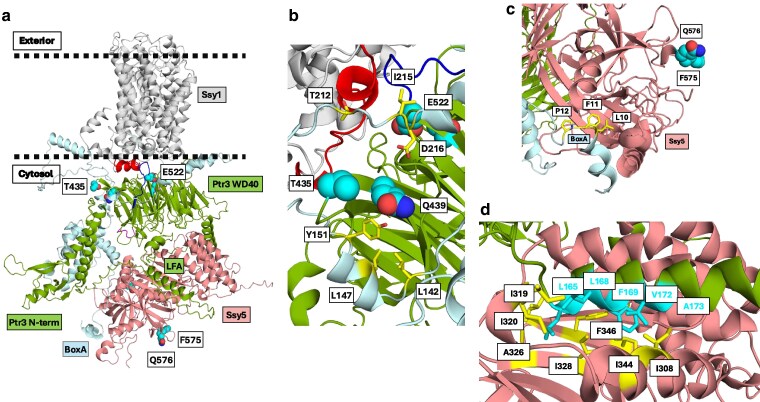
Interactions in the Ssy1–Ptr3–Ssy5 complex. a) AlphaFold 3 model of full-length Ssy1, Ptr3, and Ssy5. The various components are colored: Ssy1 N-terminal domain (pale cyan), Ssy1 TM bundle (gray), Ptr3 (split pea), and Ssy5 (salmon). Key residues are highlighted as cyan spheres, ie T435 and Q439, subject to gain-of-function *PTR3* mutations, E522 and T525, subject to loss-of-function *ptr3* mutations, and F575 and Q576, subject to gain-of-function *SSY5* mutations. The Ssy1 C-terminal latch (L813-W830) is colored red. Proposed phosphorylation motifs in Ptr3 (S511 to S521) are colored blue. The disordered region M1-E159 in Ssy5 has been hidden for clarity. b) Close-up of the interaction faces of BoxD and BoxE of Ssy1 and blade 3 of Ptr3. Ssy1 residues T212, I215 and D216 in BoxE and L142, L147, and Y151 in BoxD are highlighted as yellow sticks and neighboring Ptr3 residues T435 and Q439 as cyan spheres. c) Close-up of the interaction faces of BoxA of Ssy1 and the catalytic domain of Ssy5. Ssy1 residues L10, F11, and P12 in BoxA are shown as yellow sticks. d) Close-up of the interaction faces of the LFA motif of Ptr3 and the prodomain of Ssy5. Ptr3 residues L165, L166, L168, F169, V172, and A173 in the LFA motif are shown as cyan sticks. Potentially, they interact with a hydrophobic face of the Ssy5 prodomain including residues I308, L309, I319, I320, A326, I328, F346, and L348 shown as yellow sticks.

An AlphaFold 3 analysis of the structure of the SPS sensor consisting of full lengths of Ssy1, Ptr3, and Ssy5 yielded a heterotrimer with low-confidence parameters (ipTM = 0.46 and pTM = 0.53; Fig. 8a). This is likely due to disordered regions in all 3 subunits (Fig. 7a, Supplementary Figs. 11 and 15), as reflected in pIDDT estimates (Supplementary Fig. 25). Whereas structured parts of the 3 subunits are confident, disordered regions have low confidence, as expected. Nevertheless, the analysis predicts an SPS-sensor complex with interactions that are very similar to the high-confidence AlphaFold 3 models of heterodimers Ptr3–Ssy5, Ptr3–Ssy1S, and Ssy5–Ssy1 discussed above (Supplementary Figs. 22–24). Thus, the heterotrimer sensor model (Fig. 8a) supports the following interactions: (i) BoxD and BoxE in Ssy1 interact with the WD40 domain of Ptr3 (Fig. 8b); (ii) BoxA in Ssy1 interacts with the catalytic subunit of Ssy5 (Fig. 8c); (iii) the LFA domain of Ptr3 interacts with the prodomain of Ssy5 (Fig. 8d); (iv) the gain-of-function *SSY5* mutations F575V and Q576P are placed at the surface of Ssy5 opposite the Ptr3-interacting face and likely reduce binding of PP2A to Ssy5 (Figs. 6 and 8a and Supplementary Fig. 22a).

In conclusion, we regard this AlphaFold 3 model of full-length Ssy1, Ptr3, and Ssy5 as the currently best working model of the SPS sensor. We emphasize that it is a provisional model, needing experimental validation. However, we find it useful for an overview of interactions between the subunits and for planning future experiments.

#### Interaction between Yck and BoxD in Ssy1

Yck1 interacts with the N-terminal domain of Ssy1 (Liu et al. 2008). To identify Yck1–Ssy1 interaction faces we made an AlphaFold 3 analysis comprising the Ssy1 N-terminal domain (residues 1 to 282), the structured part of Yck1 (residues 61 to 350, see Supplementary Fig. 14a), and an ATP molecule. This yielded a confident model (ipTM = 0.81 and pTM = 0.67; Supplementary Fig. 26). According to the model, strictly conserved Ssy1 residues F110 and Y113 in BoxD (Supplementary Fig. 20) are within range of forming hydrophobic interactions and/or pi–-pi interactions with strictly conserved Yck1 residues F84 and F99 (Supplementary Fig. 27) in β-sheets β2 and β3, respectively, of the N-terminal lobe of Yck1 (Supplementary Fig. 26b). The reliability of the interactions is supported by pIDDT estimates (Supplementary Fig. 26c and -d).

These results suggest that Yck binds to BoxD residues F110 and Y113. Similarly, results described above suggest that Ptr3 also binds to BoxD, but at residues L142, L147, and Y151. The ability of Ssy1 to bind Yck and Ptr3 in adjacent positions supports the notion (Liu et al. 2008; Omnus and Ljungdahl 2013) that binding of ligand to Ssy1 initiates conformational changes of Ssy1 that bring Ptr3, bound to the Ssy1 N-terminus, in contact with plasma membrane-bound Yck. We suggest that Ptr3 becomes hyperphosphorylated by Yck as a result of this series of conformational events.

#### Phosphorylation of Ptr3 and its displacement from Ssy1

The SPS-sensor model (Fig. 8a), which is in an outward-open, nonsignaling conformation, suggests new mechanistic details of the signal transduction process taking place at the Ssy1–Ptr3 interface: (i) As discussed above, the constitutively signaling *PTR3* mutations T435K and Q439R may reduce Ptr3 interactions with BoxD and BoxE in Ssy1 in a way that promotes signaling (Fig. 8b). Accordingly, we suggest that the conformational changes induced by ligand-bound Ssy1 involve interactions in the Ptr3–Ssy1 interface, leading to a rotational displacement of Ptr3 from Ssy1. (ii) The Ptr3 phosphosites region (S511-S521) and the neighboring mutations E522K and T525A, believed to block hyperphosphorylation of Ptr3, are placed by AlphaFold 3 close to the Ssy1 C-terminal latch (L809-W830; Fig. 8a and Supplementary Fig. 28b). We interpret the low-confidence score of the Ptr3 phosphosites and the Ssy1 latch (Fig. 8a, Supplementary Fig. 29e, and 29f) to reflect flexible and adaptive features expected for these regions.

In the nonsignaling SPS complex, with the outward-open conformation of Ssy1, the 3 conserved phosphosites in Ptr3 are buried within the structure and therefore inaccessible to phosphorylation by Yck (Supplementary Fig. 28c). We propose that the conformational changes associated with ligand binding to Ssy1 include a large displacement of Ptr3 from Ssy1 that allows access of plasma membrane-bound Yck to the phosphosites, leading to phosphorylation of Ptr3 residues S514, T517, and S521. The introduced phosphate groups neighbor the highly conserved residues D814, D817, D819, E824, and D826 in the C-terminal latch of Ssy1 (Supplementary Fig. 29). We suggest that repulsion between the negatively charged phosphate groups in Ptr3 and the side chains of the aspartate and glutamate residues in the Ssy1-latch results in further release of Ptr3 from Ssy1. This notion is supported by AlphaFold 3 analysis of Ssy1 and Ptr3 variants having various combinations of phosphorylations of S514, T517, and S521. The analysis suggests a large rotational displacement of phosphorylated Ptr3 away from Ssy1 (Supplementary Fig. 30).

#### Ptr3–Ptr3 interactions

Because of the likely importance of Ptr3 dimerization in SPS signaling (Bernard and André 2001; Liu et al. 2008; Omnus and Ljungdahl 2013), we explored Ptr3–Ptr3 interaction using AlphaFold 3. The analysis was only able to measure confident interactions between 2 copies of the N-terminal, comprising residues 1 to 110 predicted to be structured (Supplementary Fig. 11a). The resulting dimer had high-confidence parameters (ipTM = 0.81 and pTM = 0.82) and exhibited a comprehensive interaction pattern (Supplementary Fig. 31) that may explain Ptr3–Ptr3 interaction.

The importance of Ptr3 dimerization in SPS signaling is poorly understood and could imply dimerization of the full SPS complex. Examples of transporter oligomerization within the APC family include LeuT (Yamashita et al. 2005), AdiC (Ilgü et al. 2021), and monoamine transporters (Paul and Shukla 2024).

We propose that a rotational displacement of Ptr3 away from Ssy1 (Supplementary Fig. 27) and subsequent hyperphosphorylation of Ptr3 are prerequisites for homodimerization of Ptr3. This is in accordance with the finding that the loss-of-function mutation *ptr3*-T525A, which blocks hyperphosphorylation of Ptr3, abolishes the ability of Ptr3 to interact with itself (Omnus and Ljungdahl 2013). It is conceivable that Ssy5 is still attached to Ptr3 when Ptr3 dimerizes, so that a Ptr3–Ssy5 heterotetramer is formed; this could offer a steric situation that allows phosphorylation of Ssy5 by Yck, which is anchored to the plasma membrane.

### Coordinate dynamics of the SPS-sensor complex

Except for the *SSY5*-E131K mutation, all gain-of-function *SSY5* and *PTR3* mutants analyzed for *EC*_50_ turned out to be both constitutive and hyper-responsive. This is consistent with the interpretation by Poulsen et al. (2005b, 2006, 2008) that these mutations impact global conformations of an intimately connected SPS complex and thereby influence early conformational changes of Ssy1, i.e. changes prior to phosphorylation of Ptr3 and Ssy5. In the framework of Fig. 2, this possibility would mean that not only the 7 *SSY1* mutants discussed in detail above, but also *PTR3*-T435K, *PTR3*-Q439R, *SSY5*-E512K, *SSY5*-F575V, and *SSY5*-Q576P change the equilibrium constant between states B and D of Ssy1 (reactions E2 and E3) as well as the equilibrium constant between states A and F (reactions E4 and E5), so that both of the signaling states D and F are favored. The existence of the constitutive, non-hyper-responsive mutant *SSY5*-E131K strengthens this interpretation, since the weakening of the LxxVxE motif for binding to PP2A by substitution of a negative charge with a positive charge fully explains the constitutive phenotype. In this connection it should be noted that the mutation affects a disordered part of Ssy5, not expected to strongly affect conformations of Ssy1. We see no other simple explanation of the lowered *EC*_50_ of the other mutants than an influence on the conformation of a tight, dynamic complex of Ssy1, Ptr3, and Ssy5. Our interpretation is also in accordance with the observation that the substituted side chains of the 5 mutants are surface exposed in the AlphaFold models of Ptr3 (AF-P43606; Fig. 5) and Ssy5 (P47002; Supplementary Fig. 15b). As described above, the AlphaFold 3 analysis of PP2A and Ssy5 together with the hyper-responsiveness of *SSY5*-F575V and *SSY5*-Q576P suggests inclusion of the 3 subunits of PP2A into this complex.

### Summary of early events in SPS signaling

As pointed out throughout this paper, we regard equilibria between conformational states of the SPS complex to be central for an understanding of the signaling process. In addition, it is useful to consider likely steps in signaling; they may be summarized as follows:

#### Presignaling

When the growth medium does not contain eliciting amino acids, we envisage that a large part, perhaps the majority, of the SPS complexes have Ssy1 in an outward-open conformation (state A, Fig. 2). No or little Ssy1 should be in states B, C, and D, and equilibrium constants should ensure that the amount of complexes with Ssy1 in state F is low, corresponding to the low wild-type basal level of signaling.

#### Step 1

Binding of an amino acid molecule into the ligand binding pocket of Ssy1 promotes changes of positions of TM1a, TM6b, and the C-terminal latch, leading to an inward-facing occluded conformation of Ssy1.

#### Step 2

Ptr3 is partially released from Ssy1.

#### Step 3

Phosphorylation of one or more of S514, T517, and S521 of Ptr3 further releases Ptr3 from Ssy1.

#### Step 4

This release allows homodimerization of Ptr3 and leads to a situation allowing phosphorylation of the Ssy5 prodomain.

#### Step 5

The Ssy5 prodomain is phosphorylated, ubiquitinated, and degraded. The released Ssy5 catalytic subunit processes the transcription factors Stp1 and Stp2, thus furthering the signal to the transcriptional apparatus.

Some or all phosphorylations in steps 3 and 5 are counteracted by dephosphorylations by PP2A^Rts1^, an activity that is needed to keep Stp1 processing low when amino acids are absent from the exterior.

### Main hypotheses and suggestions for experiments

Results presented here have been obtained by computational modeling, and it will be of obvious interest to test many of the suggestions and possibilities by wet-lab experiments and further computational approaches. Some of the most important hypotheses and suggestions put forward here are:

#### The signaling conformation of Ssy1 is inward-facing occluded

Our analysis of 7 gain-of-function Ssy1 mutations suggests that an inward-facing occluded conformation is signaling, or more precisely, a conformation close to that of the templates 5OQT, 6F34, and 3GIA (legend to Fig. 2) is signaling. This represents a hypothesis that should be tested and, if validated, hopefully refined. We have considered the alternative hypothesis that another occluded conformation triggers signaling, like that of states C and E (“occluded”) but find it less likely since it does not fit the relationship between phenotype and modeled structure of the 7 mutants. A simple example is the mutant F333S, in which a pi–pi interaction has been lost. This loss is from the Phyre2 models predicted to promote part of the structural change associated with inward opening. Since the mutation causes hyper-responsiveness and increased basal level of signaling, the modeling suggests that the signaling conformation is not fully inward closed, i.e. not “occluded” (states C and E) but rather “inward-facing occluded” (states D and F). It is conceivable that this could be further analyzed by cryo-electron microscopy (cryo-EM) studies using the constitutive Ssy1 mutants, with or without its N-terminal domain. Isolation of large quantities of high-quality Ssy1 can be carried out as described (Scharff-Poulsen and Pedersen 2013). Selected amino acid analogs from the collections of van Zeebroeck et al. (2009) and Lee et al. (2025) may be applied to obtain Ssy1 in various conformations. Inhibitors of SPS signaling may also be used to obtain mechanistic suggestions from the kinetics of inhibition. A better prediction of Ssy1 conformations might also be obtainable by a recent coevolution-powered machine learning approach used for glucose transporters (Mitrovic et al. 2023).

#### Role of Ptr3 phosphorylation in signaling

Understanding the specific sites and levels of phosphorylation of Ptr3 is key to substantiate our hypothesis that phosphorylation of Ssy1-bound Ptr3 results in rotational, partial splitting of Ptr3 from Ssy1, making Ptr3 accessible to Yck. This may be achieved by site-directed mutagenesis of phosphosites followed by sodium dodecyl sulfate polyacrylamide gel electrophoresis procedures. Protein mass spectrometry analysis, for example, as described by Li et al. (2024) is also important. Effects of Ptr3 phosphorylation may also be approached using molecular dynamics simulations.

#### The proposed structure of the Ssy1–Ptr3–Ssy5 complex

Cryo-EM of in vitro prepared complexes of, eg Ptr3, Ssy5, and the N-terminal domain of Ssy1 may be possible using a procedure successfully applied for the APC family amino acid transporter SLC38A9 in complex with RagA, RagC, and Ragulator (Fromm et al. 2020). Likewise, detailed 2-hybrid experiments with small, selected parts of the 3 subunits, following the strategy of Omnus and Ljungdahl (2013) for the Ptr3–Ssy5 interaction, could well be informative.

## Supplementary Material

jkaf312_Supplementary_Data

## Data Availability

The authors affirm that all data necessary for confirming the conclusions of the article are present within the article, figures, and Supplementary Figures and Tables. Protein sequences from the UniProt database were used in alignments as described above. Protein structures from the Protein Data Bank were used for modeling as described above. Supplemental material available at G3 online.
