# Supplementary material for: Structural bases of signal generation and transduction by the SPS amino acid sensor of Saccharomyces cerevisiae
Source: G3 (Bethesda). 2025 Dec 24;16(3):jkaf312. doi: 10.1093/g3journal/jkaf312 (PMC12958826; doi:10.1093/g3journal/jkaf312)

Fig. S1: Alignment of the *S. cerevisiae* amino acid receptor Ssy1 with *S. cerevisiae* YAT family amino acid transporters

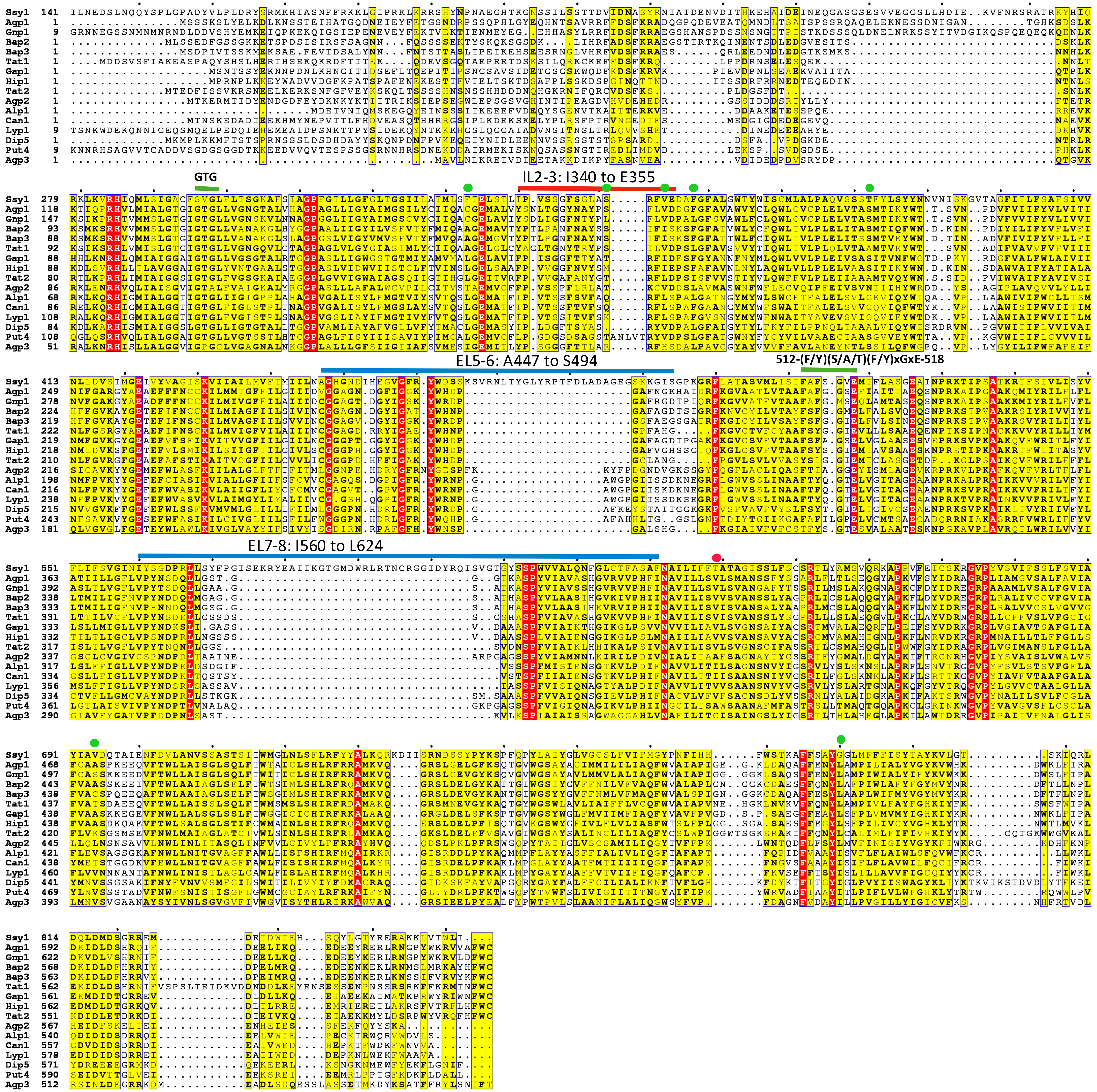

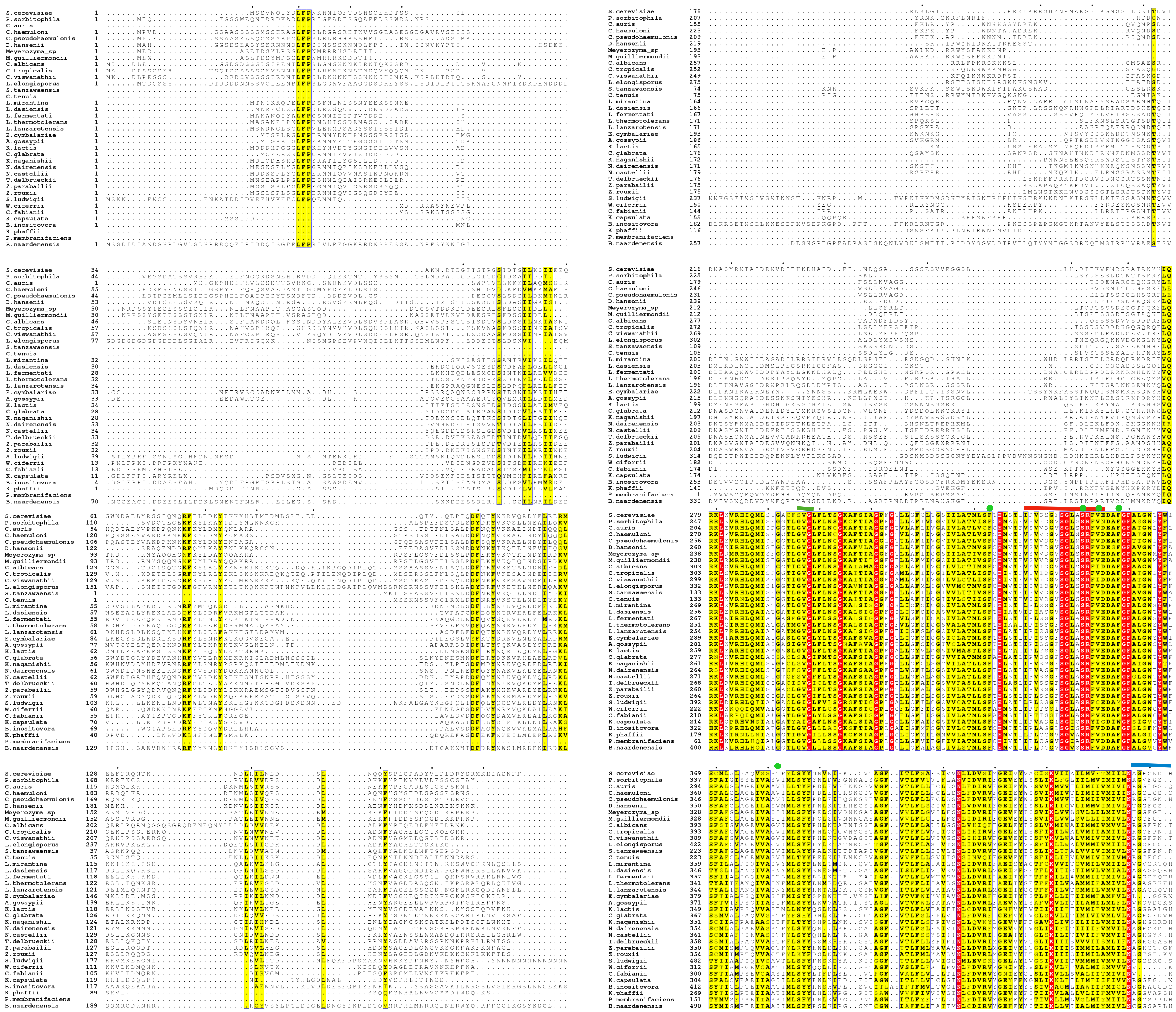

Fig. S2. Alignment of 37 fungal Ssy1 orthologs

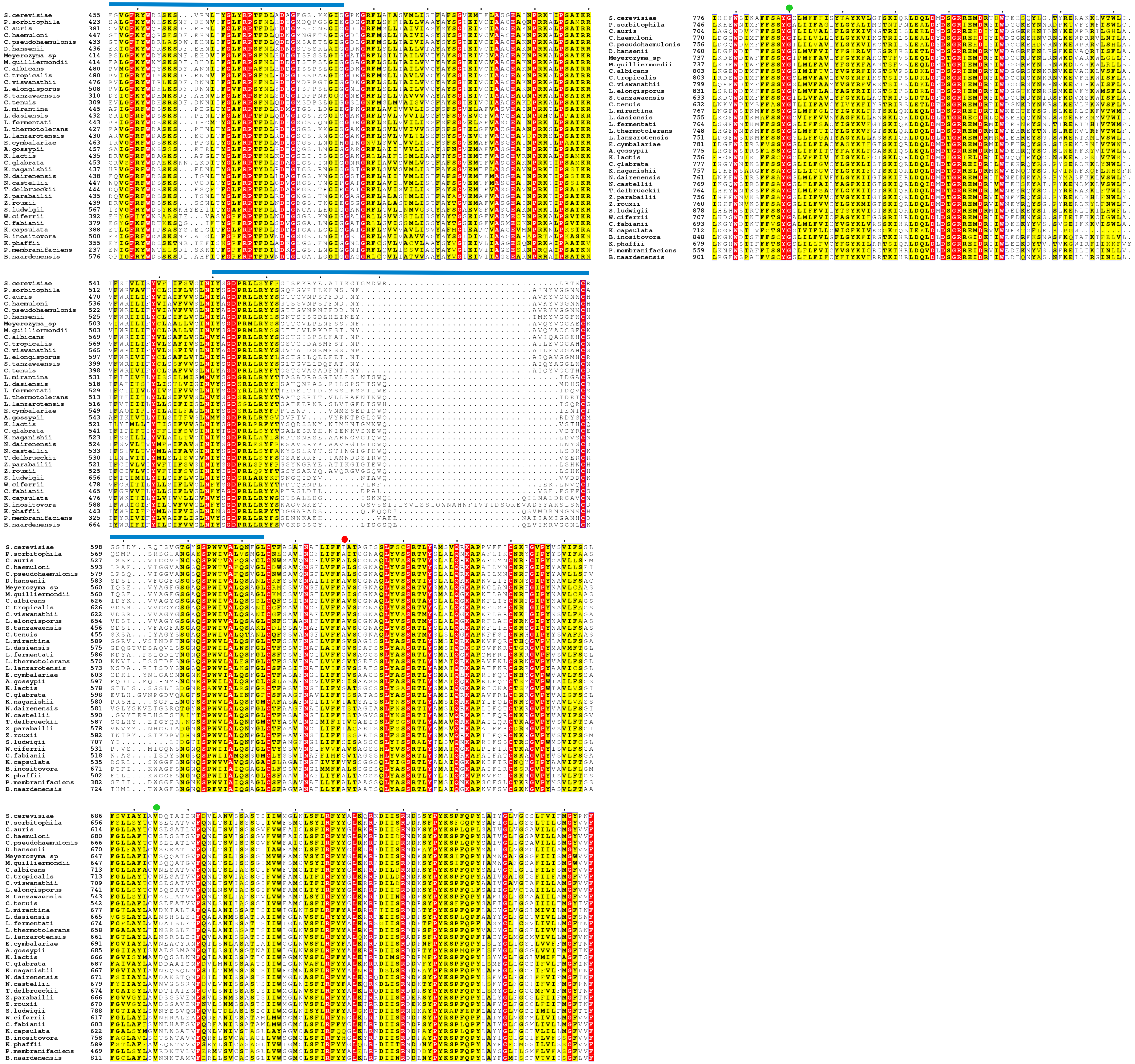

Fig. S2 continued. Alignment of 37 fungal Ssy1 orthologs

Fig. S3: Positions of residues fully conserved in YAT transporters and Ssy1.

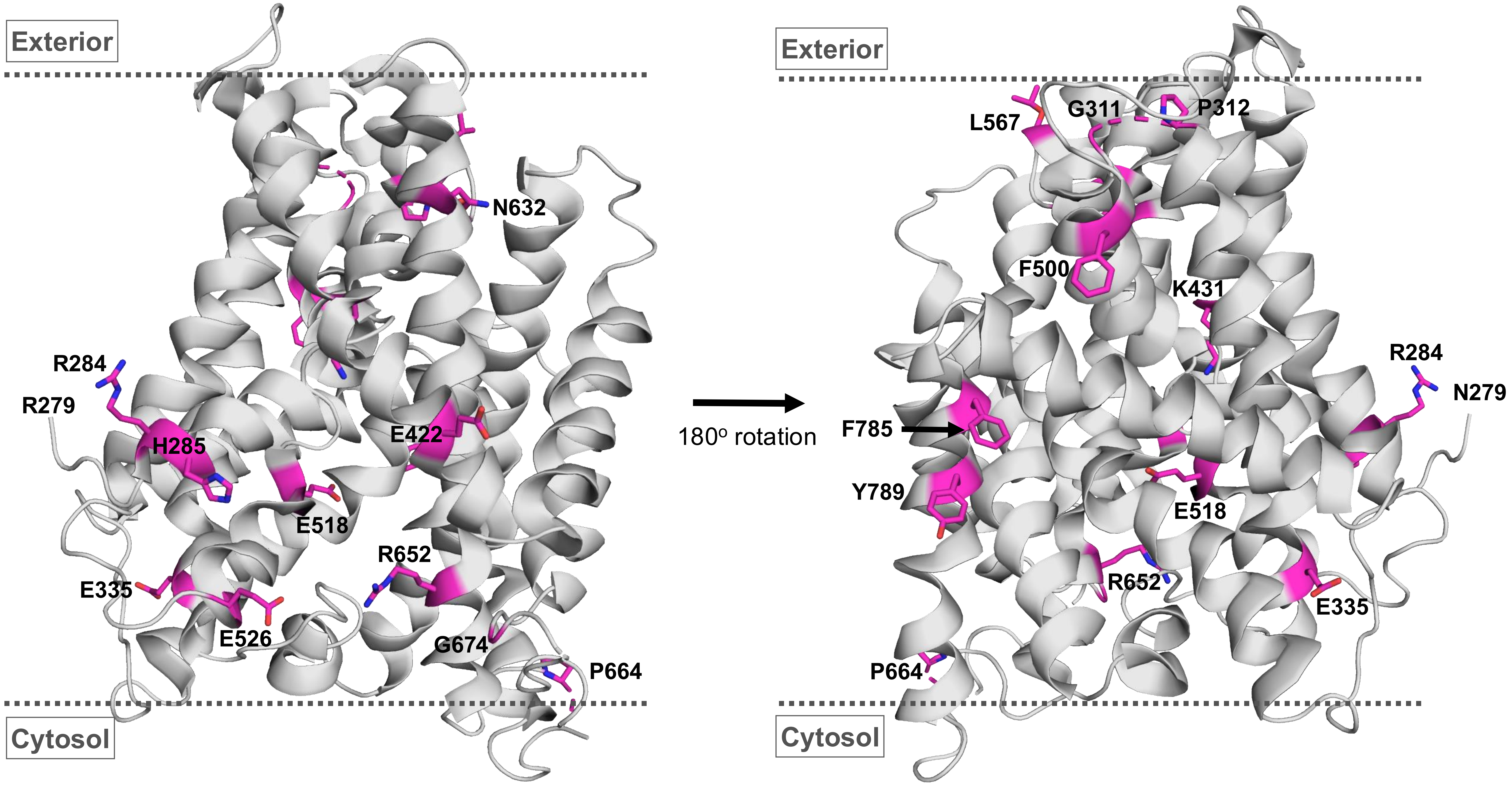

Residues conserved in YATs (Ssy1 numbering)

Residue	Face	TM/loop
R284	Intracellular	TM1a
H285	Intracellular	TM1a
G311	Extracellular	EL1-2
P312	Extracellular	TM2
E335	Intracellular	TM2
E422	Intracellular	TM5
K431	Transport path	TM5
G448[a)]	Extracellular	EL5-6
G458[a)]	Extracellular	EL5-6
Y461[a)]	Extracellular	EL5-6
F500	Extracellular	EL5-6
E518	Transport path	TM6b
E526	Intracellular	IL6-7
L567	Extracellular	EL7-8
S613	Extracellular	EL7-8
P614	Extracellular	EL7-8
N632	Extracellular	TM8
R652	Intracellular	TM8
P664	Intracellular	IL8-9
G674	Intracellular	IL8-9
A730[a)]	Intracellular	IL10-11
F785	Extracellular ?	TM12
Y789	Extracellular ?	TM12

Fig. S4: Displacement of V694 during the shift from an outward open to an occluded conformation

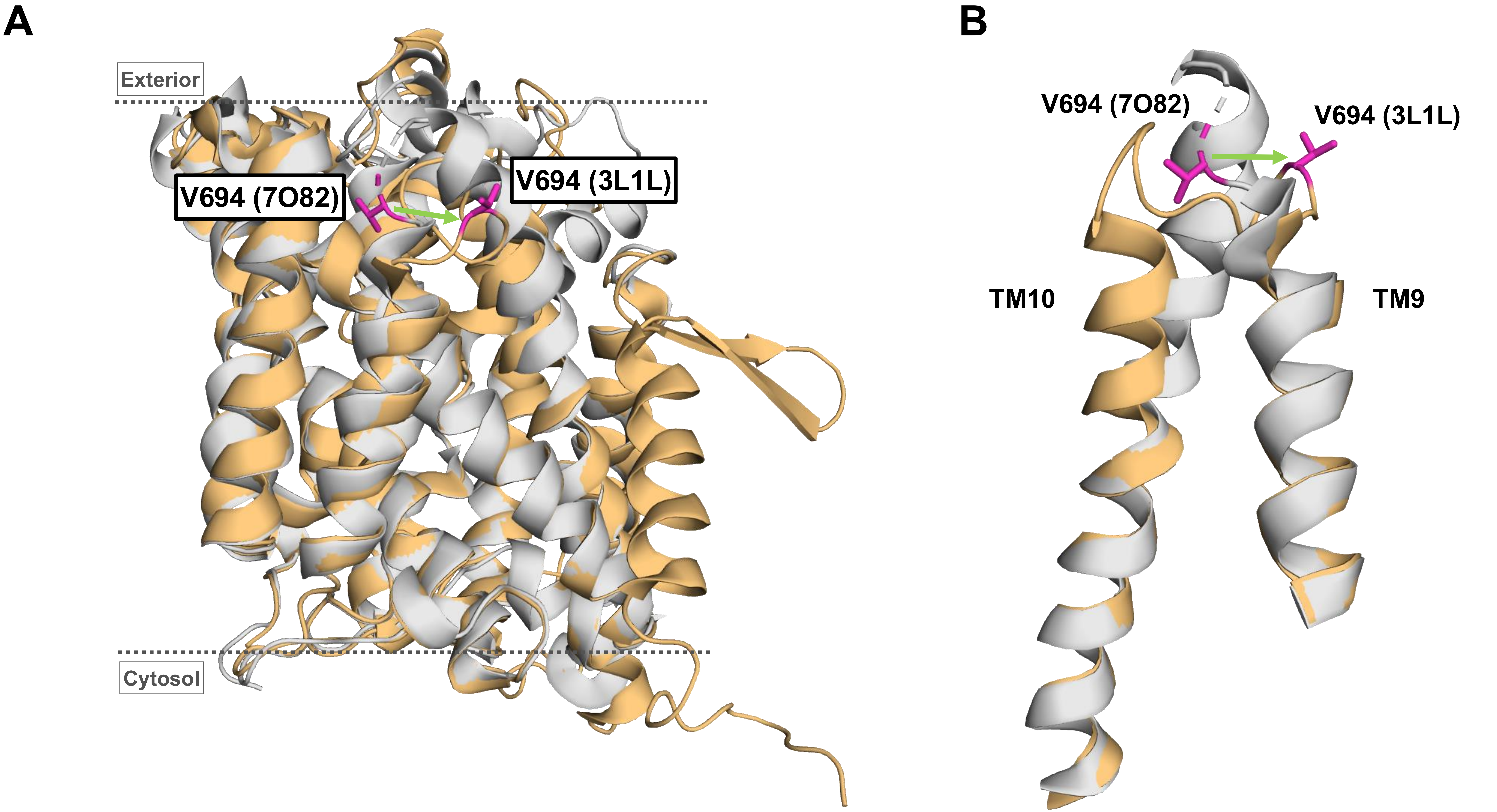

Fig. S5. Modeling of extracellular loops suggests a cap that can close the extracellular gate.

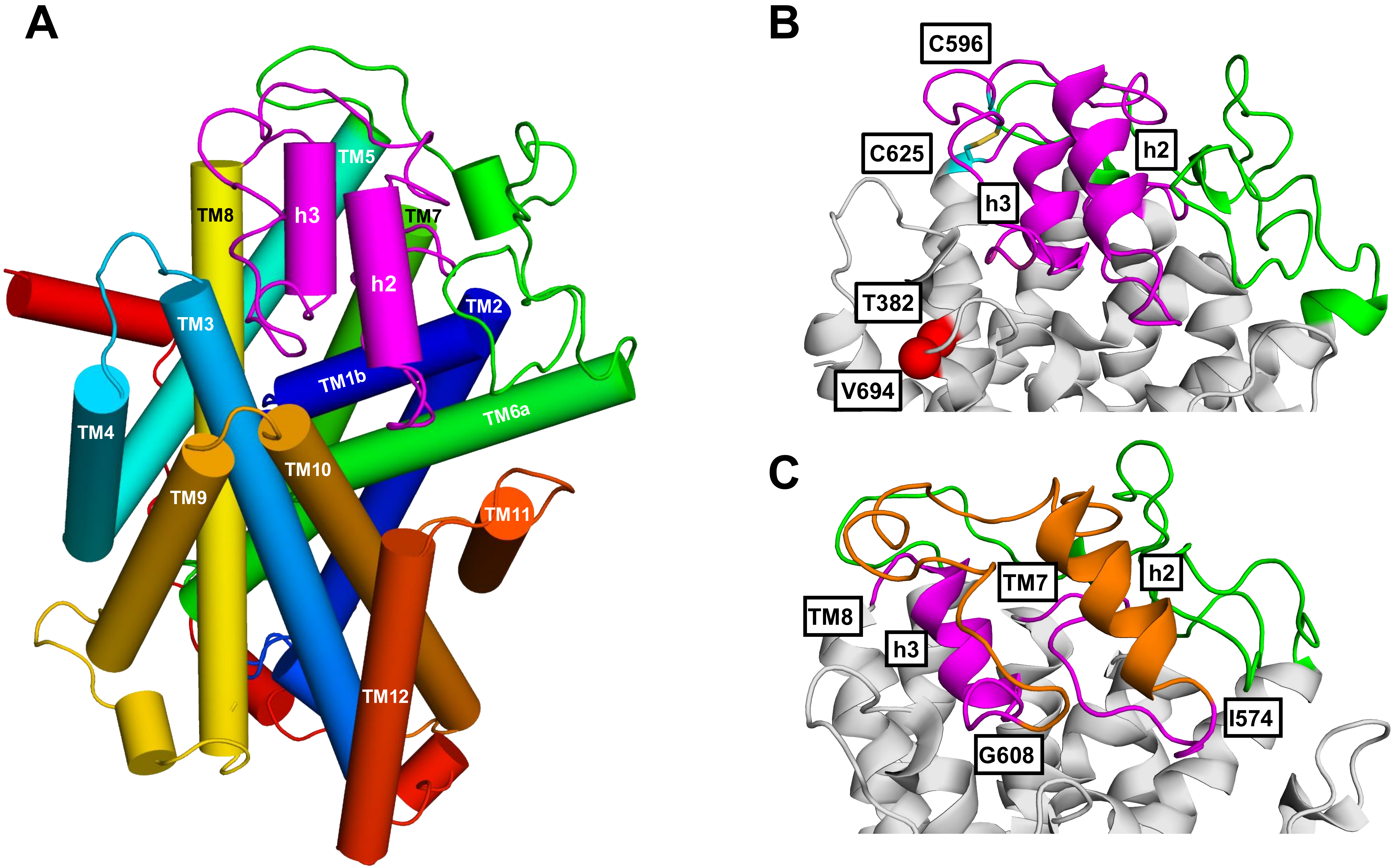

Fig. S6. Pi-pi interactions between Ssy1 F333 and F521 favor a closed intracellular gate

A

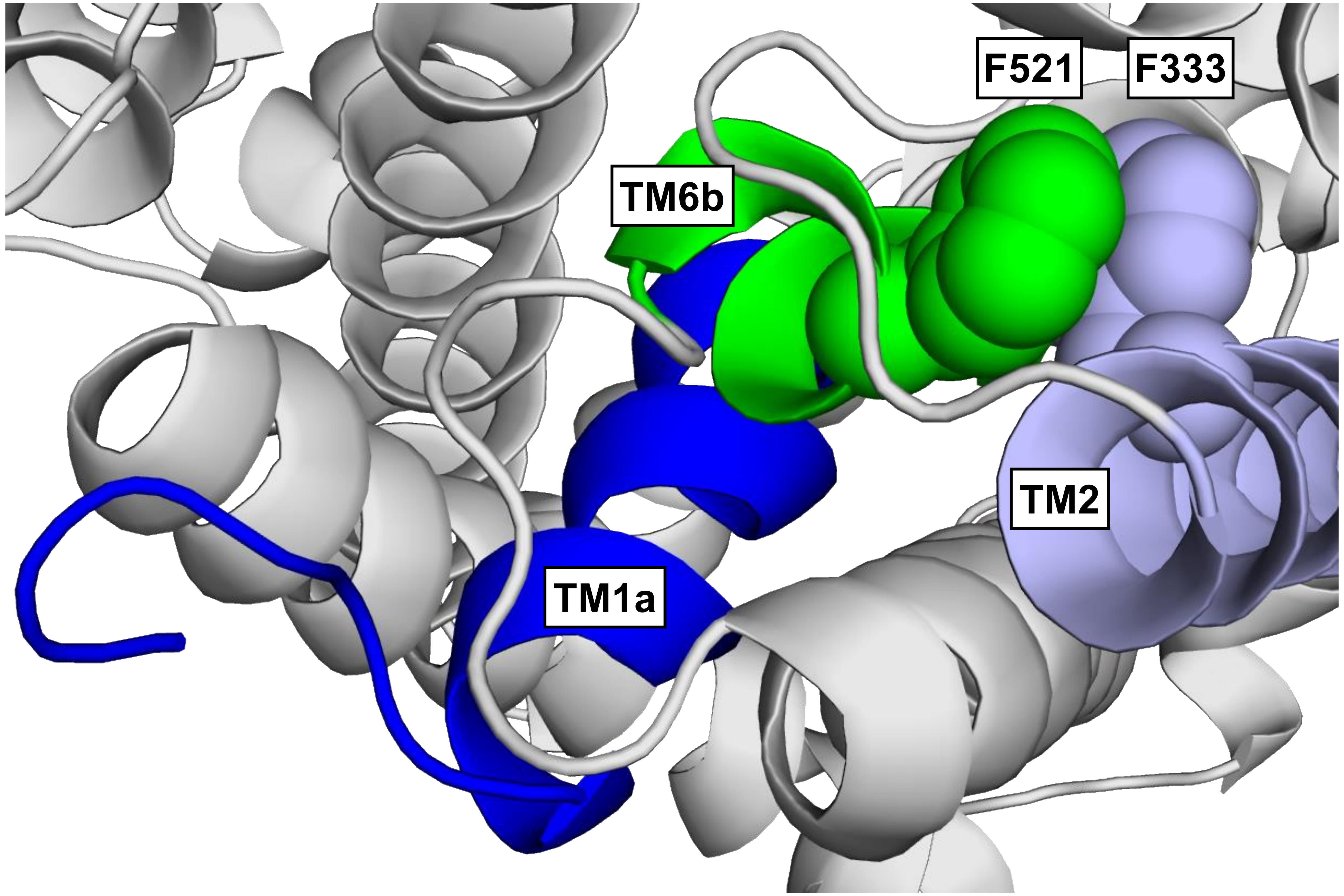

B

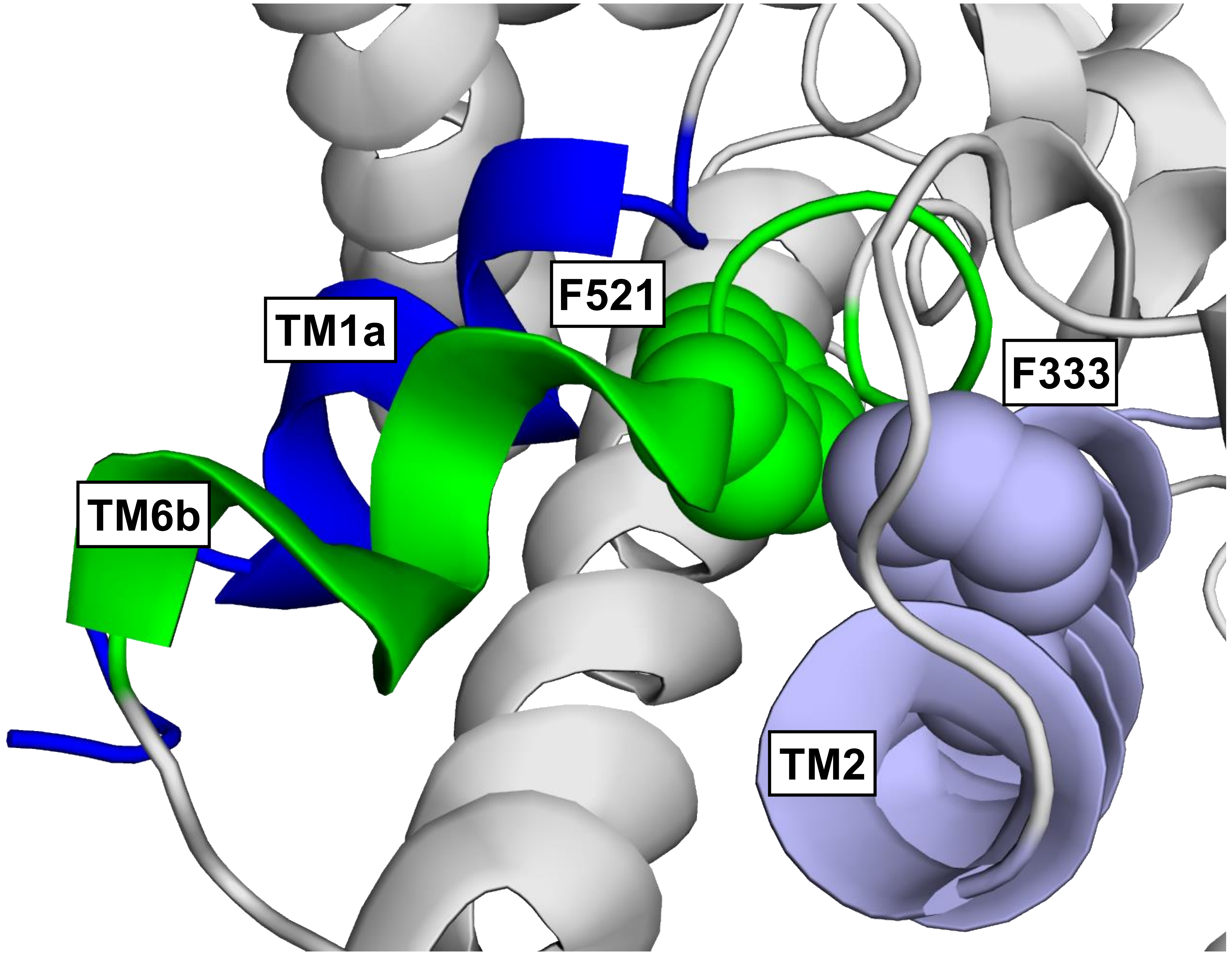

Fig. S7. A hydrophobic cluster at the bottom of the binding pocket of Ssy1 closes the intracellular gate

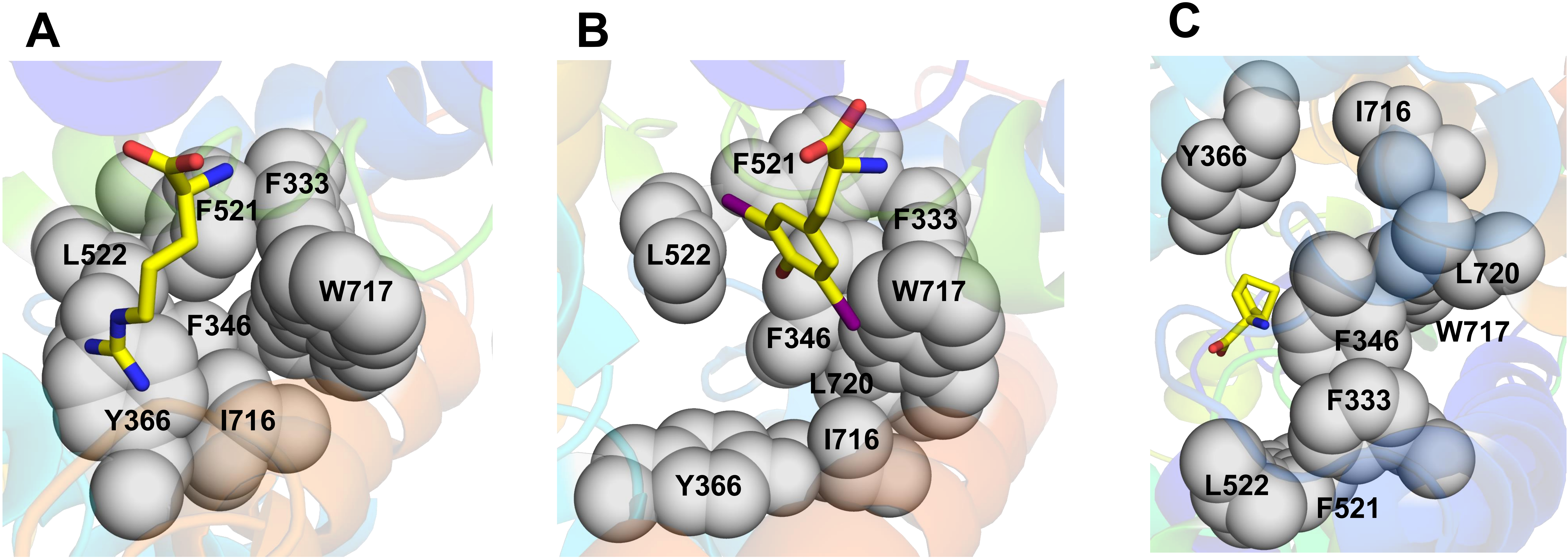

Fig. S8: Alignment of the Ssy1 transmembrane core of 18 *Saccharomycetaceae* orthologs

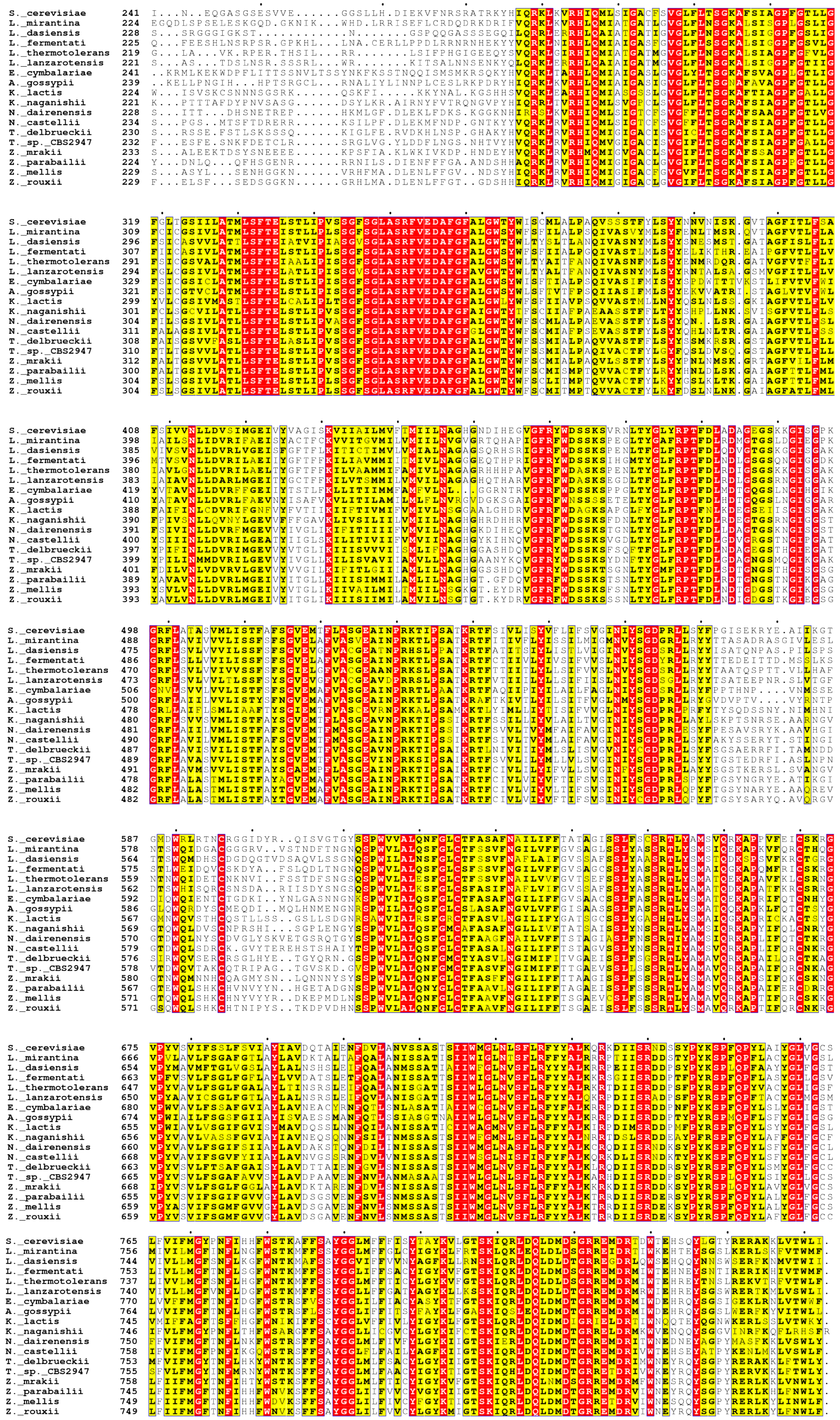

Fig. S9. Binding of leucine in an Ssy1 model based on a LAT2 structure

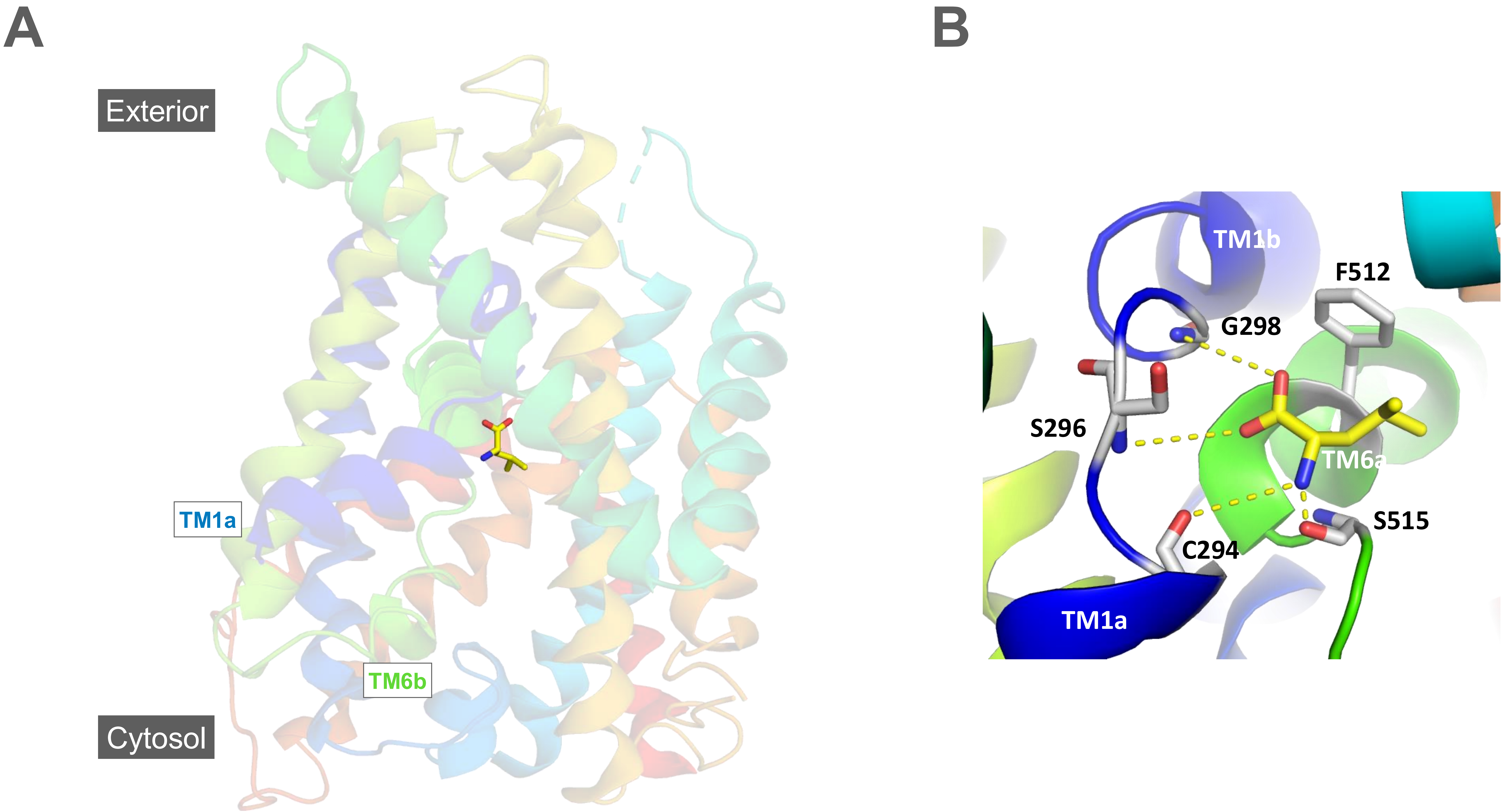

Fig. S10. The hypo-responsive mutation T639I affects ligand binding and conformational shifts of the TM1 loop

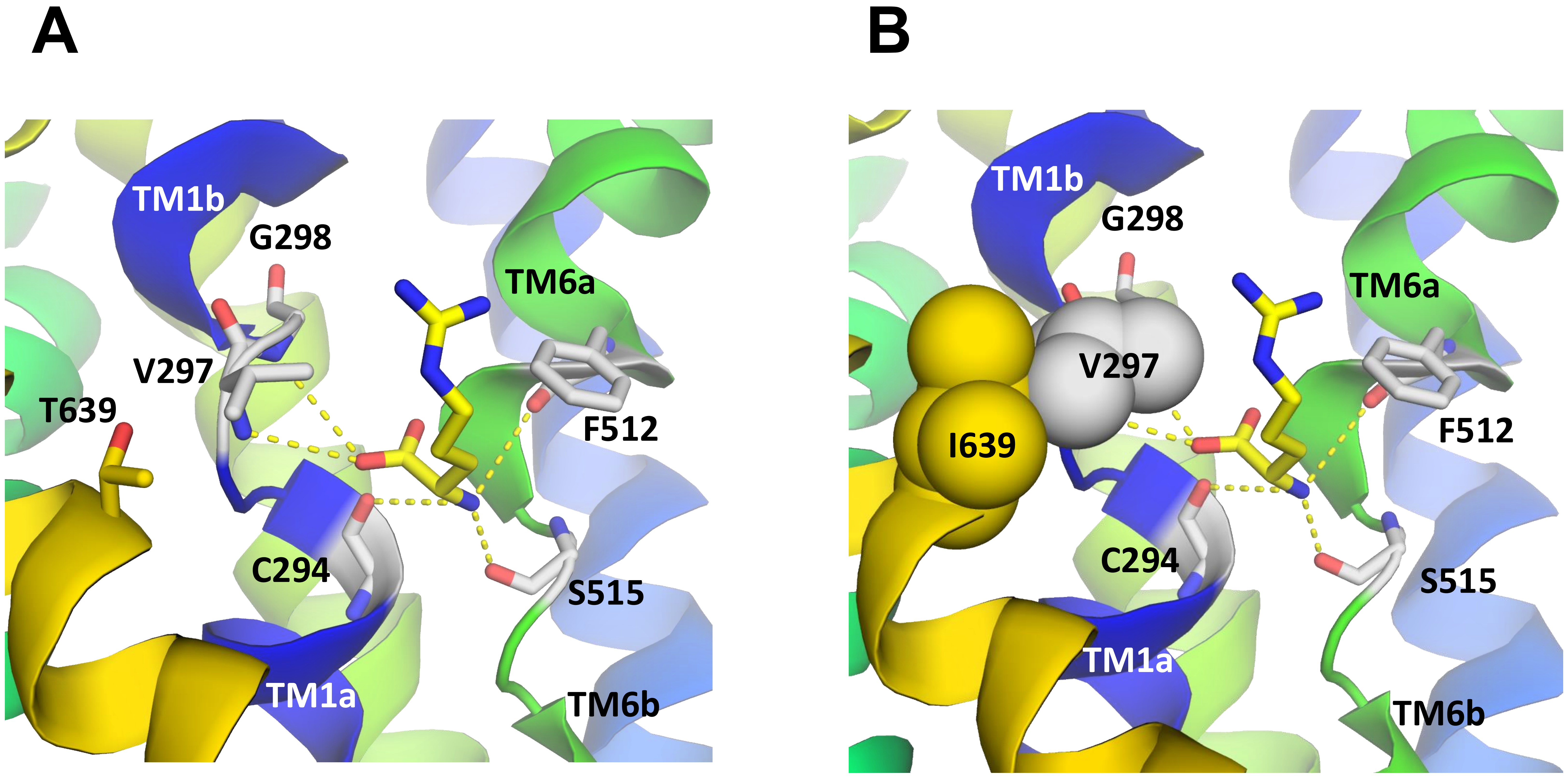

Fig. S11. Prediction of disorder and structure of Ptr3

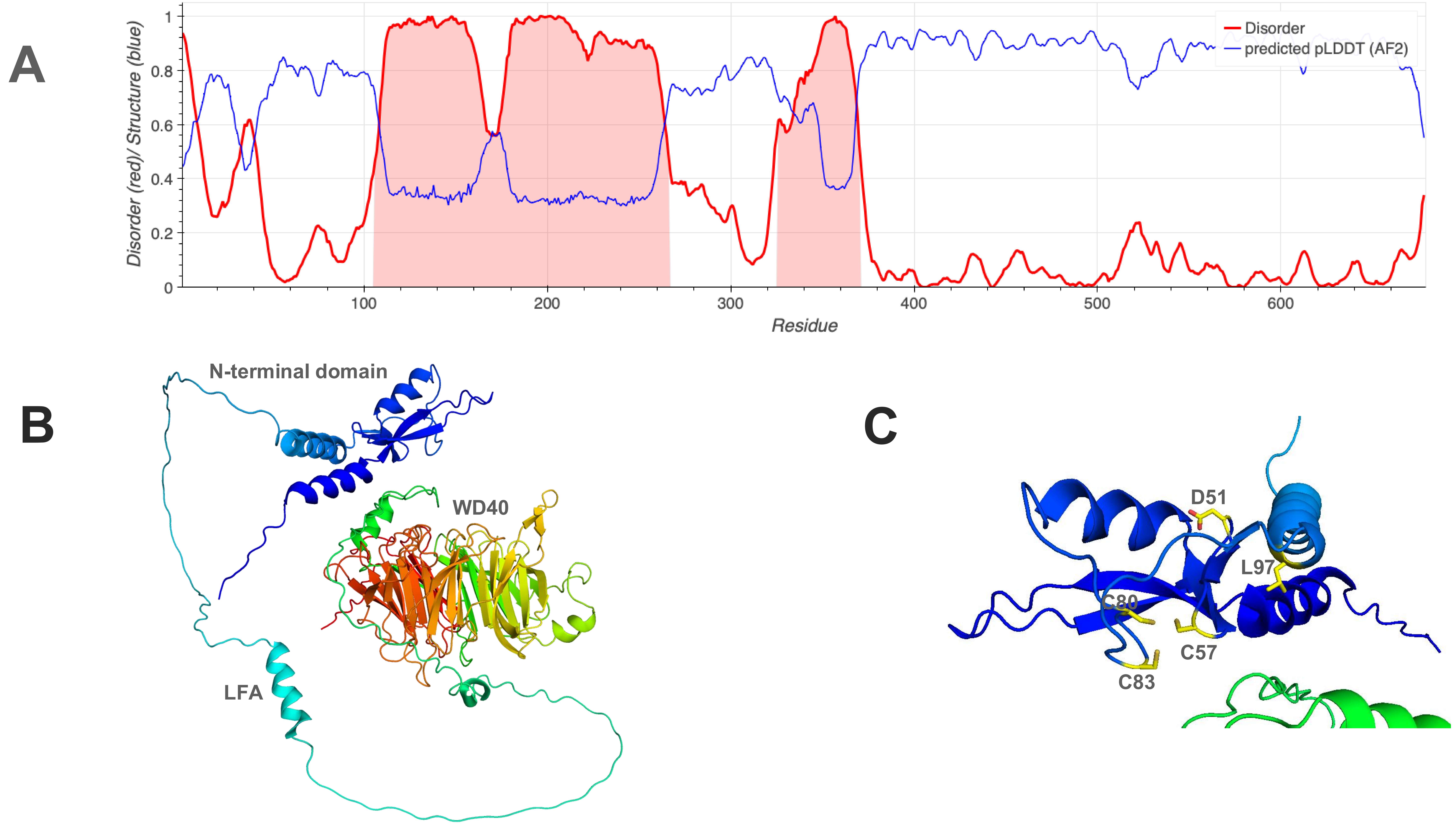

Fig. S12. Alignment of N-terminal regions of 32 Ptr3 orthologs

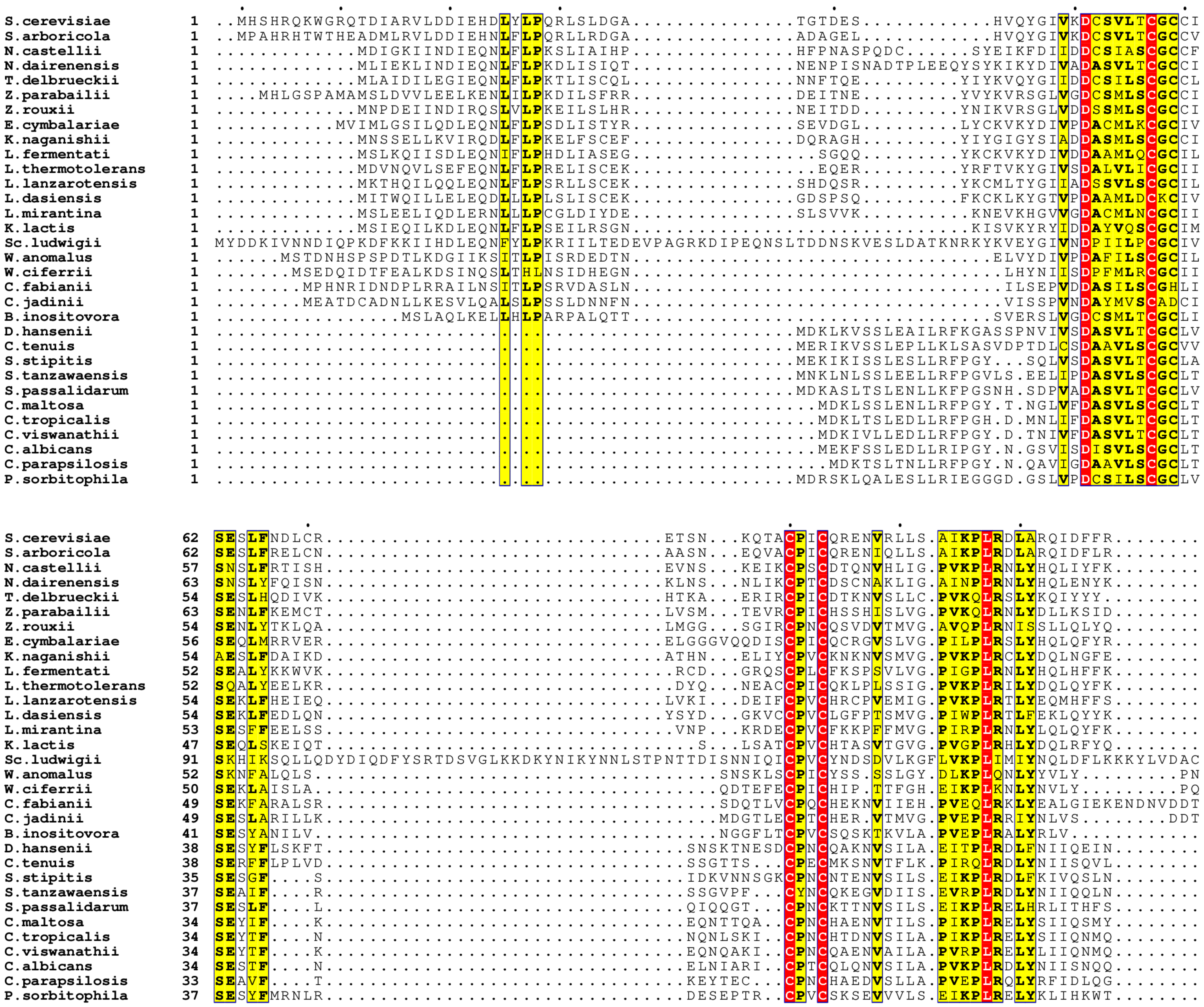

Fig. S13. Sequence alignment of Ptr3 from 46 related fungal species.

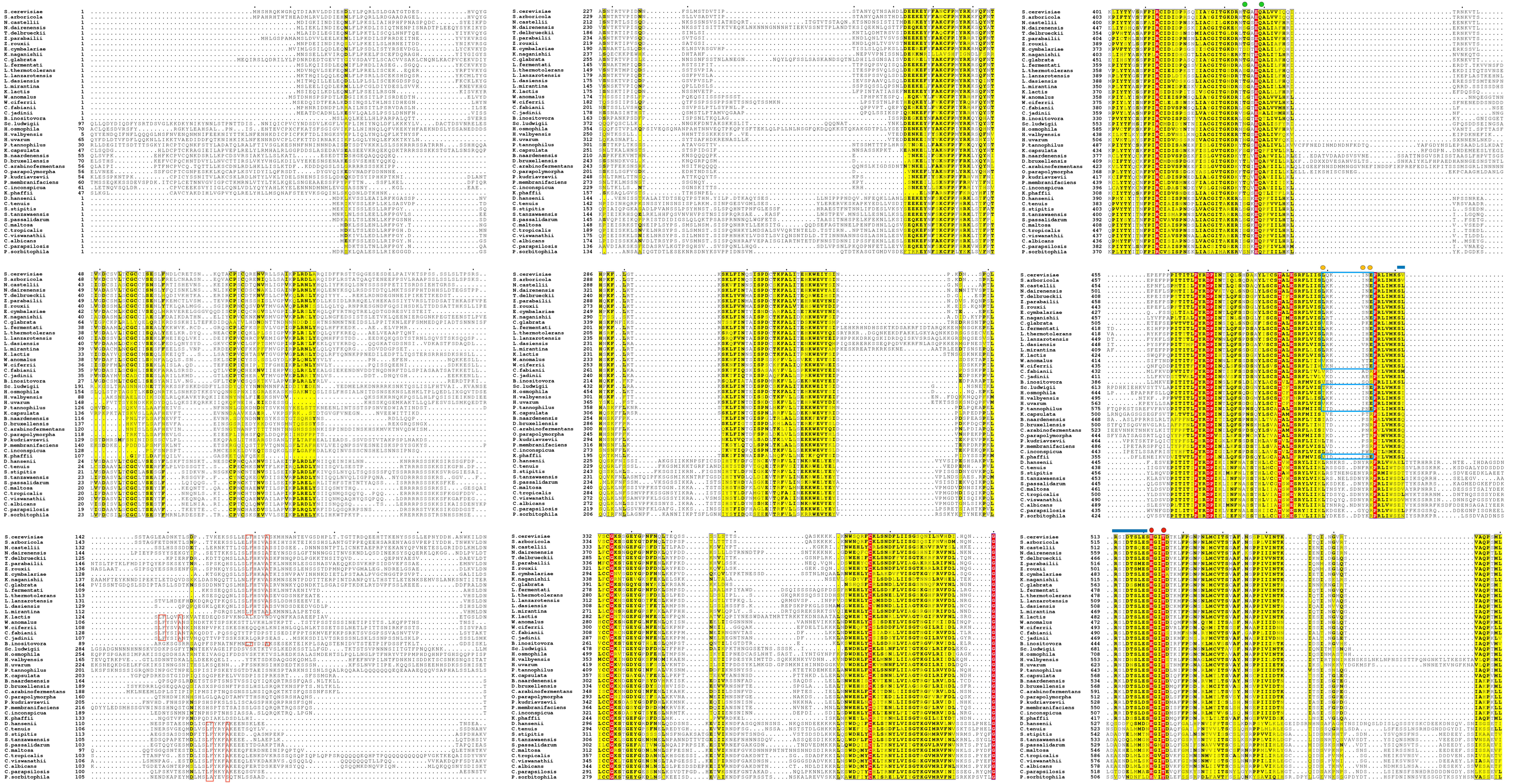

Fig. S13 continued.

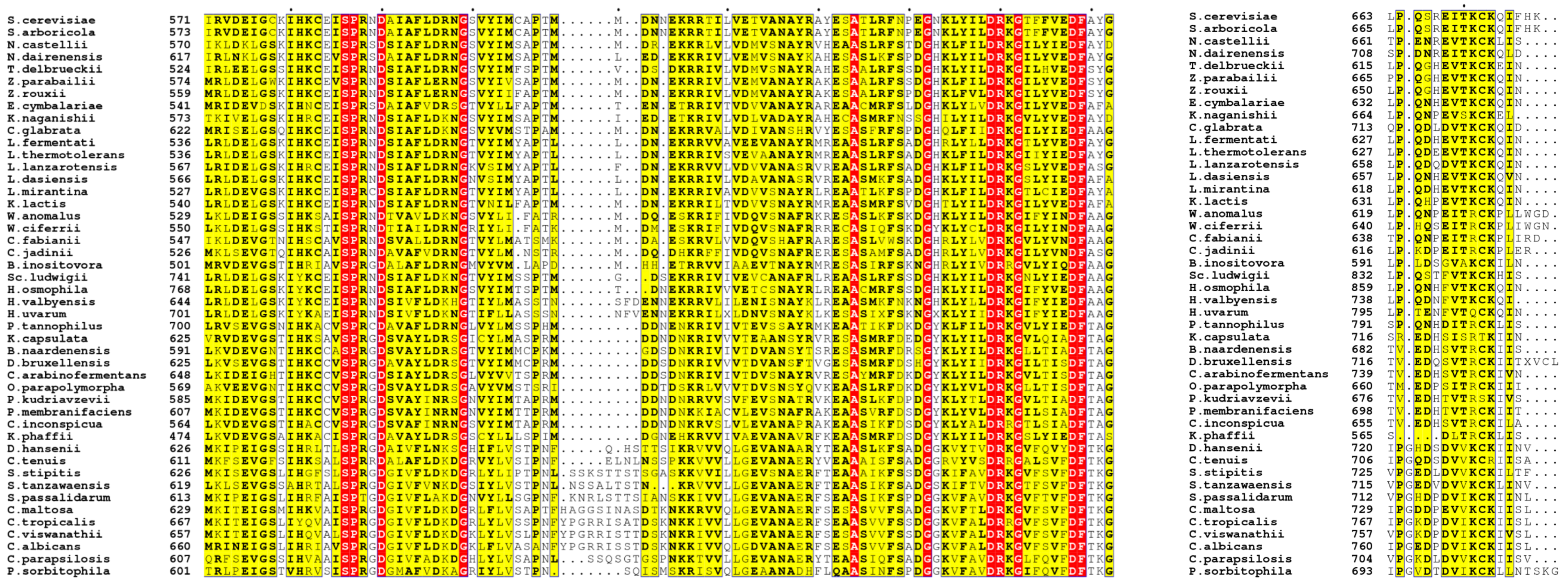

Fig. S14. Structure of *S. cerevisiae* Yck1 and its interaction with the N-terminal of Ssy1

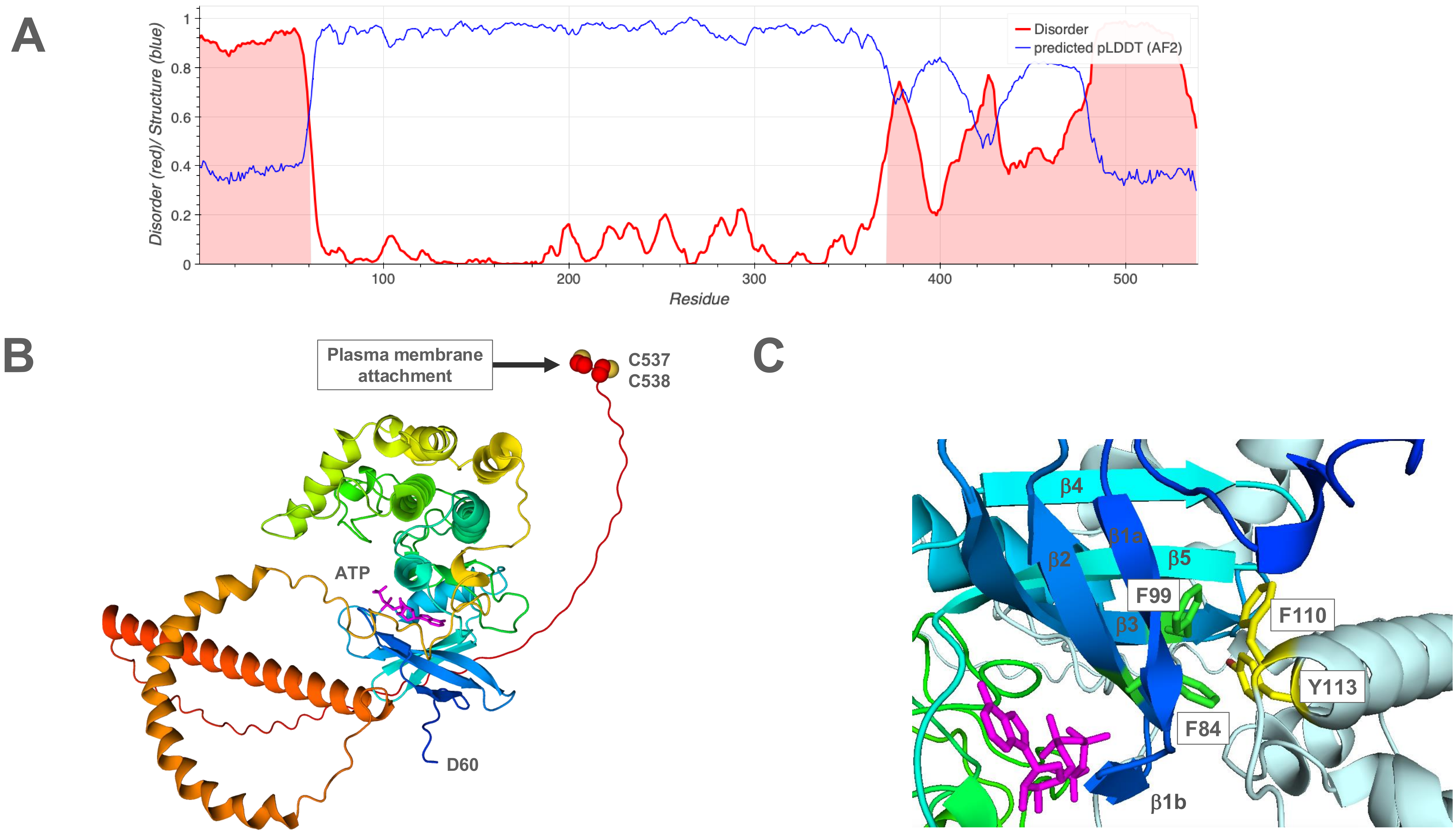

Fig. S15. Prediction of disorder and structure of Ssy5

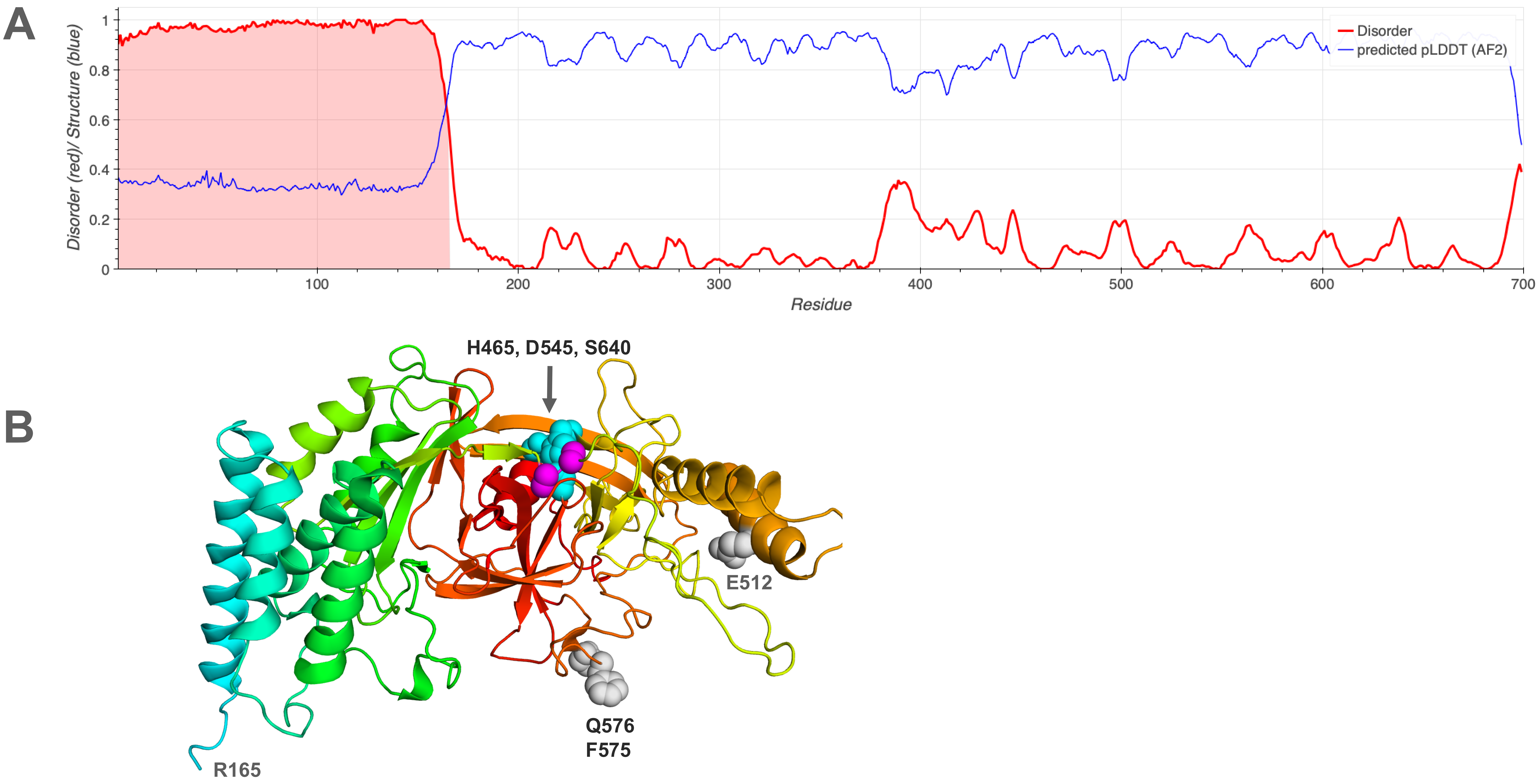

Fig. S16. Alignment of 18 Ssy5 orthologs highlights functional motifs

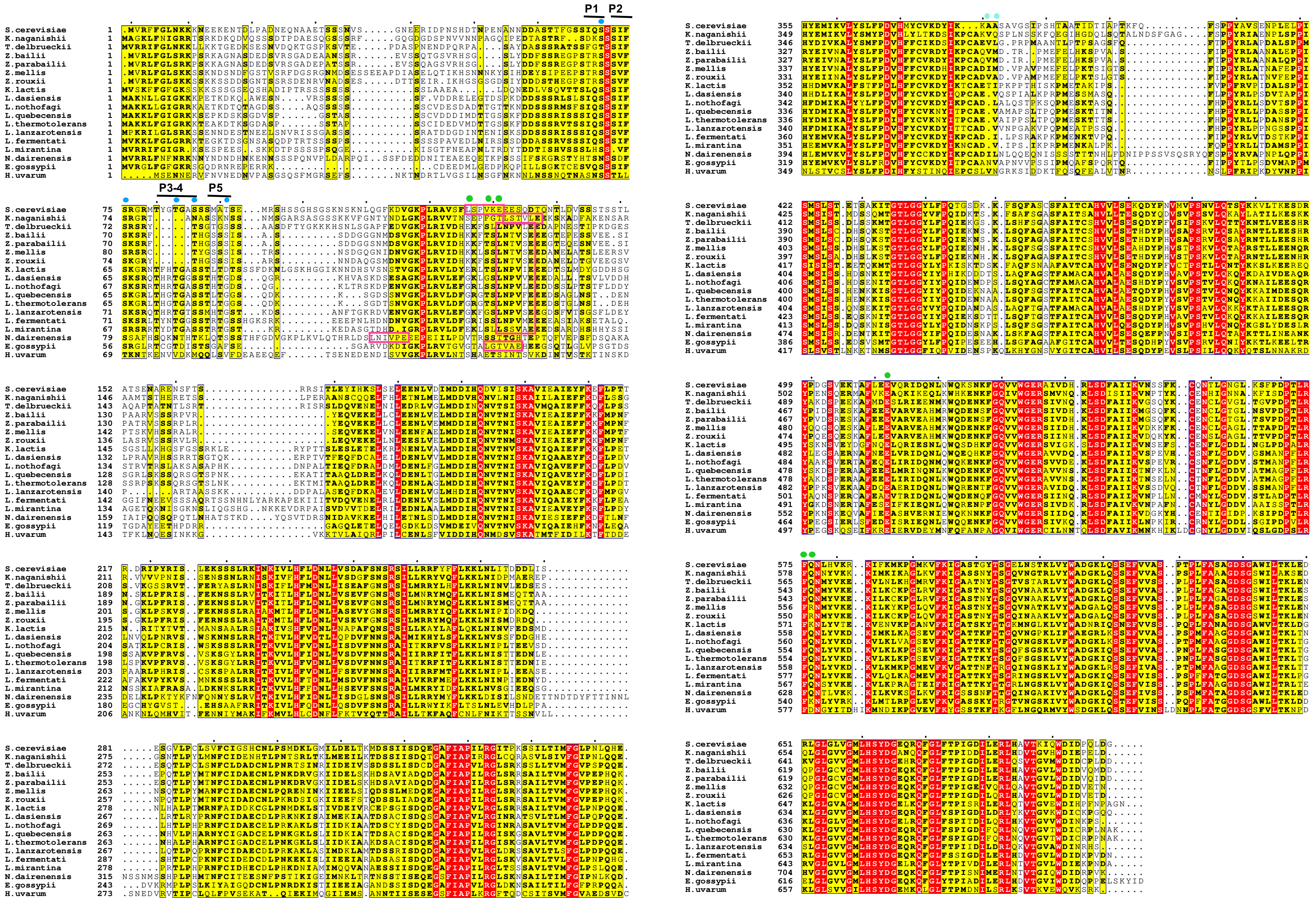

Fig. S17 Interactions between Ssy5 and PP2A predicted by AlphaFold 3

A

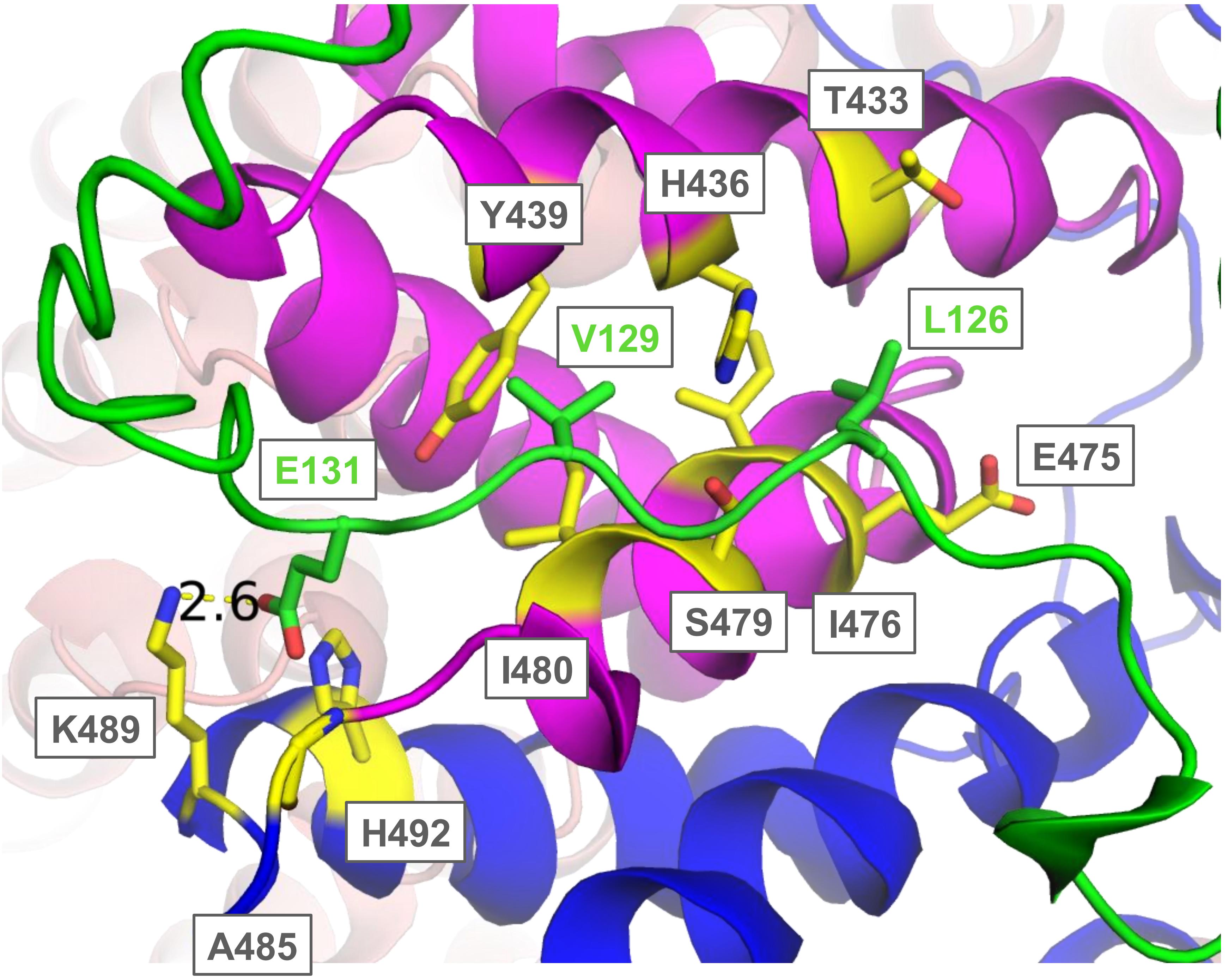

B

LxxIxE motif		Interactor	
**Ssy5**	**BubR1** [a]	**B56γ** [a]	**Rts1**
**L126**	L669		
		S173	S422
		T184	T433
		H187	H436
		E226	E475
		I227	I476
**V129**	I672		
		H187	H436
		Y190	Y439
		S230	S479
		I231	I480
**E131**	E674	K240	K489
		H243	H492
		A236	A485

[a] (Wang et al 2016)

C

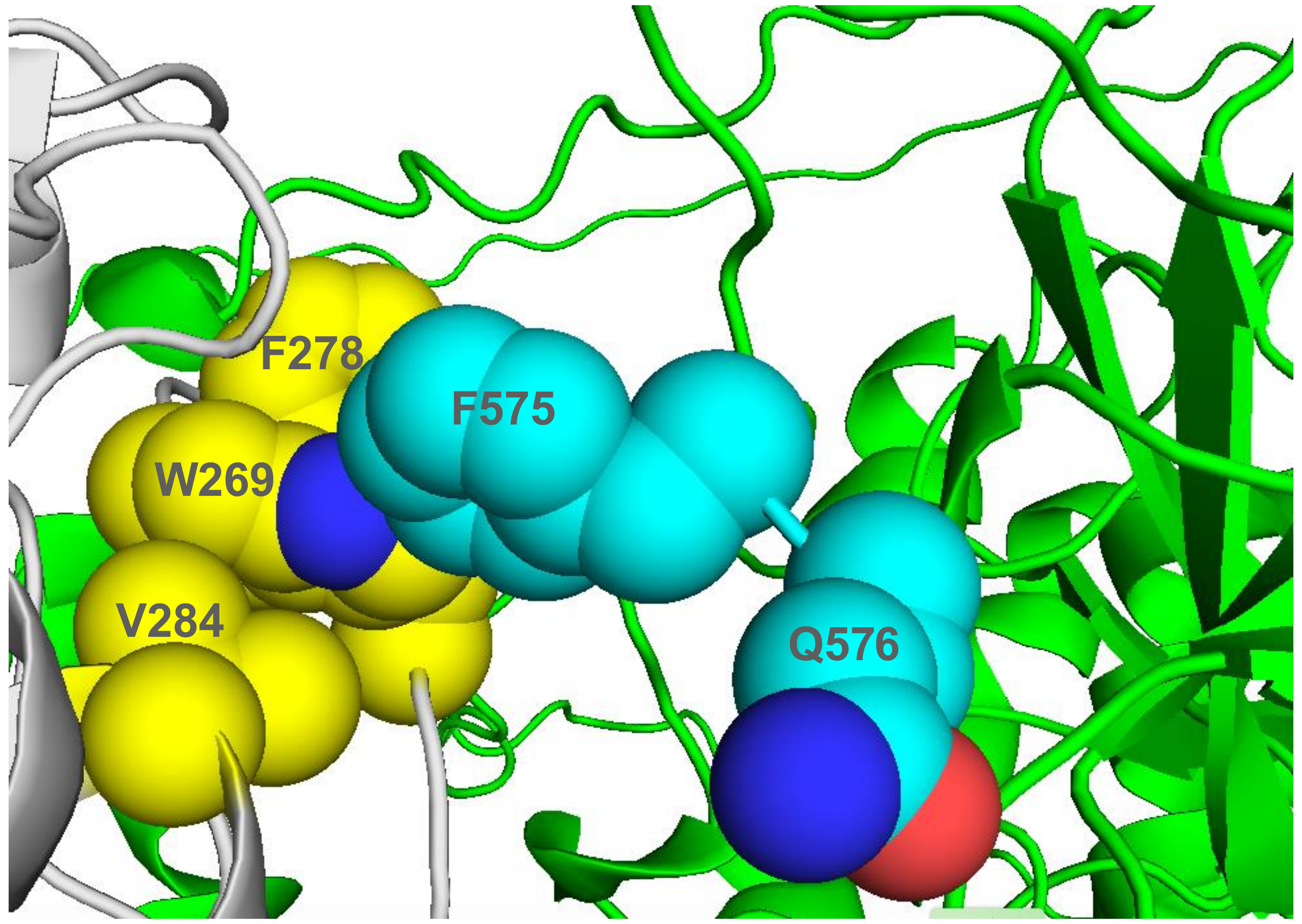

Fig. S18. Binding of the Ssy5 phosphosites region to the catalytic cleft of Pph21

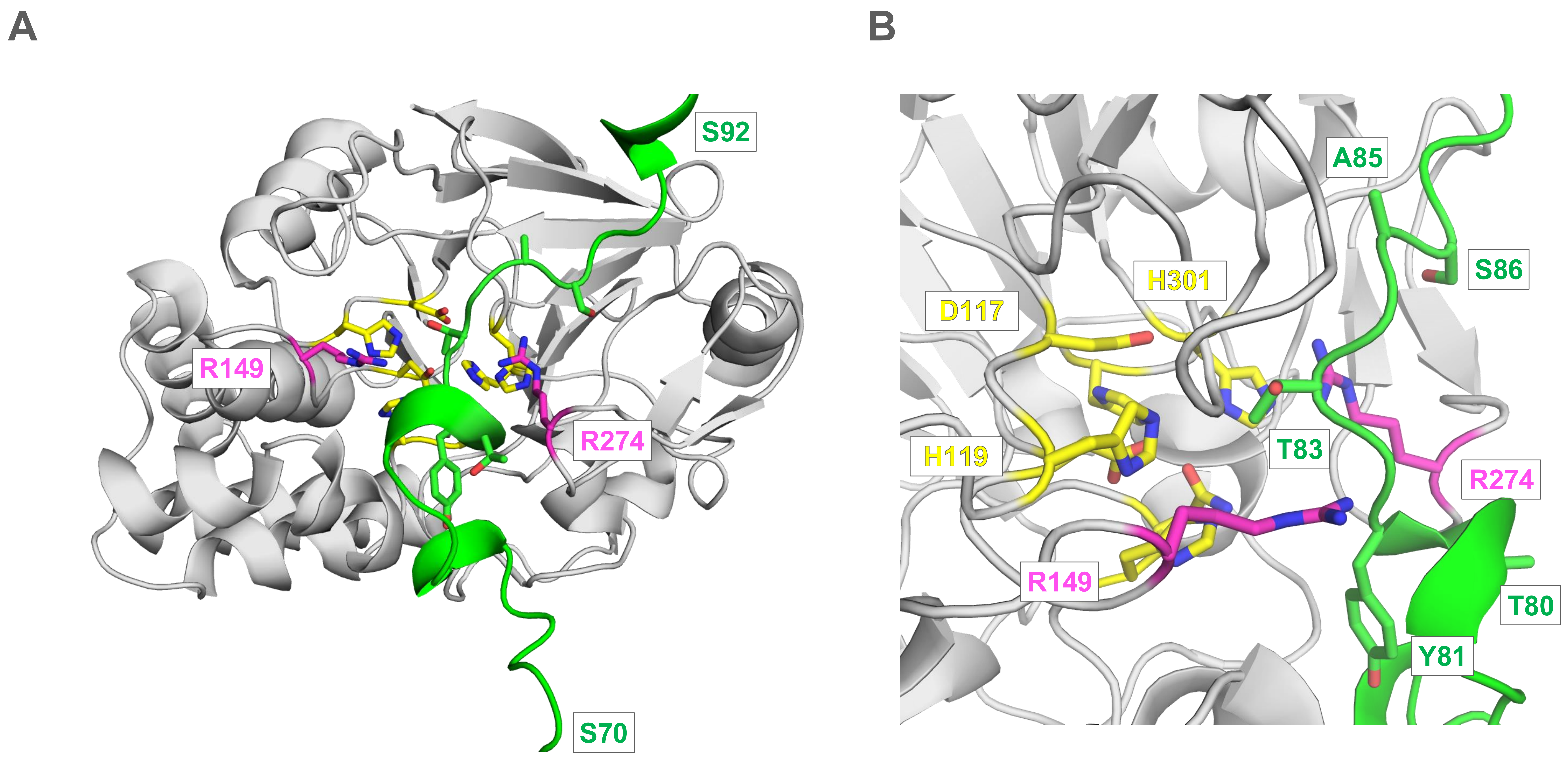

Fig. S19. AlphaFold 3 model of Ssy5-PP2A complex showing pIDDT confidence estimates

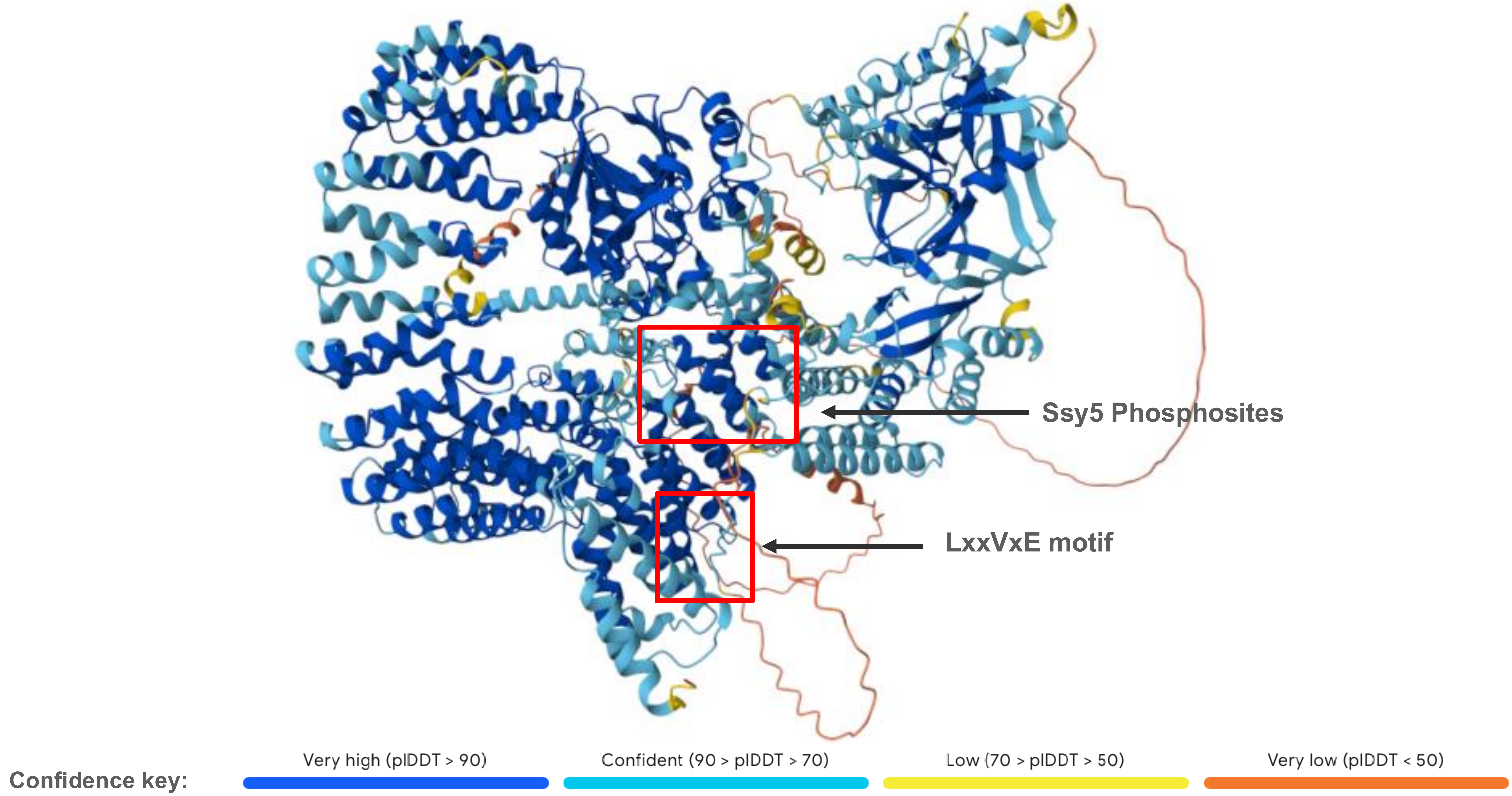

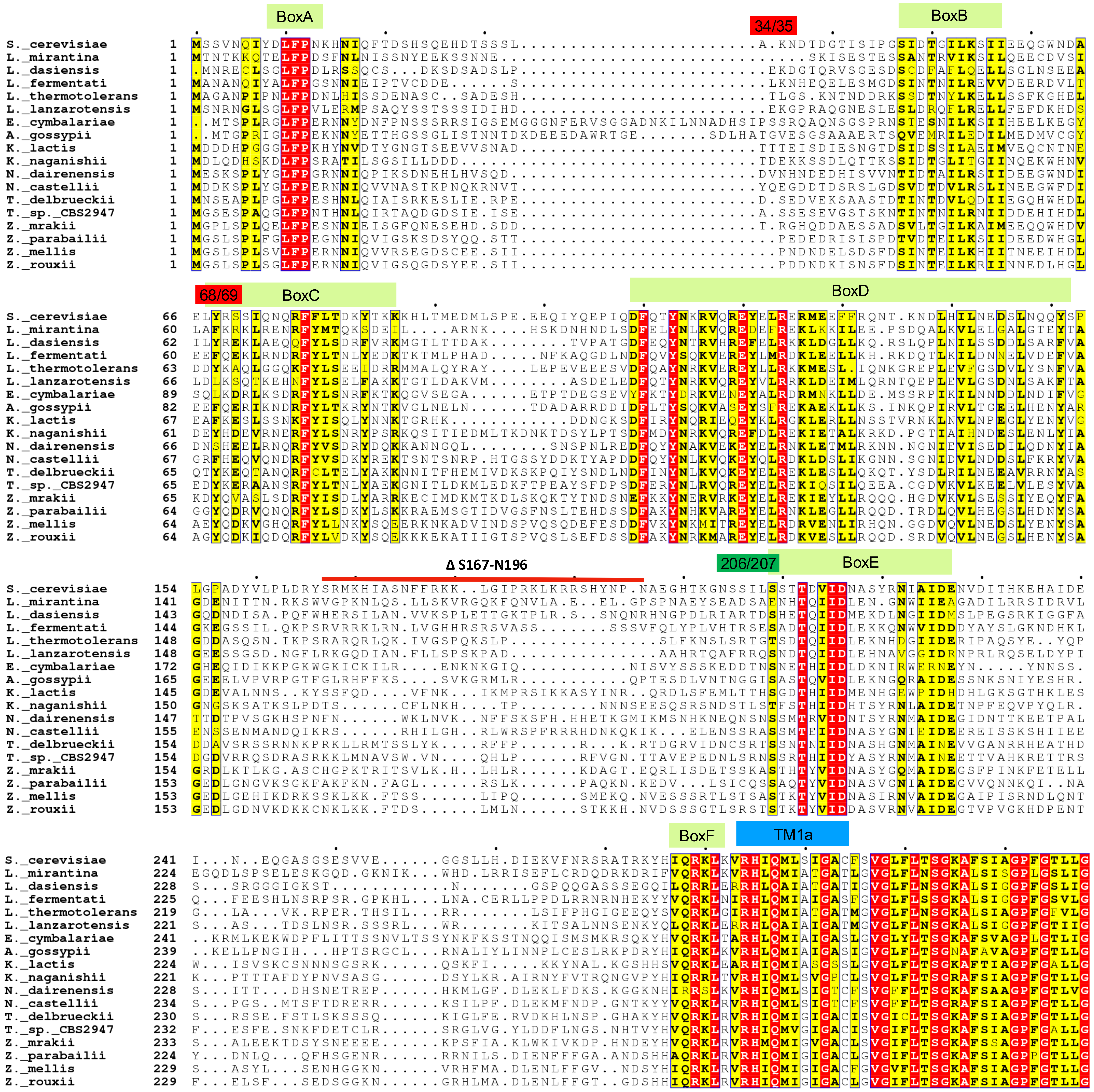

Fig. S20. Alignment of the N-terminal domains of 18 Ssy1s from *Saccharomycetaceae*

Fig S21. BoxE is located close to IL2-3 and the C-terminal latch.

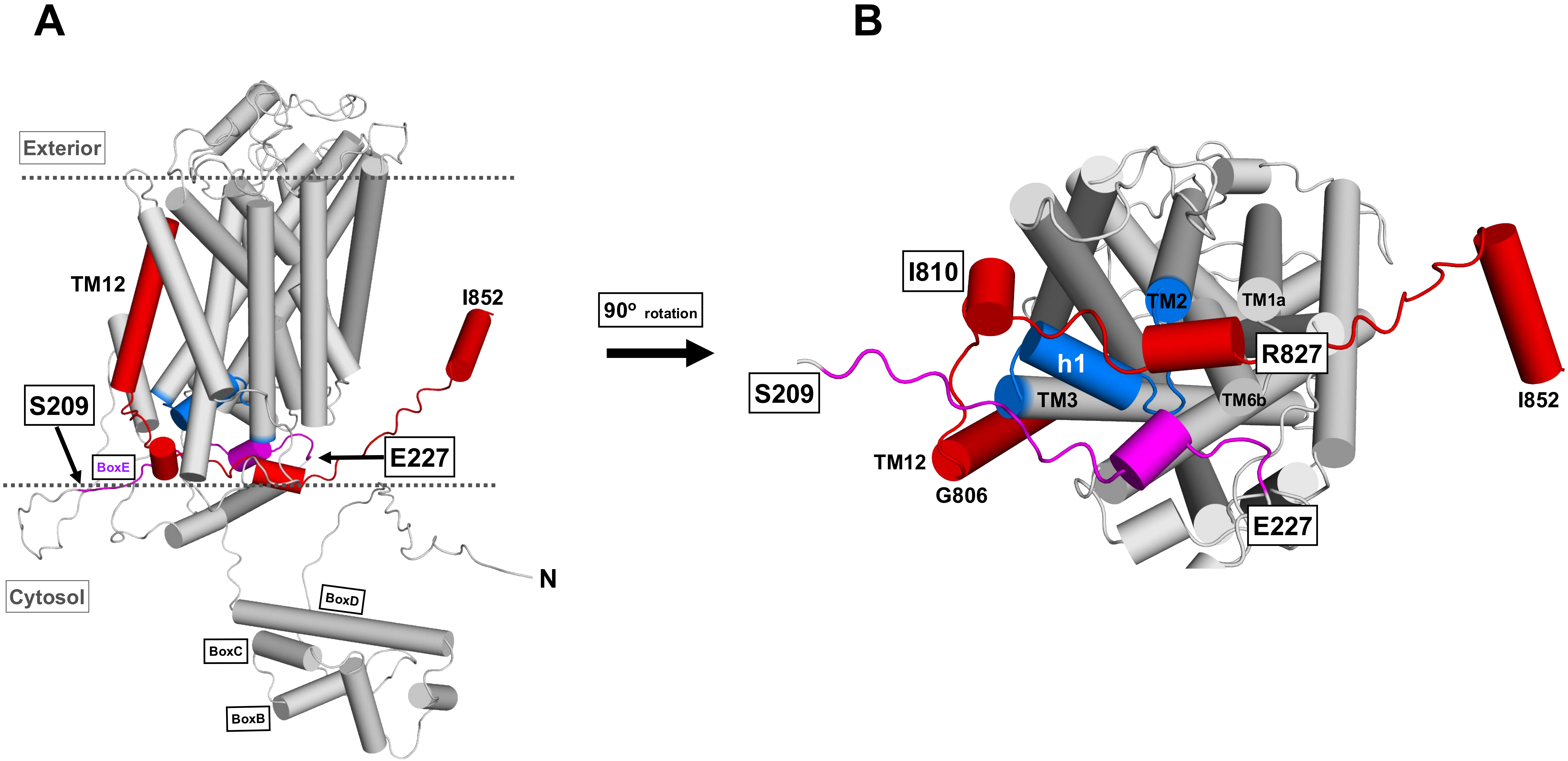

Fig. S22. Interaction between the LFA motif in Ptr3 and the prodomain of Ssy5

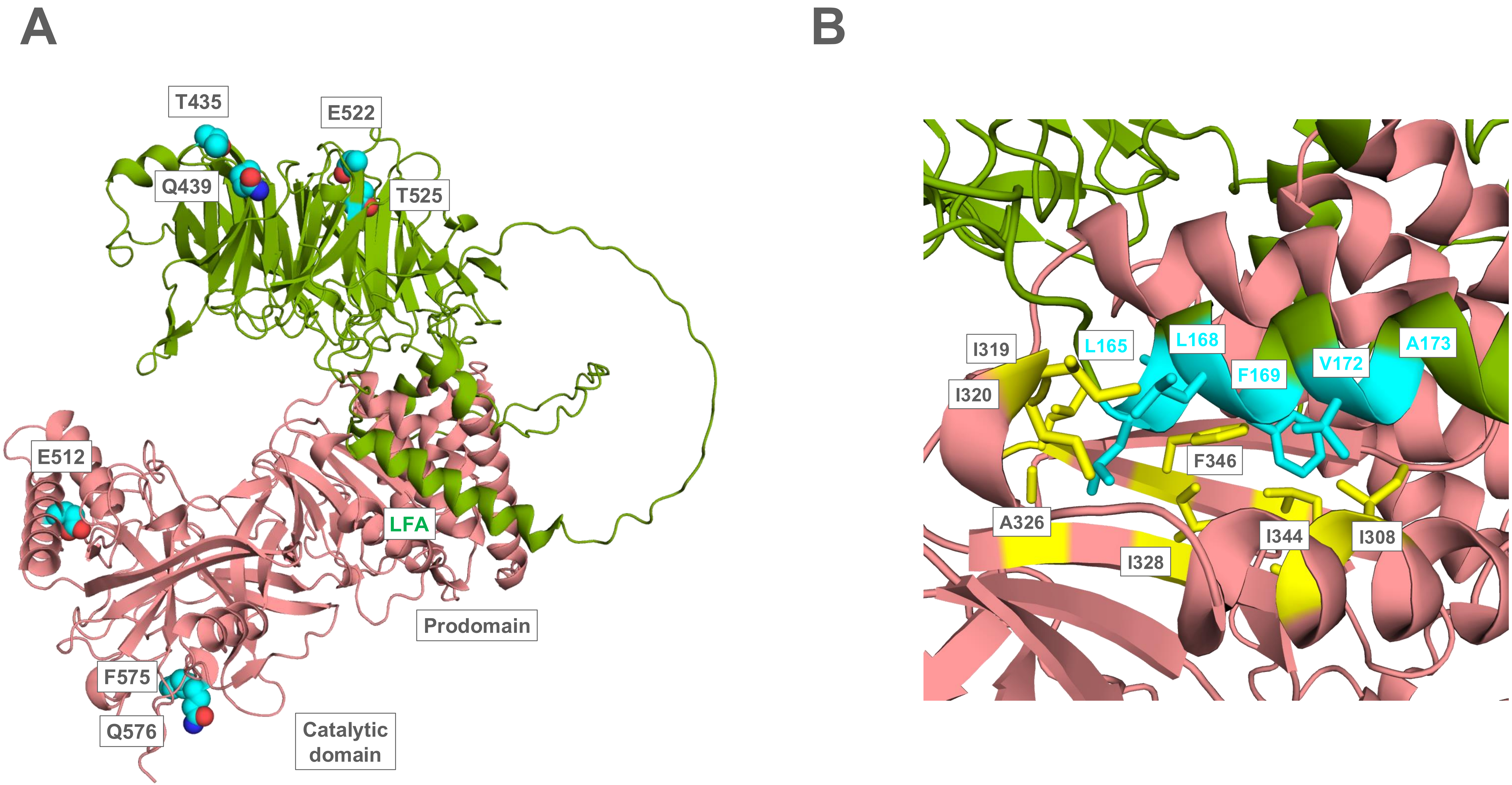

Fig. S22C. Model of Ptr3-Ssy5 interaction showing per-atom confidence estimates

C

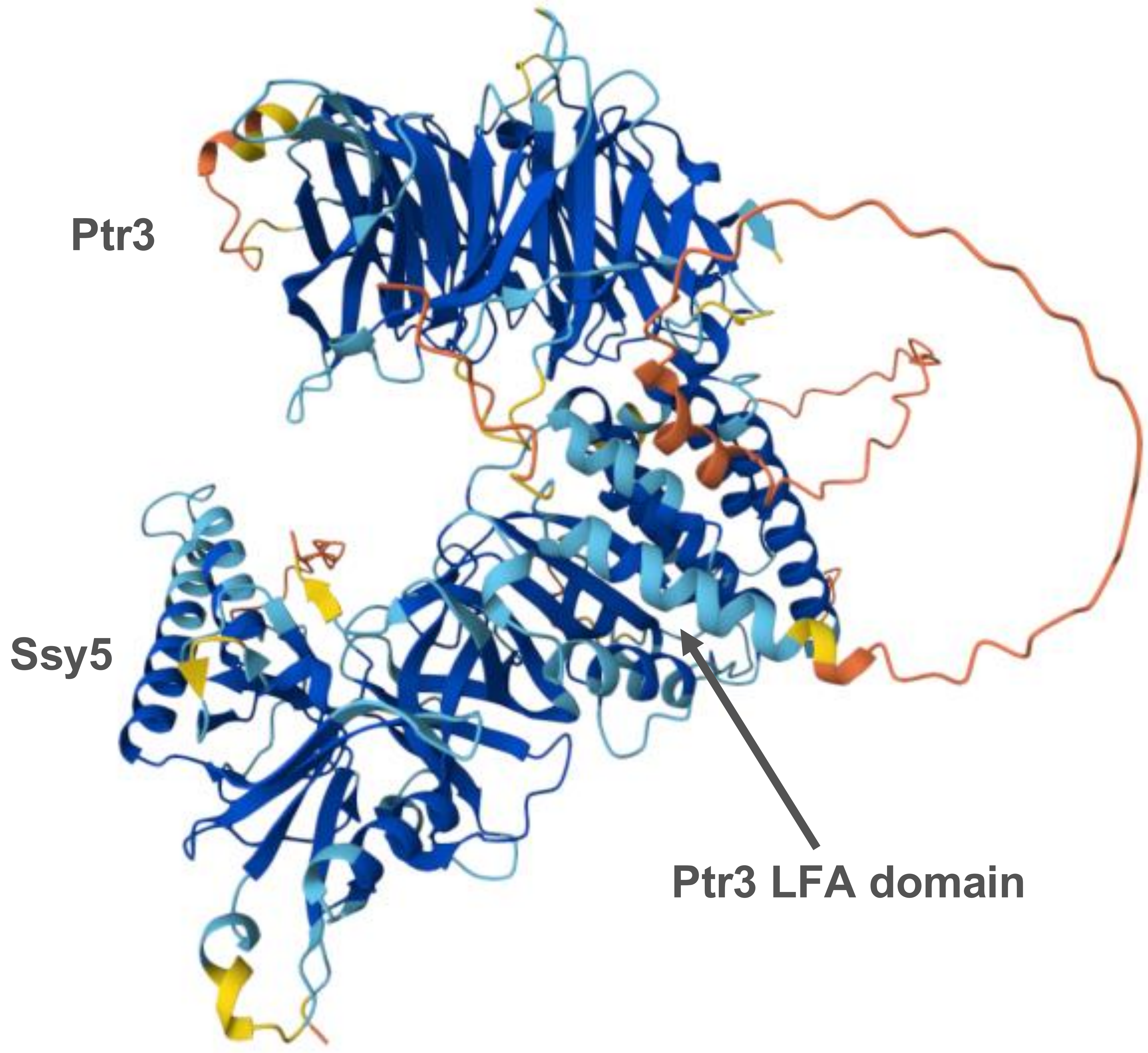

Confidence key: Very high (pIDDT > 90) | Confident (90 > pIDDT > 70) | Low (70 > pIDDT > 50) | Very low (pIDDT < 50)

Fig. S23 Interaction of BoxD and BoxE in Ssy1 with blade 3 in Ptr3

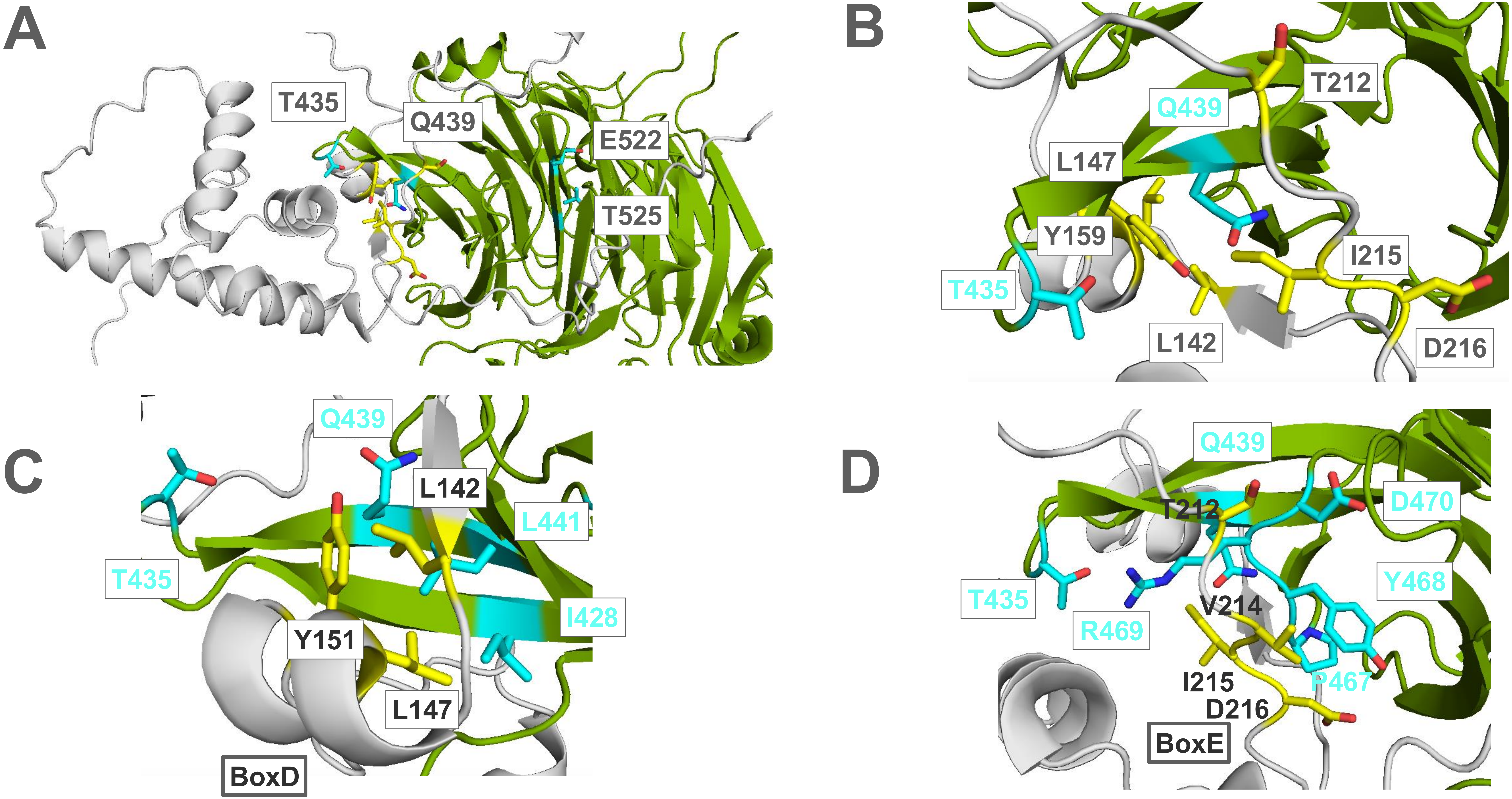

Fig. S23 Interaction between BoxD and BoxE in Ssy1 with blade 3 in Ptr3

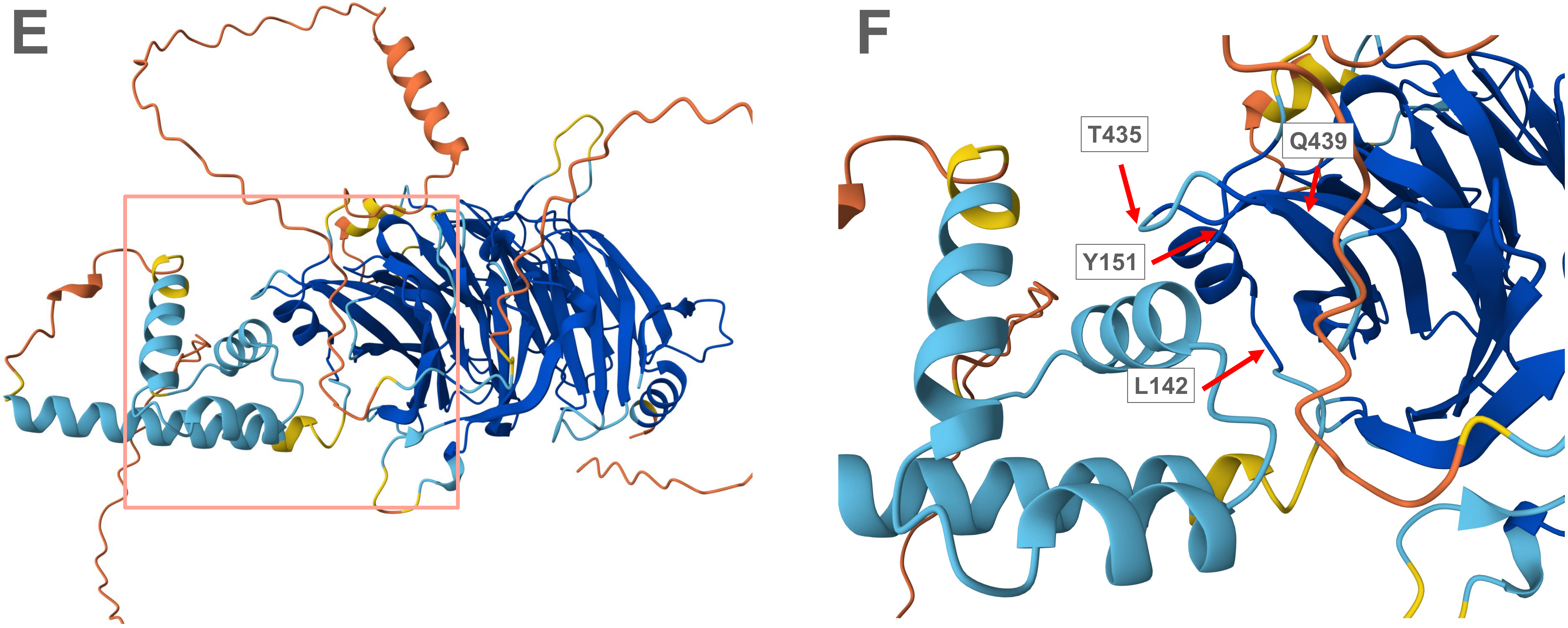

Confidence key: Very high (pIDDT > 90) Confident (90 > pIDDT > 70) Low (70 > pIDDT > 50) Very low (pIDDT < 50)

Fig. S24. BoxA in the Ssy1 N-terminal domain likely interacts with the catalytic domain of Ssy5

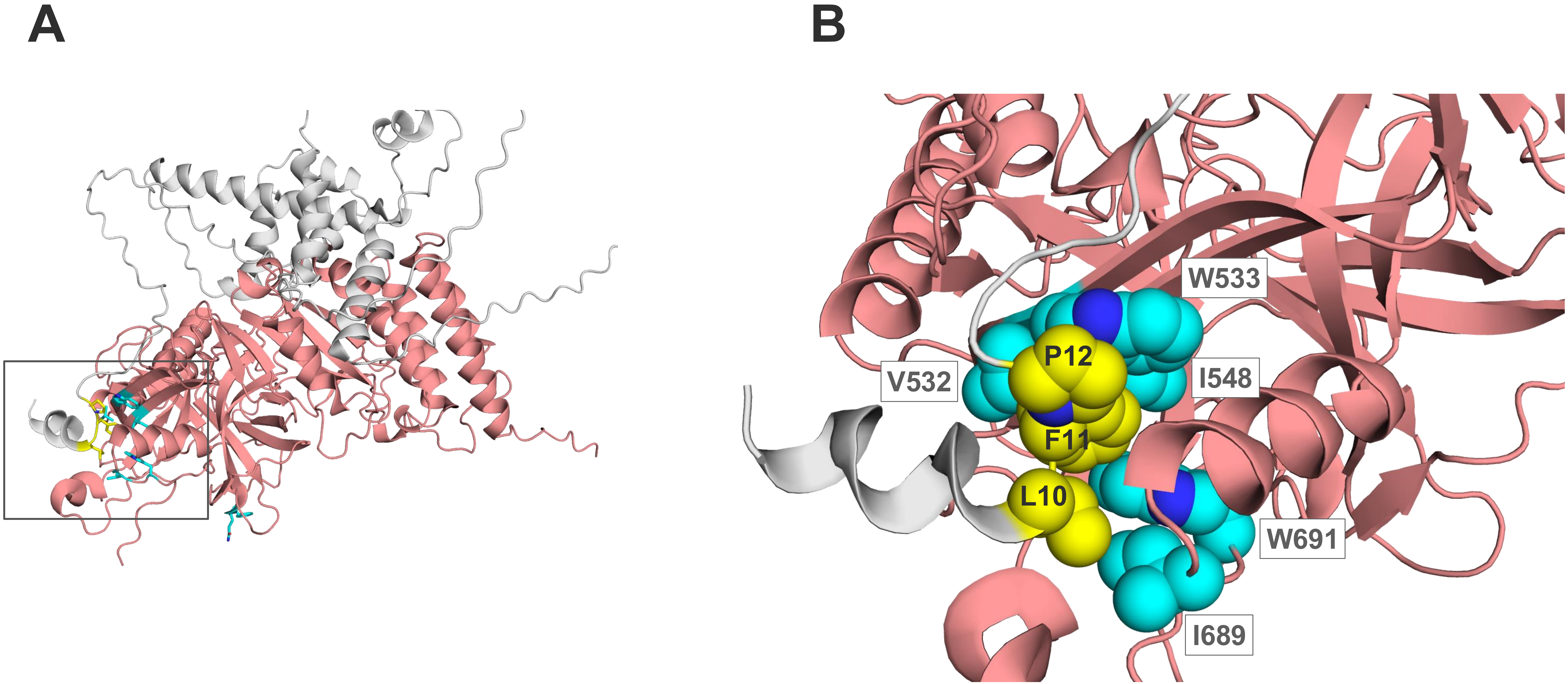

Fig. S24. Model of Ssy1-Ssy5 interaction showing per-atom confidence estimates (pIDDTs)

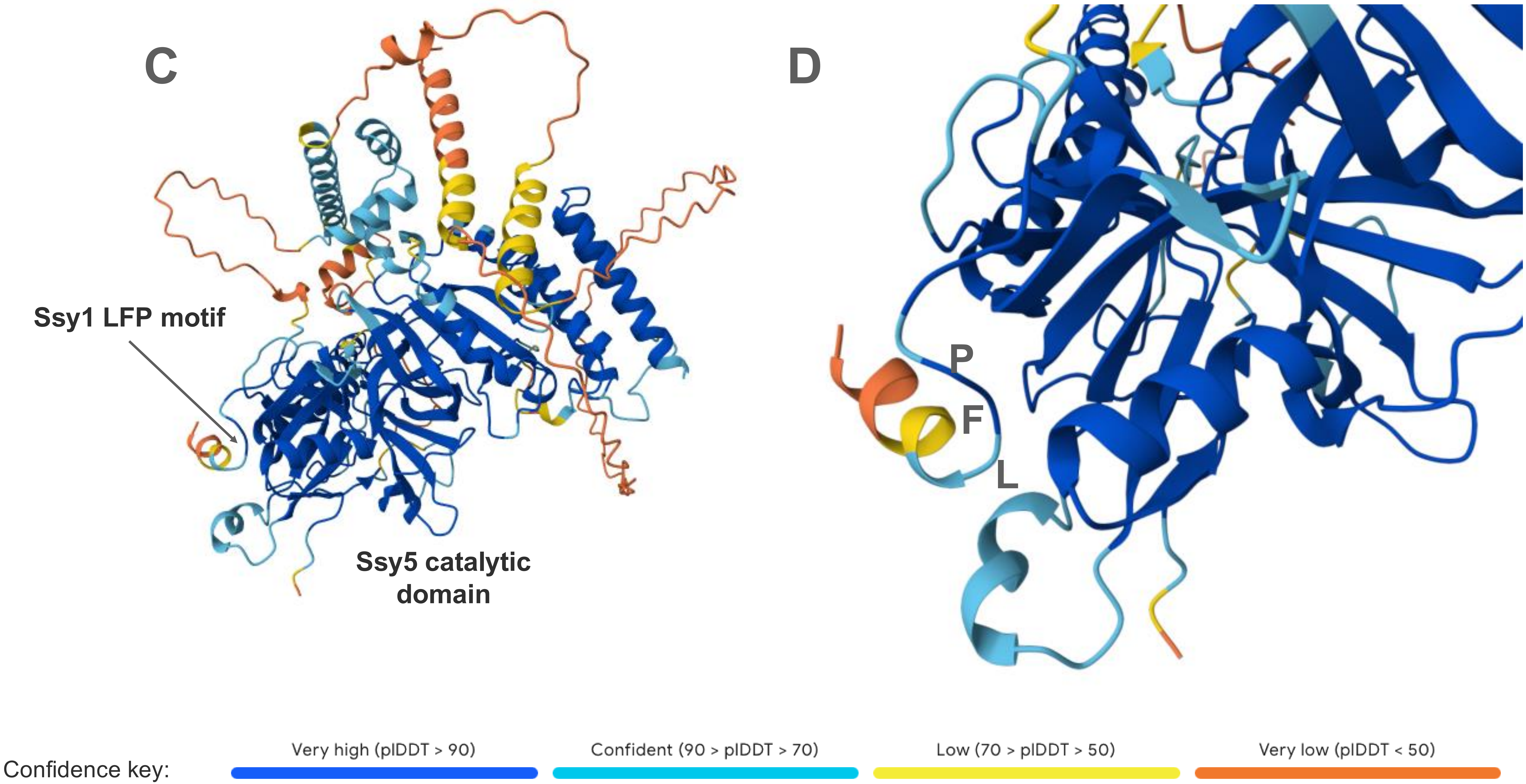

Fig. S25. Per-atom confidence estimates of the Ssy1-Ptr3-Ssy5 complex

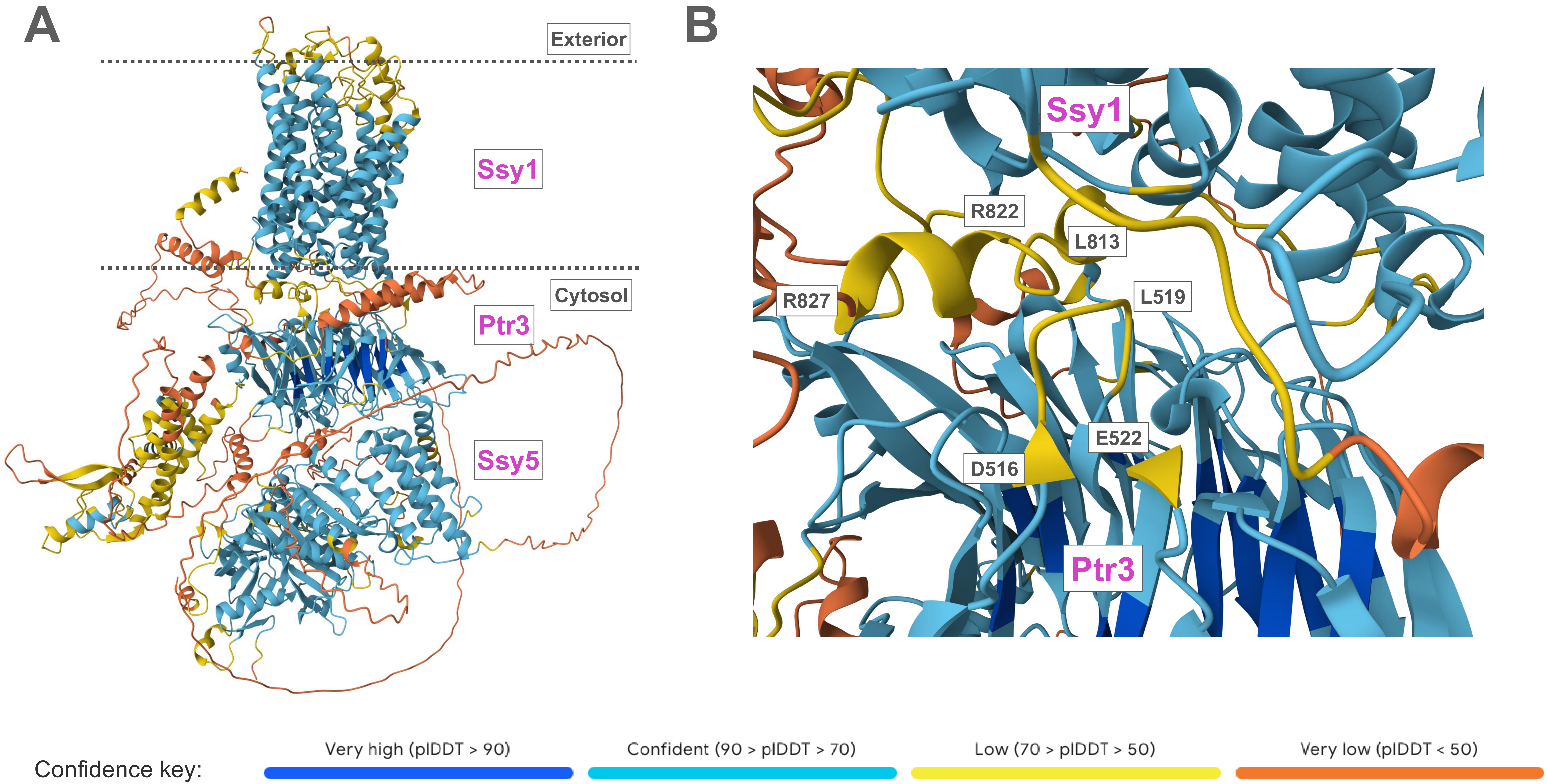

Fig. S26. Interaction between Yck1 and BoxD in Ssy1

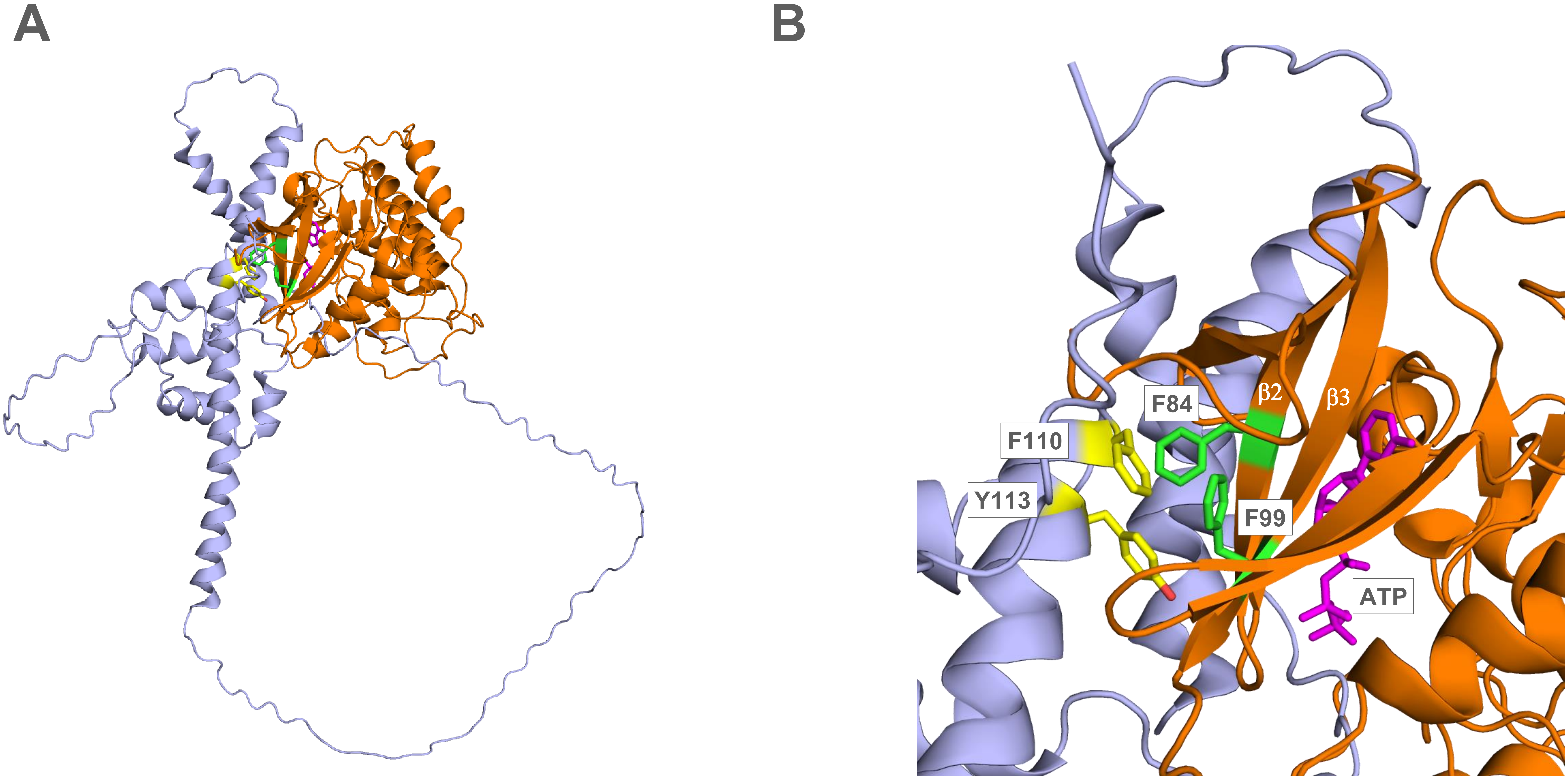

Fig. S26. Model of Yck1 and Ssy1 interaction showing per-atom confidence estimates (plDDTs)

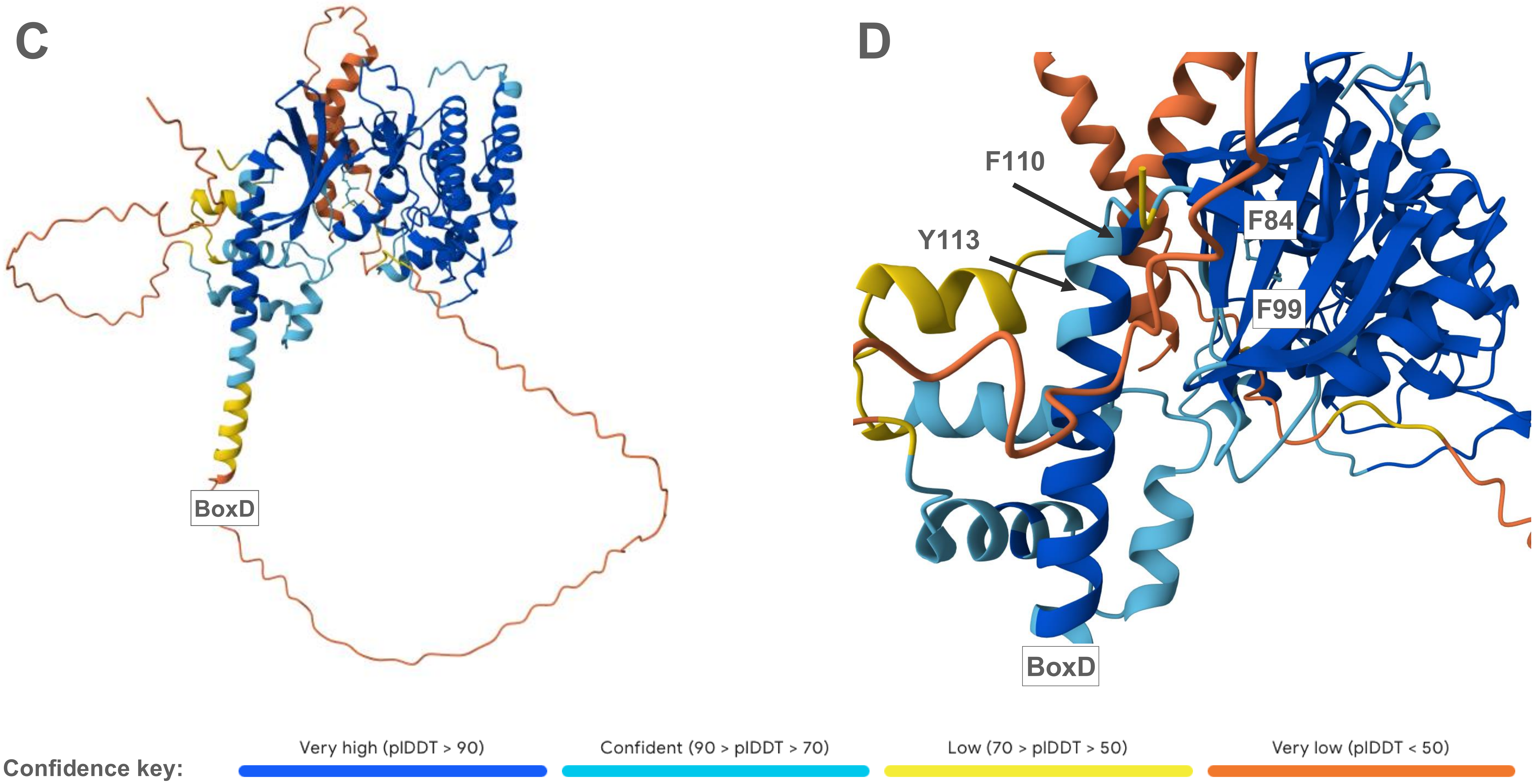

Fig. S27. Alignment of 50 Yck1 orthologs highlighting residues F84 and F99

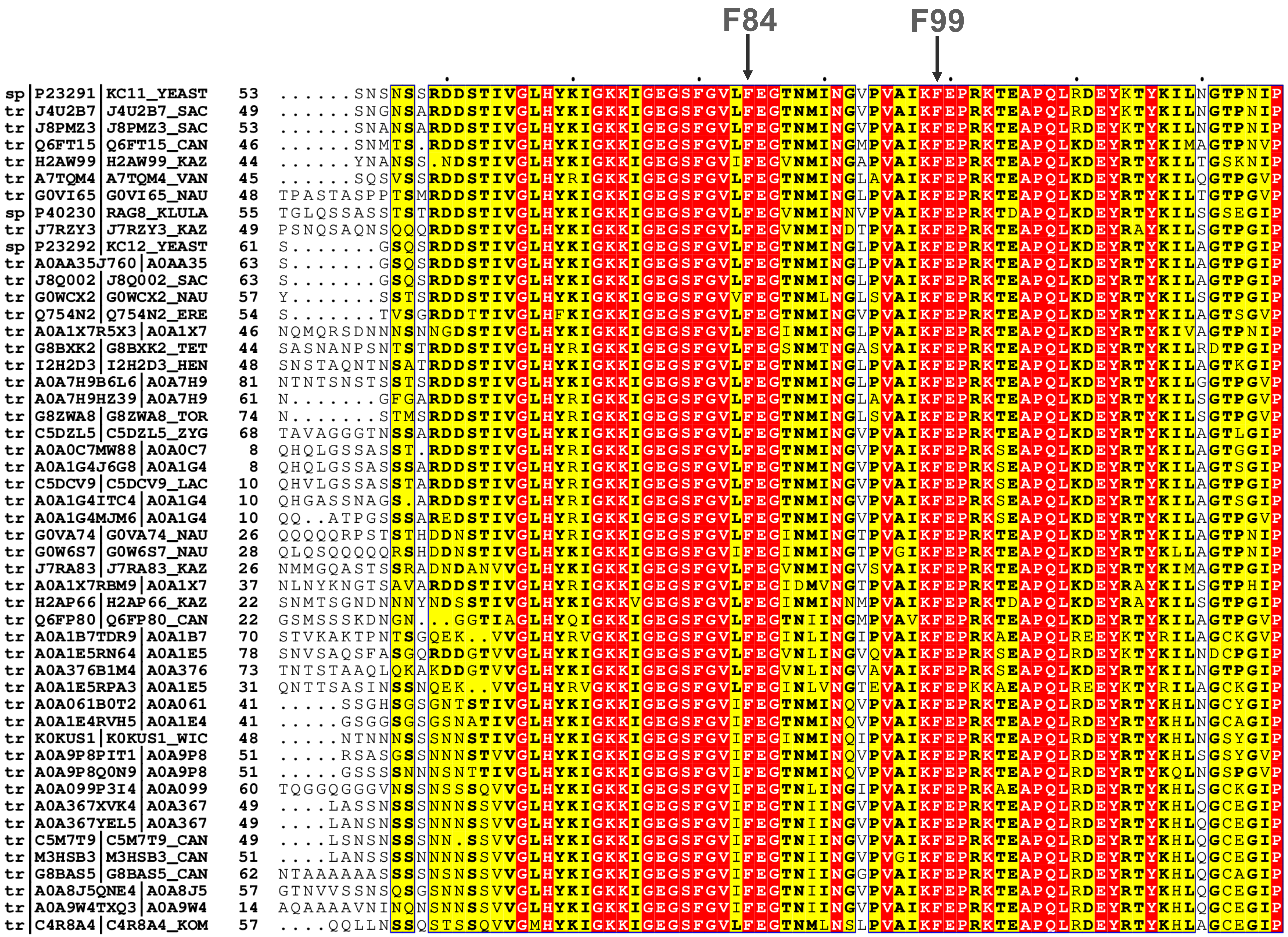

Fig. S28. Proposed Ptr3 phospho-sites are located in the Ssy1-Ptr3 interface.

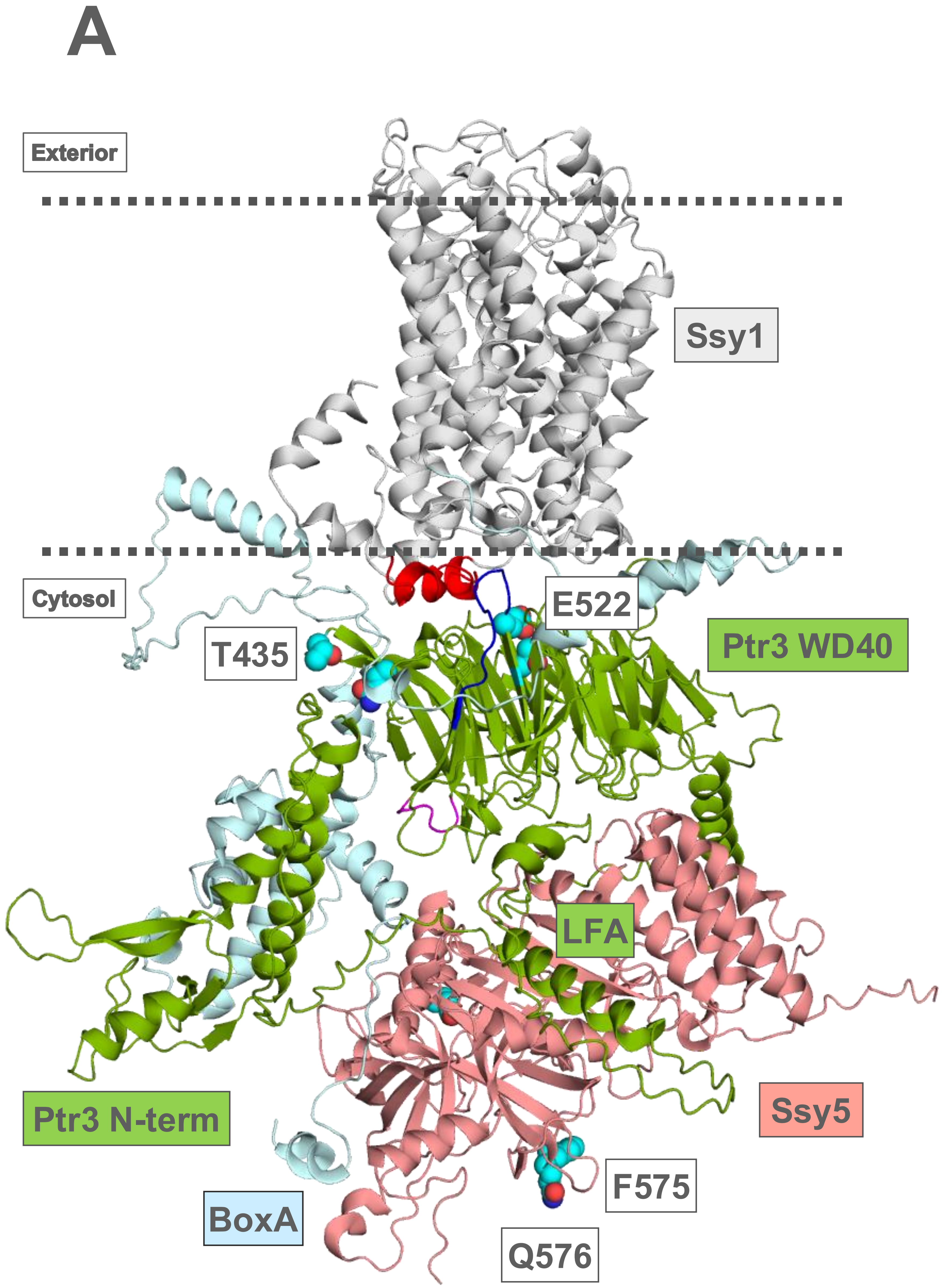

Fig. S29. Residues T517 and S521 in the Ptr3 phosphosites loop are close to D817 and D819 in the Ssy1 latch

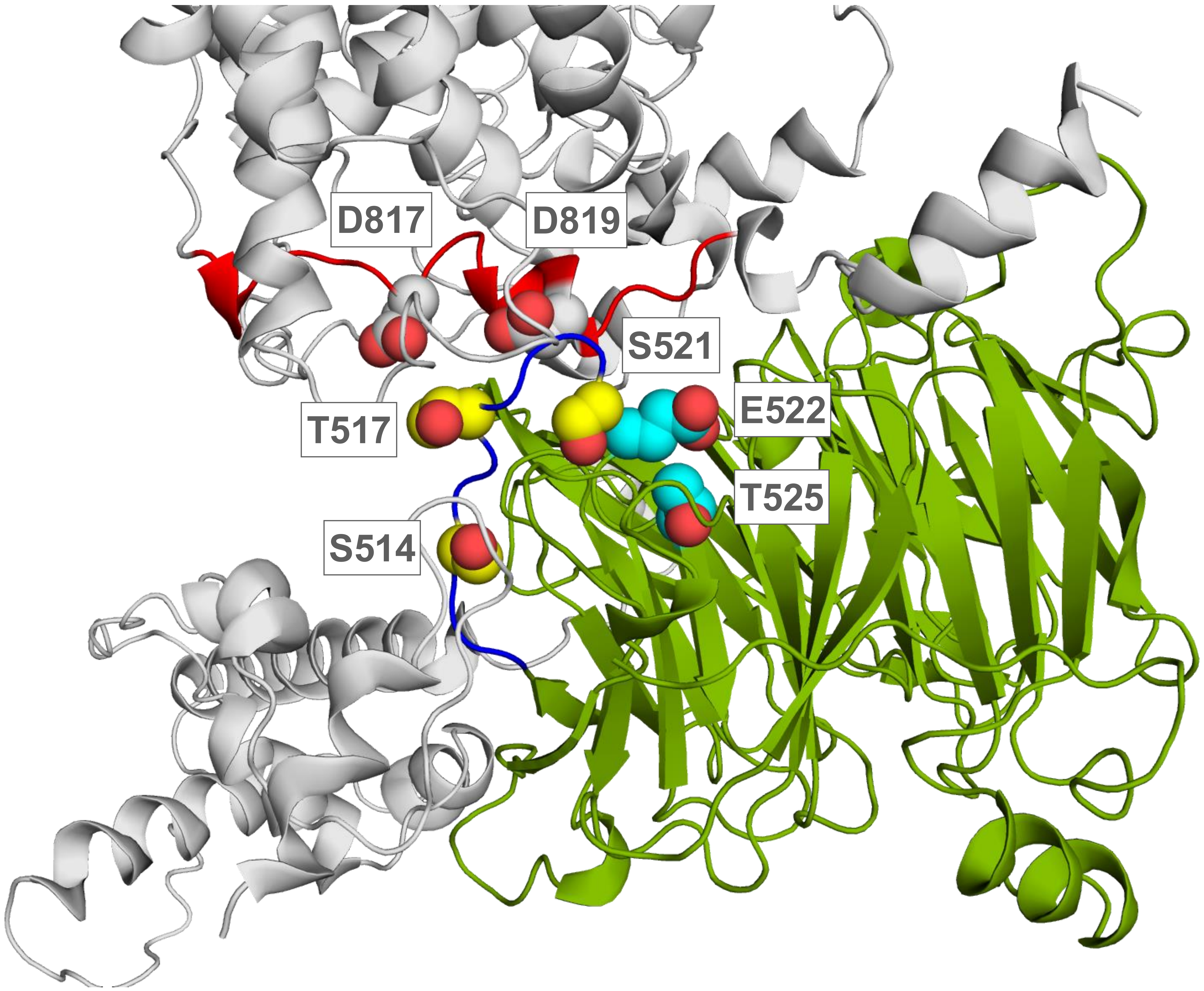

Fig. S30. Displacement of Ptr3 from Ssy1 by phosphorylation

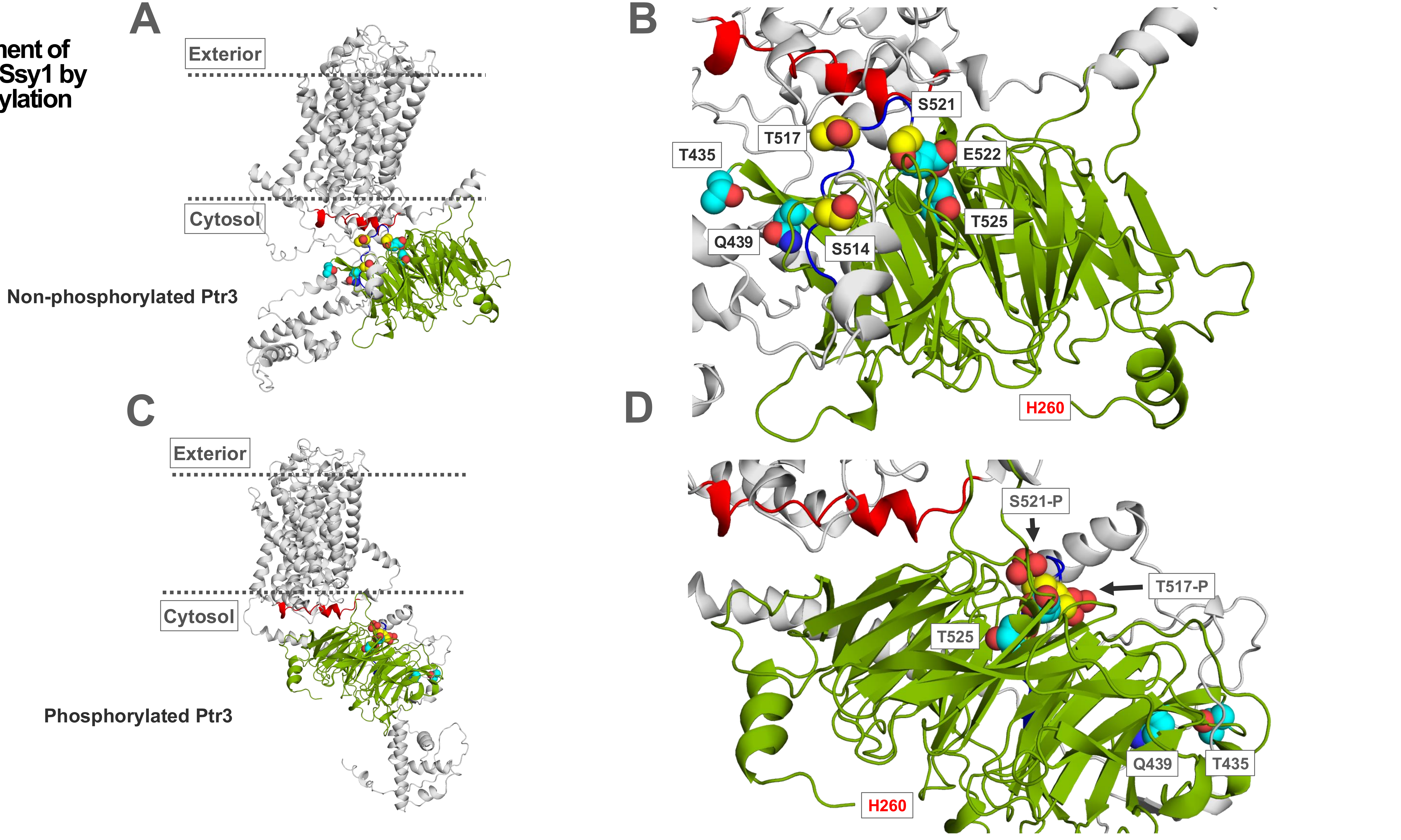

Fig 30. Per-atom confidence estimates of the Ssy1-Ptr3 complex

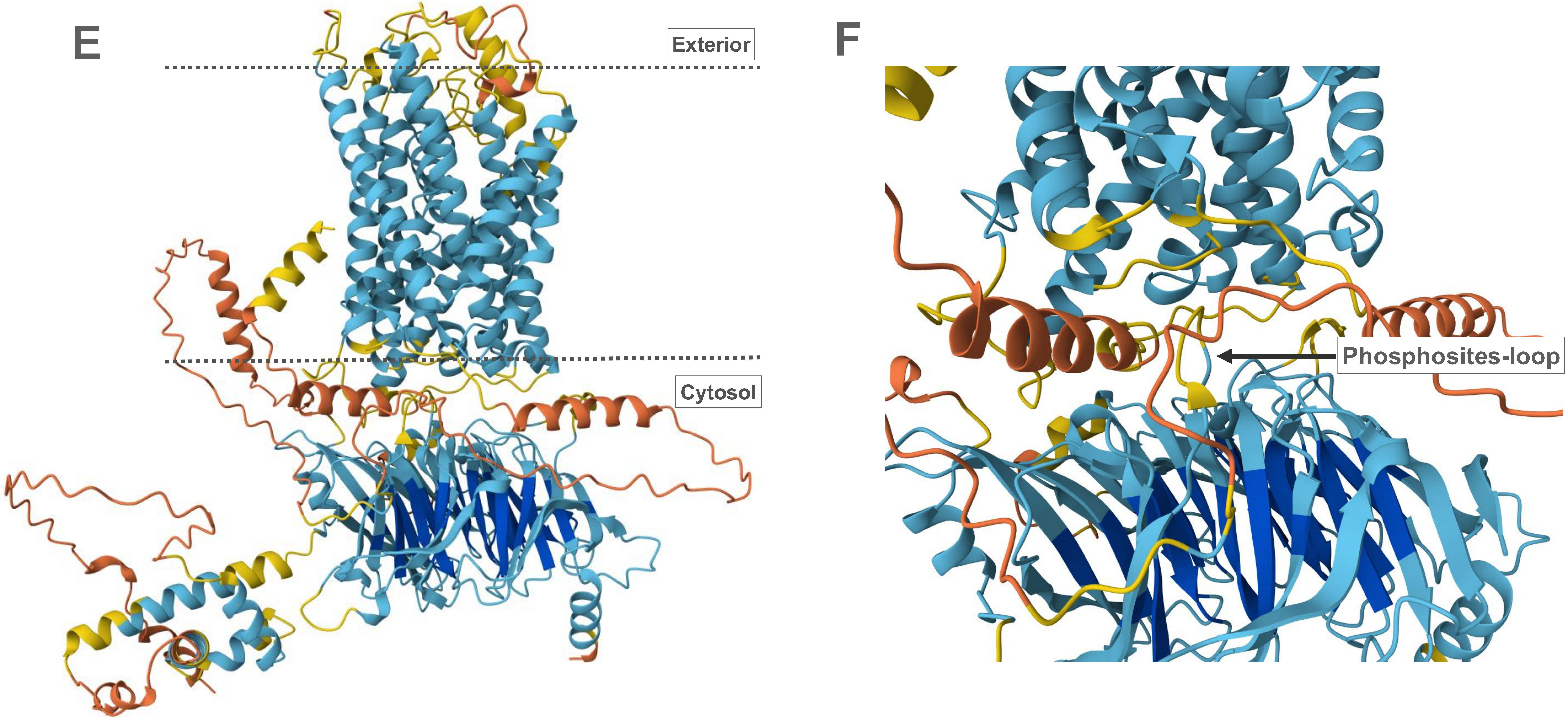

Confidence key:

Very high (pIDDT > 90)	Confident (90 > pIDDT > 70)	Low (70 > pIDDT > 50)	Very low (pIDDT < 50)

Fig. S31. Dimerization of the N-terminal of Ptr3

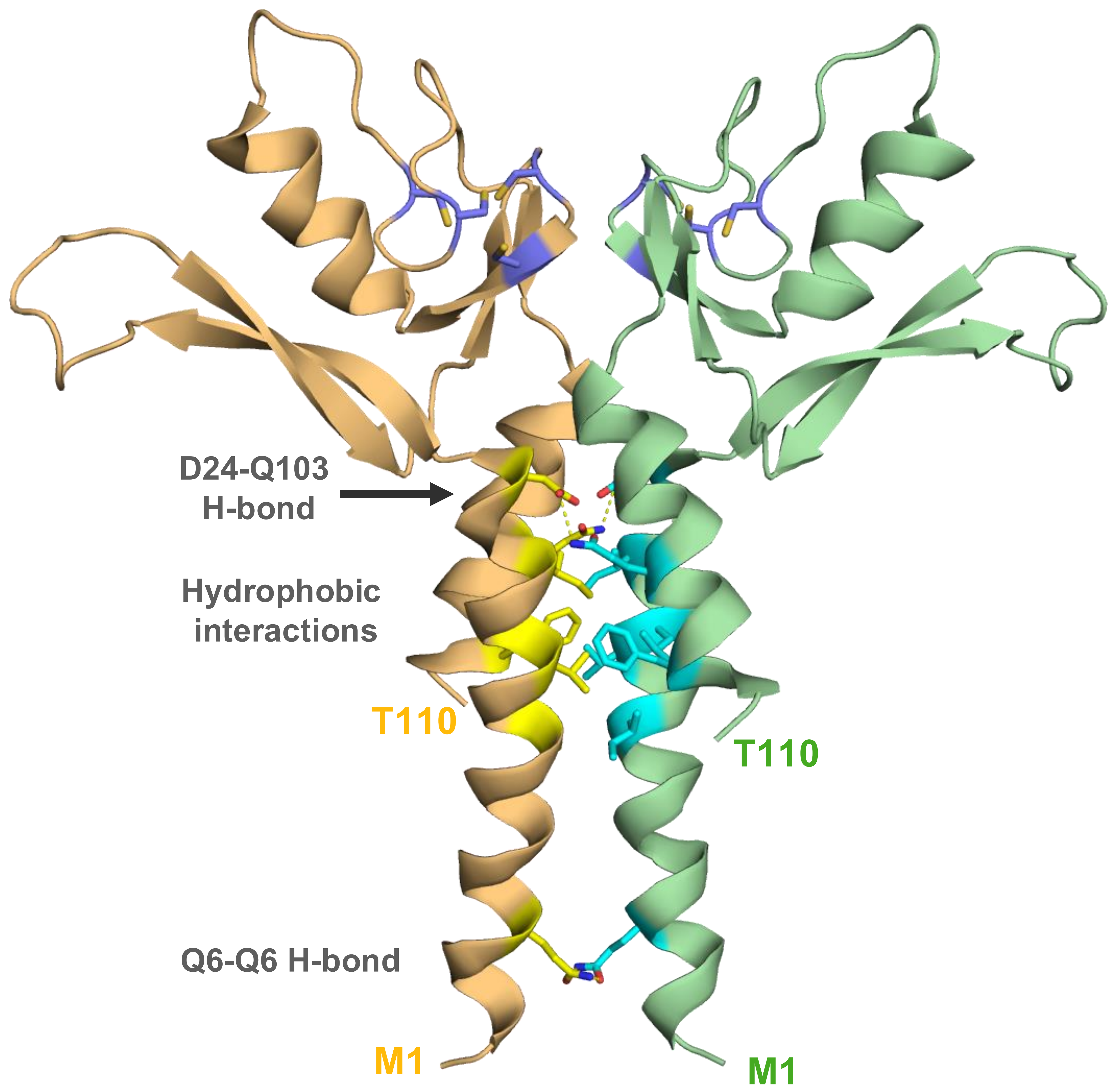

Supplement: jkaf312_Supplementary_Data [file jkaf312_supplementary_data.zip › Supplementary_Figures_G3-2025-406353/G3-2025-406353R1_SuppFigs.pdf]
