# Supplementary material for: Structural bases of signal generation and transduction by the SPS amino acid sensor of Saccharomyces cerevisiae
Source: G3 (Bethesda). 2025 Dec 24;16(3):jkaf312. doi: 10.1093/g3journal/jkaf312 (PMC12958826; doi:10.1093/g3journal/jkaf312)
Supplement: jkaf312_Supplementary_Data [file jkaf312_supplementary_data.zip › Supplementary_Figure_Legends_and_Tables_G3-2025-406353.docx]

**Supplementary Figures Legends and Tables**

**Fig. S1.** **Alignment of the *S. cerevisiae* amino acid receptor Ssy1 with *S. cerevisiae* YAT family amino acid transporters.** Clustal Omega and ESPript were used for alignment. Residues with similar physico-chemical characters are written with black bold characters and boxed in yellow when conservation is above a threshold of 60% per column. Conserved residues are written with white characters and boxed in red. The positions of seven constitutive mutations are indicated with green dots above the sequence, while a hypo-responsive mutation is marked with a red dot. IL2-3 is indicated by a red bar, while EL5-6 and EL7-8 are indicated with blue bars. Ligand binding motifs GTG (296-SVG-298 in Ssy1) and (F/Y)(S/A/T)(F/Y)xGxE (512-FAFSGVE-518 in Ssy1) are indicated with green bars.

**Fig. S2.** **Alignment of 37 fungal Ssy1 orthologs.** A BLAST sequence similarity search with the *S. cerevisiae* Ssy1 sequence was performed at the UniProt portal (<https://www.uniprot.org>) in order to obtain sequences of Ssy1 homologs. Among the 50 top hits obtained, which were all fungal, we selected 36 Ssy1 homologs on the basis of having long N-terminal domains, omitting the seven closely related *Saccharomyces sensu stricto* orthologs already considered in Fig. S3. Alignment with the *S. cerevisiae* Ssy1 sequence shows N-terminal domains ranging in size from 59 to 398 residues with an average of 254 residues. The alignment uncovers low sequence homology between the N-terminal domains, which lack fully conserved residues. In contrast, the transmembrane core shows high homology, with 35 residues fully conserved and many highly conserved regions. EL5-6 (A447 to S494, *S. cerevisiae* numbering) is highly conserved, whereas EL7-8 (I560 to L624) is less conserved, with a central segment of roughly 35 non-conserved residues interrupted by a highly conserved cysteine residue (C596). The non-conserved residues are flanked by two partially conserved regions: Y561 to P572, neighboring TM7, and T609 to S629, neighboring TM8.

Residues with similar physico-chemical properties are indicated with black bold characters in yellow boxes when conservation is above a threshold of 60% per column. Fully conserved residues are shown as white characters in red boxes. The positions of seven constitutive mutations are indicated with green dots above the Ssy1 sequence, while a hypo-responsive mutation is marked with a red dot. The N-terminal domain in *S. cerevisiae* Ssy1 contains residues M1 to K282, IL2-3 residues I340 to E355 (red bar), EL5-6 residues A447 to S494 (blue bar), EL7-8 residues I560 to L624 (blue bar), SVG motif (green bar).

**Fig. S3. Positions of residues fully conserved in YAT transporters and Ssy1.** The 25 conserved residues are indicated in magenta in this high-confidence Phyre2 structure model of Ssy1 based on *Carnobacterium* amino acid transporter BasC (PDB 6F2W, Table S2). Most of the 25 conserved residues are located close to either the extracellular face or the cytoplasmic face of Ssy1, twelve of them in loops. Only K431 in TM5 and E518 in TM6b are centrally located, close to the ligand binding site. Both are conserved in equivalent positions in Ssy1 orthologs (Fig. S2). Mutagenesis of equivalent residues in amino acid transporters shows that they have key functions in transport. Thus, changes of BasC-K154, MjApcT-K158 and GkApcT-K191, all corresponding to Ssy1-K431, abolish transport (Shaffer et al. 2009) (Jungnickel et al. 2018) (Errasti-Murugarren et al. 2019). Studies of BasC-K154 suggest that the positively charged side chain of K154 supports the intrinsic gating of TM1a and proper substrate binding (Fort et al. 2025). Transport is also abolished by mutations affecting residues equivalent to Ssy1-E518 in YAT family members Bap2 and Tat2 (i.e., Bap2-E305 and Tat2-E286) (Kanda and Abe 2013) (Usami et al. 2014) and in the *Escherichia coli* antiporters AdiC and GadC (i.e. AdiC-E208 and GadC-E218) (Gao et al. 2009) (Ma et al., 2012) also leads to lack of transport.

**Fig. S4. Displacement of V694 during the shift from an outward open to an occluded conformation. A.** Side view of an alignment of Ssy1 models based on AdiC (7O82) in an outward-open structure (grey) and AdiC (3L1L) in an occluded structure (light orange). The alignment illustrates the displacement of TM10 and V694 in EL9-10. V694 is shown as magenta sticks. **B.** Close-up view of TM9, EL9-10 and TM10.

**Fig. S5. Modeling of extracellular loops suggests a cap that can close the extracellular gate.**

**A.** Side view of an outward open AlphaFold model of Ssy1 (Q03770) presented with cylindrical helices colored blue through red from TM1; EL7-8 (I560 to L624) is highlighted in magenta. **B.** Close-up view with TM helices in grey and EL5-6 and EL7-8 highlighted in green and magenta, respectively. The positions of T382 in TM3 and V694 in EL9-10 are indicated with red spheres. A potential disulfide bridge between C596 in EL7-8 and C625 in TM8 is shown as cyan sticks with yellow sulfur atoms. The predicted helices h2 (P572 to K584) and h3 (S613 to N621) in EL7-8 are indicated. **C.** Close-up view highlighting the segment from I574 to G608 (orange), which has no counterpart in YAT transporters.

Since the Phyre2 modeling only accounts for about 30 residues of each of the loops EL5-6 and EL7-8, we used the AlphaFold Ssy1 model, which includes a structure prediction of the full length of Ssy1 (Panel A). We consider this model to have an outward-open conformation, because structural alignments of the AlphaFold model with Ssy1 models based on AdiC (PDB code 7O82) (Ilgü et al. 2021), AdiC (3L1L) (Gao et al. 2010) and LAT1 (6IRT) (Yan et al. 2019) in outward-open, occluded, and inward-open conformations, respectively, yield all-atom Root Mean Square Deviations of 2.393, 6.967 and 6.643, respectively. In addition, TM1b and TM6a are clearly in their swung-out position in accordance with an outward-open conformation (Panel A). The predicted structure of EL5-6 (A447 to S494) is estimated by AlphaFold to be reliable. EL7-8 (I560 to L624) has two α-helical segments here designated h2 (S575 to K584) and h3 (S613 to N621) (Panels A and B), both of which have reliable predicted structures. In contrast, the proposed structure of the stretch from D589 to Y611, connecting h2 and h3, is of low confidence. The additional segment of EL7-8 comprising residues I574 to G608, which is absent in YATs (Fig. S1), forms helix h2 and a long loop structure (Panel C).

Conserved C596 and C625 likely form a disulfide bond between EL7-8 and TM8. Residues C596 in EL7-8 and C625 in TM8 are 100 % conserved in Ssy1 orthologs (Fig. S2), whereas none of these two cysteine residues are present in YAT family amino acid transporters (Fig. S1). In the model generated by AlphaFold, both C596 and C625 are within a confident structure, and their closeness suggests a disulfide bond between them (Panel B). This presumed, conserved bridge will attach EL7-8 to TM8 in a way that could be important in signaling.

**Figure S6: Pi-pi interactions between Ssy1 F333 and F521 favor a closed intracellular gate. A.** Detail of intracellular view of a model of Ssy1 in an outward open conformation based on AdiC (7O82); helices are shown in grey with highlighting of TM1a (dark blue), TM2 (marine blue), and TM6b (green). The potential of the side chains of F333 and F521 to engage in a pi-pi interaction that anchors TM2 to TM6b is apparent. **B.** Corresponding detail of a model of Ssy1 based on LAT1 (6IRT) in an inward open conformation. TM6b has been substantially displaced, and the pi-pi interaction between F333 and F521 is absent.

**Fig. S7.** **A hydrophobic cluster at the bottom of the binding pocket of Ssy1 likely closes the intracellular gate.** **A.** Extracellular close-up view of an Ssy1 model based on an outward open structure of AdiC (3OB6) with bound arginine shown as yellow sticks. A cluster of hydrophobic side chains is shown as grey spheres. **B.** Extracellular close-up view of an Ssy1 model based on an outward-facing structure of LAT1 (7DSQ) bound with the inhibitor 3,5-diiodo-L-tyrosine shown as yellow sticks. **C.** Intracellular close-up view of an Ssy1 model based on an inward-open structure of LAT1 (6IRT) bound with the inhibitor BCH shown as yellow sticks. Helices are shown transparent, colored blue through red from the N-terminus.

**Fig. S8. Alignment of the Ssy1 transmembrane core of 18 *Saccharomycetaceae* orthologs.** Residues with similar physico-chemical properties are indicated with black bold characters in yellow boxes when conservation is above a threshold of 60% per column. Fully conserved residues are shown as white characters in red boxes.

**Fig. S9. Binding of leucine in an Ssy1 model based on a LAT2.** **A.** Binding of leucine (yellow sticks) shown in an Ssy1 model based on a LAT2 in an inward open structure with bound leucine (7CMH). The swung-out of helices TM1a and TM6b are indicated. The side chain of leucine is oriented towards the cytosol. **B.** Close-up showing details of leucine binding. The α-carboxyl of leucine bound in Ssy1 likely forms hydrogen bonds to the main chain amide groups of S296 and G298 in the TM1 loop, while the α-amino group forms hydrogen bonds to main-chain carbonyls of C294 in TM1a and S515 in the TM6 loop. F512 in TM6a stabilizes the side chain of leucine. This is in accordance with the general binding mode of amino acids to transporters with a LeuT fold shown in Table S3, in which binding of ligands in nine APC family amino acid transporters are matched with Phyre2-generated models of Ssy1 based on the ligand-bound structures. Further this analysis shows that the main-chain amide groups of S296, V297 and G298 in the TM1 loop of Ssy1 likely form hydrogen bonds with the α-carboxyl of the ligand. The three residues (296-SVG-298) correspond to the highly conserved GTG motif in the *bona fide* transporters in the YAT family (Gournas et al. 2016) (Fig. S1). However, the valine residue in this motif in Ssy1 is a major difference. Valine occurs in this position in 34 of 37 Ssy1 orthologs (Fig. S2), suggesting that residue V297 somehow provides a feature expedient for the receptor function, contrary to the GTG motif in the YAT transporters, which may be required for swift substrate transport. Fast TM1 loop dynamics is thus believed to be required for the conformational switches necessary in transporters (Gao et al. 2009). Indeed, mutations that introduce bulky, hydrophobic residues into the corresponding GTG motifs of *S. cerevisiae* Tat2 (G97V) (Kanda and Abe 2013), *S. cerevisiae* Bap2 (G110V) (Usami et al. 2014), and *Danio rerio* SLC38A9 (T133W) (Wyant et al. 2017) eliminate amino acid transport. It is conceivable that the bulky side chain of V297 not only slows down the loop dynamics but also in some way contributes to the inability of Ssy1 to directly interconvert its states D and H (Fig. 2).

F512 likely functions as a thin gate, similarly to W202 in AdiC (Gao et al. 2010), which contributes to closure of the extracellular gate. F512 is highly conserved in YAT transporters (Fig. S1) and conserved as either F or Y in Ssy1 orthologs (Fig. S2). The importance of a residue with an aromatic side chain in the position corresponding to Ssy1-F512 has been confirmed by mutational analysis of several amino acid transporters in the APC family. For example, the mutant forms AdiC-W202L, LAT1-F252A, LAT2-F243A, and b^0,+^AT-W230R have drastically reduced transport activity (Fang et al. 2009) (Lee et al. 2019) (Yan et al. 2020a) (Yan et al. 2020b).

**Fig. S10. The hypo-responsive mutation T639I affects ligand binding and conformational shifts of the TM1 loop. A.** Model of Ssy1 based on AdiC (3OB6) with bound arginine (yellow sticks) in an outward open structure. The α-carboxyl of arginine forms hydrogen bonds to the main-chain amide groups of V297 and G298, and the α-amino group forms hydrogen bonds to main-chain carbonyls of C294 in TM1a, S515 in the TM6 loop, and F512 in TM6a. Note the proximity of side chains of T639 in TM8 and V297 in the TM1 loop. **B.** Interference of the hypo-responsive T639I mutation with substrate binding. Close-up view of the model based on AdiC (3OB6) illustrating substitution of T639 with an isoleucine residue and the resulting steric conflict between the V297 and I639 side chains shown as spheres.

**Fig. S11. Prediction of disorder and structure of Ptr3. A.** Plot of the per-residue disorder of Ptr3 (red curve) analyzed on the Metapredict online (v2.4) portal (Emenecker et al. 2021). Also shown is the predicted AlphaFold structural confidence score (pLDDT) (blue curve) of the Ptr3 model (P43606). The analysis predicts structured N-terminal (R10 to T111) and C-terminal (E264 to K678) domains. The region in between is disordered except for an α−helix (L165 to A179), which has a peak in the structure prediction coinciding with a minimum in the disorder prediction. This region contains the so-called LFA motif involved in Ptr3-Ssy5 interaction (Omnus and Ljungdahl 2013). **B.** AlphaFold model of Ptr3 (P43606) shown as a cartoon illustration with loops and helices colored blue through red from the N-terminus. The LFA motif predicted to form an α-helix and the WD40 repeat region are indicated. **C.** Zoom-in of same structure showing the N-terminal domain with five strictly conserved residues D51, C57, C80, C83, and L97.

**Fig. S12. Alignment of N-terminal regions of 32 Ptr3 orthologs.** A subset of closely related Ptr3 sequences showing a conserved region in the N-terminal. Two stretches of highly/strictly conserved residues, V49 through F66 and C80 through A101, comprising D51, C57, C59, C80, C83, and L97.

**Fig. S13. Sequence alignment of Ptr3 from 46 related fungal species.** Alignment of *S. cerevisiae* Ptr3 with 45 orthologs obtained from a BLAST sequence similarity search with the *S. cerevisiae* Ptr3 sequence at the UniProt portal (<https://www.uniprot.org>). The orthologs were aligned using Clustal Omega and ESPript 3.0. Residues with similar physico-chemical properties are indicated with black bold characters in yellow boxes when conservation is above a threshold of 60% per column. Fully conserved residues are shown as white characters in red boxes.

Various features of *S. cerevisiae* Ptr3 are indicated above the sequence: (i) gain-of-function mutations T435K and Q439R (Poulsen et al. 2005) (green dots); (ii) loss-of-function mutations E522K (ptr3-35 mutant (Abdel-Sater et al. 2004), B. André, personal commun.) and T525A (Liu et al 2008) (red dots); (iii) potential Yck phosphorylation sites, 511-SVRS-514, 514-SIDT-517, and 518-SLES-521 (blue bars) agreeing with the (S/T)XX(S/T) motif (Mok et al. 2010); the three phosphorylation sequence motifs are rather conserved in 35 orthologs but absent in 11 distantly related orthologs; (iv) the LFA motif, involved in Ptr3-Ssy5 interaction (Omnus and Ljungdahl 2013), is present in 32 orthologs (red boxes); (v) the Rts1 binding motif (LxxIxE), conserved in various versions in 24 orthologs, is indicated by blue boxes and orange dots above the *S. cerevisiae* sequence.

**Fig. S14. Structure of *S. cerevisiae* Yck1 and its interaction with the N-terminal of Ssy1. A.** Metapredict analysis of *S. cerevisiae* Yck1 proposes that N-terminal residues (1-60) and the C-terminal residues (480-538) are disordered. The intermediate residues (61-479) are structured and have a typical casein kinase structure (Xu et al. 1995) consisting of an N-terminal lobe with five twisted antiparallel β-strands and an α-helix followed by a C-terminal lobe with nine α-helices. ATP is bound in the cleft between the two lobes. The succeeding ~100 residues are partially structured and contain two glutamine-rich α-helices (AlphaFold), which are followed by a palmitoylation domain in the C-terminus (Roth et al. 2011). **B.** Model of *S. cerevisiae* Yck1 with bound ATP generated in AlphaFold 3 and shown in rainbow mode. ATP bound in the ATP-binding cleft is shown as magenta sticks. The C-terminal residues C537 and C538 are shown as spheres. The structured, glutamine-rich helices (P380 to N421) and (Y433 to T480) are shown in orange and red, respectively. The disordered N-terminal residues (1 to 59) are hidden for clarity. **C.** Close-up of an AlphaFold 3 analysis of interactions between Yck1 and the N-terminal domain of Ssy1. Yck1 is highlighted in rainbow mode, Ssy1 N-terminal (1-283) in pale cyan, ATP in magenta, and F110 and Y113 as yellow sticks. The side chains of conserved residues F110 and Y113 in BoxD of the N-terminal of Ssy1 are facing β3 and β5 sheets of Yck1; the β lobe nomenclature is according to Xu et al., (1995).

**Fig. S15. Prediction of disorder and structure of Ssy5. A.** Plot of the per-residue disorder of Ssy5 (red curve) analyzed on the Metapredict online (v2.4) portal. Also shown is the AlphaFold structural confidence score (pLDDT) (blue curve) of the model (P47002). Residues 1 to 164 are predicted to be disordered, whereas the remaining part of Ssy5 is predicted to form an ordered structure. **B.** AlphaFold model of Ssy5 (P47002) showing residues from R165 to G699 colored blue to red from the N-terminal. The self-cleavage site (A381/A382) is highlighted as magenta spheres, while the catalytic triad (H465, D545, and S640), clustering close to the cleavage site, is shown as grey spheres. Residues subject to constitutive *SSY5* mutations F575V, Q576P and K581N are shown as grey spheres.

**Fig. S16. Alignment of 18 Ssy5 orthologs highlights functional motifs.** The Rts1 binding motif is present in Ssy5 as LxxVxE and is indicated with magenta frames. The motif is found in *S. cerevisiae* Ssy5 and in 16 Ssy5 orthologs of the *Saccharomycetaceae* family. A Rts1 binding site is absent in the corresponding region of *Hanseniaspora uvarum*, which belongs to the *Saccharomycodaceae* family. P1 through P5 designate S/TxxS/T Yck1/2 motifs. Blue dots indicate residues potentially subject to phosphorylation. Positions subject to constitutive mutations are indicated with green dots. The autolytic cleavage site (A381/A382) (Poulsen et al. 2006) is indicated by cyan dots. Lysine residues K200, K210 and K237 are strictly conserved, and K268 and K269 are highly conserved. We suggest that one or more of these five conserved lysine residues are the targets for prodomanin ubiquitination by the SCF^Grr1^ ubiquitin-protein ligase complex and subsequent proteasomal degradation.

**Fig. S17. Interactions between Ssy5 and PP2A predicted by AlphaFold 3**. **A.** Close-up view of Fig. 6. The LxxVxE motif in Ssy5 is shown as green sticks. Interacting residues in Rts1 are shown as yellow sticks. Rts1 is shown in blue with the HEAT3-HEAT4 region highlighted in magenta. **B.** The interactions between LxxVxE and the HEAT3-HEAT4 region are very similar to those found between B56γ and BubR1 (Wang et al. 2016). Residues in the HEAT3-HEAT4 helices in Rts1 that interact with the Ssy5 LxxVxE site are highlighted in red. **C.** Close-up view of Fig. 10 showing that Ssy5 residues F575 and Q576 (cyan spheres), subject to gain-of-function mutations F575V and Q576P, are adjacent to Pph21 residues W269, F278, and V284 (yellow spheres). The Ssy5 mutations may diminish interactions at the Ssy5-Pph21 hydrophobic interface and thereby promote phosphorylation of Ssy5.

**Fig. S18. Binding of the Ssy5 phosphosites region to the catalytic cleft of Pph21. A.** Close-up of Fig. 6, showing positions of Ssy5 residues within the region from S70 to S92 (green), containing the four potential phosphosites, in the catalytic cleft of Pph21 (grey). Several residues conserved within the phosphoprotein protease (PPP) family of serine/threonine phosphatases are directly involved in the dephosphorylation process (Yigong Shi 2009, Oberoi et al 2016). The corresponding residues in Pph21 are highlighted: (i) residues D117, H119, D145, N177, H178, H227, and H301 (yellow sticks), which coordinate metal-ions Mn^2+^ and Fe^2+^ (not shown); (ii) residues R149 and R274 (magenta sticks), which also participate in the catalytic mechanism. **B.** Further close-up, showing that the potential phosphosites in Ssy5 (80-TYGT-83 and 83-TGAS-86, green sticks) are situated within the hydrophobic groove of Pph21 with Ssy5-T83 docked deep in the active site of Pph21 flanked by R149 and R274.

**Fig. S19. AlphaFold 3 model of Ssy5-PP2A complex showing pIDDT confidence estimates.** AlphaFold 3 analysis of full-length Ssy5 and structured parts of yeast PP2A (Pph21 residues 64 to 348, Tpd3 residues 31 to 635, and Rts1 residues 281 to 757). This yielded confidence parameters ipTM of 0.58 and pTM of 0.63. The per-atom confidence estimates (pIDDTs) are shown according to the confidence color key at the bottom of the figure.

**Fig. S20. Alignment of the N-terminal domains of 18 Ssy1s from *Saccharomycetaceae*.** Residues with similar physico-chemical properties are indicated with black bold characters in yellow boxes when conservation is above a threshold of 60% per column. Fully conserved residues are shown as white characters in red boxes. BoxA through BoxF are indicated with green boxes and TM1a with a blue box. The deletion Δ S167-N196 (red bar) did not interfere with SPS sensor function (Ring et al. 2019). Insertion of hemagglutinin epitopes at peptide bond 34/35 or at 68/69 yielded non-functional Ssy1s (red boxes), whereas insertion at 206/207 yielded a functional Ssy1 (green box) (Klasson et al. 1999).

**Figure S21. BoxE is located close to IL2-3 and the C-terminal latch. A.** Side view of an AlphaFold model of Ssy1 in an outward open conformation showing conserved boxes in the cytoplasmic N-terminal domain. Highlighted are BoxE (S209 to E227, magenta), IL2-3 (L339 to D356, blue), and TM12 plus the C-terminal (T782 to I852, red). BoxE is located close to IL2-3 and the C-terminal latch just beneath the cytoplasmic part of the transmembrane core. **B.** Intracellular close-up view showing BoxE spanning the cytoplasmic face of the transmembrane core close to IL2-3 and C-terminal sequences I810 to D826. Residues 1 to 207 are omitted for clarity.

**Fig. S22. Interaction between the LFA motif in Ptr3 and the prodomain of Ssy5. A.** Interactions between Ptr3 (N151-K678, split pea) and Ssy5 (N160-G699, salmon) (ipTM = 0.8, pTM = 0.79). Ptr3 residues T435, Q439, E522, and T525 are highlighted as cyan spheres. The LFA motif of Ptr3 (α-helix L165-D186) mediates interactions with α-helices M302-K314 and H353-L365 in the Ssy5 pro-domain. Ssy5 residues E512, F575, and Q576, subject to gain-of-function mutations, are highlighted (cyan spheres). The structured N-terminal part of Ptr3 (residues 1 to 110) is placed separately without interactions to Ssy5. **B.** Close-up of the AlphaFold 3 analysis showing the interaction between the Ptr3 LFA domain and the prodomain of Ssy5. Residues L165, L166, L168, F169, V172, and A173 (cyan sticks) of the LFA motif interact with a hydrophobic face of the prodomain formed by I308, L309, I319, I320, A326, I328, F346, and L348 (yellow sticks). **C.** Per-atom confidence estimates (pIDDTs) of the Ptr3-Ssy5 model. The confidence color key at the bottom of the figure is used.

**Fig. S23. Interaction of BoxD and BoxE in Ssy1 with blade 3 in Ptr3. A.** AlphaFold 3 analysis of Ptr3 residues 260-678 (green) and Ssy1 residues 1-283 (grey) (confidence metrics ipTM = 0.81 and pTM = 0.65). Ptr3 residues T435, Q439, E522 and T525 are shown as cyan sticks. Ssy1 residues L142, L147, Y151, T212, I215 and D216 are shown as yellow sticks. **B.** Close-up showing Ptr3 residues T435 and Q439 neighboring conserved Ssy1 residues L142, L147, and Y151 in BoxD as well as strictly conserved T212, I215 and D216 in BoxE. **C.** Close-up showing interactions between Ssy1 BoxD residues L142, L147, and Y151 and Ptr3 residues I428 and L441 in blade 3. **D.** Close-up showing interactions between Ssy1 BoxE and the Ptr3 loop between blade 3 and blade 4. Conserved BoxE residues T212, I215 and D216 (yellow sticks) face conserved Ptr3 residues P467, Y468, R469, and D470 (cyan sticks). **E.** Per-atom confidence estimates (pIDDTs) of the Ssy1-Ptr3 model. The confidence color key at the bottom of the figure is used. The Ssy1-Ptr3 interface is framed with an orange square **F.** Close-up. The positions of Ssy1 residues L142 and Y151 and of Ptr3 residues T435 and Q439 are indicated with red arrows.

**Fig. S24. BoxA in the Ssy1 N-terminal domain interacts with the catalytic domain of Ssy5. A.** AlphaFold 3 analysis of structured Ssy5 residues 160 to 699 (colored in salmon) and Ssy1 residues 1 to 283 (grey); the result yielded acceptable confidence parameters (ipTM = 0.79 and pTM = 0.67). The side chains of strictly conserved BoxA residues L10, F11, and P12 (yellow sticks) appear inserted into a hydrophobic pocket formed by Ssy5 residues V532, W533, I548, I689 and W691 (cyan sticks). **B.** Close-up. Indicated residues are shown as spheres **C.** AlphaFold 3 model of Ssy1-Ssy5 interaction showing per-atom confidence estimates (pIDDTs) according to the confidence color key at the bottom of the figure. **D.** Close-up showing L10, F11, and P12 motif interaction with Ssy5 catalytic domain.

**Fig. S25. Per-atom confidence estimates (pIDDT) of the Ssy1-Ptr3-Ssy5 complex. A.** AlphaFold 3 model of full-lengths of Ssy1, Ptr3, and Ssy5 (ipTM = 0.46 pTM = 0.53). The confidence estimates (pIDDTs) are illustrated according to the confidence color key at the bottom of the figure. **B.** Close-up showing the Ssy1-Ptr3 interface.

**Fig. S26. Interaction between Yck1 and BoxD in Ssy1. A.** AlphaFold 3 analysis of Ssy1 N-terminal residues (1 to 282) (grey), structured Yck1 residues (61 to 350) (orange) and an ATP molecule (magenta) yielded a confident model (ipTM = 0.81 and pTM = 0.67).

Ssy1 residues F110 and Y113 are shown as yellow sticks, while Yck1 F84 and F99 are shown as green sticks. **B.** Close-up showing potential hydrophobic and/or pi-pi interactions of Ssy1 residues F110 and Y113 with Yck1 residues F84 and F99, respectively. **C.** Per-atom confidence estimates (pIDDTs). **D.** Close-up of C.

**Fig. S27. Alignment of 50 Yck1 orthologs highlighting residues F84 and F99**

Alignment of 50 top hits from a BLAST sequence similarity search using the *S. cerevisiae* Yck1 sequence. UniProtKB accession numbers are shown to the left of the sequences. The upper Yck1 sequence is from *S. cerevisiae*. Strictly conserved residues F84 and F99 are highlighted with arrows.

**Fig. S28.** **Proposed Ptr3 phospho-sites are located in the Ssy1-Ptr3 interface. A.** AlphaFold 3 analysis of full lengths of Ssy1, Ptr3, and Ssy5 (iPTM = 0.46 pTM = 0.53). The various components are colored: Ssy1 N-terminal domain (pale cyan), Ssy1 TM bundle (grey), Ptr3 (split pea), and Ssy5 (salmon). Key residues are highlighted as cyan spheres, i.e. T435 and Q439, subject to gain-of-function *PTR3* mutations, E522 and T525, subject to loss-of-function *ptr3* mutations, and F575 and Q576, subject to gain-of-function *SSY5* mutations. The Ssy5-interacting LFA motif in Ptr3 and the Ssy1 C-terminal latch (L813-W830) are colored magenta and red, respectively. Proposed phosphorylation motifs in Ptr3 (S511 to S521) are colored blue. The intrinsically disordered region M1-E159 in Ssy5 is hidden for clarity. **B.** Close-up showing Ptr3 residues E522 and T525 and proposed Ptr3 phospho-sites in region S511 to S521 positioned close to the Ssy1 C-terminal latch. **C.** Similar close-up shown in spheres style, illustrating surface exposure. Proposed phosphorylated residues S514, T517, and S521 are shown as yellow spheres in the stretch of residues S511 to S521. T517 and S521 are buried within the sensor structure, thus inaccessible to phosphorylation. This accords with the shown conformation being outward-facing and non-signaling.

**Fig. S29. Residues T517 and S521 in the Ptr3 phosphosites loop are close to D817 and D819 in the Ssy1 latch.** Close-up of Ssy1-Ptr3 complex in Fig. S27A showing that the side chains of D817 and D819 in the Ssy1 latch are in close proximity of side chains T517 and S521 in the Ptr3 phosphosites loop. Phosphorylation of T517 and S521 most likely results in repulsion of D817 and D819 side chains, causing displacement of Ptr3. Ssy1 is colored grey with D817 and D819 highlighted as spheres. Ptr3 is colored green with T517 and S521 highlighted as yellow spheres and E522 and T525 as cyan spheres. Loss-of-function mutations E522K and T525A both abolish signaling. T525A prevents hyper-phosphorylation of Ptr3, and E522K may well also have this defect.

**Fig. S30. Displacement of Ptr3 from Ssy1 by phosphorylation. A.** AlphaFold 3 analysis (ipTM = 0.68 pTM = 0.6) of full-length Ssy1 (grey) and the structured part of Ptr3 (260-678) (split pea), in which Ptr3 residues S514, T517, and S521 (yellow spheres) are in a non-phosphorylated state. T435, Q439, E522, and T525 are indicated as cyan spheres. Ssy1 latch (813-830) (red). **B.** Similar analysis (ipTM = 0.72 pTM = 0.47) in which S514, T517, and S521 are phosphorylated (highlighted as yellow spheres). Major displacements of both Ptr3 and Ssy1 N-terminal are observed. **C.** Close-up of AlphaFold 3 analysis in panel A. **D.** Close-up of AlphaFold 3 analysis in panel B**.** Comparison of the non-phosphorylated and phosphorylated states of the Ssy1-Ptr3 complexes shows a large displacement of Ptr3 away from Ssy1 including a 180 degrees rotation of Ptr3. **E.** Per-atom confidence estimates (pIDDTs) of the Ssy1-Ptr3 complex in panel A colored according to the confidence key at the bottom of the figure. **F.** Close-up of the Ssy1-Ptr3 interface.

**Fig. S31. Dimerization of the N-terminal of Ptr3.** AlphaFold 3 model showing homodimerization of the N-terminal of Ptr3 (M1-T110) with chain A light orange and chain B pale green. Interacting side chains in chain A and chain B are shown as yellow and cyan sticks, respectively. Conserved residues C57, C59, C80 and C83 are shown as blue sticks. Ptr3 chains A and B are bound through interactions between their α-helices (H2-L25); i.e. side chains of I14, V17, L18, and I24 in chain A make hydrophobic interactions to I14, V17, L18, and I24 in chain B, respectively. Likewise, H-bonds may be formed between the two Q6 residues. Chain A and B also bind to each other by hydrophobic interactions between their L97 and L100 residues in the α-helices K95-S109. Finally, residues V17 and D24 in α-helix H2-L25 in chain A binds to residues F107 and Q103 in α-helix (K95-S109) in chain B through a hydrophobic and a H-bond interaction, respectively. *Vice versa*, V17 and D24 in chain B interacts with F107 and Q103 in Chain A.

**Literature cited**

Abdel-Sater F et al. 2004. Amino acid signaling in yeast: casein kinase I and the Ssy5 endoprotease are key determinants of endoproteolytic activation of the membrane-bound Stp1 transcription factor. Mol Cell Biol. 24(22):9771–9785. <https://doi.org/10.1128/mcb.24.22.9771-9785.2004>

Coleman JA, Green EM, Gouaux E. 2016. X-ray structures and mechanism of the human serotonin transporter. Nature. 532(7599):334–339. <https://doi.org/10.1038/nature17629>

Emenecker RJ, Griffith D, Holehouse AS. 2021. Metapredict: a fast, accurate, and easy-to-use predictor of consensus disorder and structure. Biophys J. 120(20):4312–4319. <https://doi.org/10.1016/j.bpj.2021.08.039>

Errasti-Murugarren E et al. 2019. L amino acid transporter structure and molecular bases for the asymmetry of substrate interaction. Nat Commun. 10(1):1807. <https://doi.org/10.1038/s41467-019-09837-z>

Fang Y et al. 2009. Structure of a prokaryotic virtual proton pump at 3.2 Å resolution. Nature. 460(7258):1040–1043. <https://doi.org/10.1038/nature08201>

Fort J et al. 2025. The conserved lysine residue in transmembrane helix 5 is pivotal for the cytoplasmic gating of the L-amino acid transporters. PNAS Nexus. 4(1):pgae584. <https://doi.org/10.1093/pnasnexus/pgae584>

Gao X et al. 2009. Structure and mechanism of an amino acid antiporter. Science. 324(5934):1565–1568. <https://doi.org/10.1126/science.1173654>

Gao X et al. 2010. Mechanism of substrate recognition and transport by an amino acid antiporter. Nature. 463(7282):828–832. <https://doi.org/10.1038/nature08741>

Gournas C, Prévost M, Krammer EM, André B. 2016. Function and regulation of fungal amino acid transporters: Insights from predicted structure. Adv Exp Med Biol. 892:69-106. <https://doi.org/10.1007/978-3-319-25304-6_4>

Ilgü H et al. 2021. High-resolution structure of the amino acid transporter AdiC reveals insights into the role of water molecules and networks in oligomerization and substrate binding. BMC Biol. 19(1):179. <https://doi.org/10.1186/s12915-021-01102-4>

Jungnickel KEJ, Parker JL, Newstead S. 2018. Structural basis for amino acid transport by the CAT family of SLC7 transporters. Nat Commun. 9(1):1–12. <https://doi.org/10.1038/s41467-018-03066-6>

Kanda N, Abe F. 2013. Structural and functional implications of the yeast high-affinity tryptophan permease Tat2. Biochemistry. 52(25):4296–4307. <https://doi.org/10.1021/bi4004638>

Lee Y et al. 2019. Cryo-EM structure of the human L-type amino acid transporter 1 in complex with glycoprotein CD98hc. Nat Struct Mol Biol. 26(6):510–517. <https://doi.org/10.1038/s41594-019-0237-7>

Ma D et al. 2012. Structure and mechanism of a glutamate-GABA antiporter. Nature. 483(7391):632–636. <https://doi.org/10.1038/nature10917>

Mok J et al. 2010. Deciphering protein kinase specificity through large-scale analysis of yeast phosphorylation site motifs. Sci Signal. 3(109):ra12. <https://doi.org/10.1126/scisignal.2000482>

Oda K et al. 2020. Consensus mutagenesis approach improves the thermal stability of system xc− transporter, xCT, and enables cryo-EM analyses. Protein Sci. 29(12):2398–2407. <https://doi.org/10.1002/pro.3966>

Omnus DJ, Ljungdahl PO. 2013. Rts1-protein phosphatase 2A antagonizes Ptr3-mediated activation of the signaling protease Ssy5 by casein kinase I. Mol Biol Cell. 24(9):1480–1492. <https://doi.org/10.1091/mbc.E13-01-0019>

Penmatsa A, Wang KH, Gouaux E. 2013. X-ray structure of dopamine transporter elucidates antidepressant mechanism. Nature. 503(7474):85–90. <https://doi.org/10.1038/nature12533>

Poulsen P, Leggio L Lo, Kielland-Brandt MC. 2006. Mapping of an internal protease cleavage site in the Ssy5p component of the amino acid sensor of *Saccharomyces cerevisiae* and functional characterization of the resulting pro- and protease domains by gain-of-function genetics. Eukaryot Cell. 5(3):601–608. <https://doi.org/10.1128/EC.5.3.601-608.2006>

Poulsen P, Wu B, Gaber RF, Kielland-Brandt MC. 2005. Constitutive signal transduction by mutant Ssy5p and Ptr3p components of the SPS amino acid sensor system in *Saccharomyces cerevisiae*. Eukaryot Cell. 4(6):1116–1124. <https://doi.org/10.1128/EC.4.6.1116-1124.2005>

Ring A, Martins A, Ljungdahl PO. 2019. Ssy1 functions at the plasma membrane as a receptor of extracellular amino acids independent of plasma membrane-endoplasmic reticulum junctions. Traffic. 20(10):775–784. <https://doi.org/10.1111/tra.12681>

Roth AF, Papanayotou I, Davis NG. 2011. The yeast kinase Yck2 has a tripartite palmitoylation signal. Mol Biol Cell. 22(15):2702–2715. <https://doi.org/10.1091/mbc.E11-02-0115>

Shaffer PL, Goehring A, Shankaranarayanan A, Gouaux E. 2009. Structure and mechanism of a Na+-independent amino acid transporter. Science. 325(5943):1010–1014. <https://doi.org/10.1126/science.1176088>

Usami Y et al. 2014. Functional mapping and implications of substrate specificity of the yeast high-affinity leucine permease Bap2. Biochim Biophys Acta Biomembr. 1838(7):1719–1729. <https://doi.org/10.1016/j.bbamem.2014.03.018>

Wang J et al. 2016. Crystal structure of a PP2A B56-BubR1 complex and its implications for PP2A substrate recruitment and localization. Protein Cell. 7(7):516–526. <https://doi.org/10.1007/s13238-016-0283-4>

Wu D et al. 2020. Structural basis for amino acid exchange by a human heteromeric amino acid transporter. Proc Natl Acad Sci. 117(35):21281–21287. <https://doi.org/10.1073/pnas.2008111117>

Wyant GA et al. 2017. mTORC1 activator SLC38A9 is required to efflux essential amino acids from lysosomes and use protein as a nutrient. Cell. 171(3):642-654.e12. <https://doi.org/10.1016/j.cell.2017.09.046>

Xu RM et al. 1995. Crystal structure of casein kinase-1, a phosphate-directed protein kinase. EMBO J. 14(5):1015–1023. <https://doi.org/10.1002/j.1460-2075.1995.tb07082.x>

Yamashita A et al. 2005. Crystal structure of a bacterial homologue of Na^+^/Cl^-^-dependent neurotransmitter transporters. Nature. 437(7056):215–223. <https://doi.org/10.1038/nature03978>

Yan R, Zhou J, et al. 2020a. Structural insight into the substrate recognition and transport mechanism of the human LAT2–4F2hc complex. Cell Discov. 6(1):20–23. <https://doi.org/10.1038/s41421-020-00207-4>

Yan R, Li Y, et al. 2020b. Cryo-EM structure of the human heteromeric amino acid transporter b^0,+^AT-rBAT. Sci Adv. 6(16):1–11. <https://doi.org/10.1126/sciadv.aay6379>

Yan R, Zhao X, Lei J, Zhou Q. 2019. Structure of the human LAT1–4F2hc heteromeric amino acid transporter complex. Nature. 568(7750):127–130. <https://doi.org/10.1038/s41586-019-1011-z>

**Table S1: Sequence identity and similarity among *S. cerevisiae* amino acid transporters in the YAT family**

|  | **Ssy1** | **Agp2** | **Tat1** | **Bap2** | **Bap3** | **Agp1** | **Gnp1** | **Tat2** | **Gap1** | **Hip1** | **Agp3** | **Put4** | **Dip5** | **Lyp1** | **Alp1** | **Can1** |
| --- | --- | --- | --- | --- | --- | --- | --- | --- | --- | --- | --- | --- | --- | --- | --- | --- |
| **Ssy1** |  | 35 | 33 | 34 | 33 | 34 | 33 | 37 | 38 | 37 | 36 | 37 | 42 | 37 | 40 | 38 |
| **Agp2** | 23 |  | 34 | 34 | 34 | 38 | 35 | 38 | 40 | 37 | 38 | 43 | 42 | 39 | 40 | 39 |
| **Tat1** | 20 | 21 |  | 58 | 58 | 60 | 60 | 52 | 54 | 48 | 38 | 39 | 39 | 40 | 43 | 41 |
| **Bap2** | 22 | 22 | 49 |  | 81 | 63 | 64 | 52 | 55 | 51 | 39 | 41 | 41 | 41 | 42 | 42 |
| **Bap3** | 22 | 21 | 48 | 73 |  | 63 | 63 | 52 | 54 | 50 | 38 | 38 | 39 | 41 | 42 | 40 |
| **Agp1** | 22 | 25 | 51 | 53 | 51 |  | 76 | 53 | 57 | 50 | 39 | 39 | 42 | 42 | 44 | 44 |
| **Gnp1** | 21 | 23 | 51 | 54 | 53 | 68 |  | 54 | 56 | 51 | 39 | 39 | 42 | 42 | 44 | 44 |
| **Tat2** | 24 | 25 | 38 | 39 | 38 | 39 | 40 |  | 56 | 54 | 38 | 42 | 43 | 44 | 45 | 42 |
| **Gap1** | 24 | 27 | 41 | 43 | 40 | 43 | 43 | 45 |  | 61 | 42 | 42 | 43 | 45 | 45 | 43 |
| **Hip1** | 25 | 24 | 36 | 41 | 39 | 39 | 39 | 42 | 49 |  | 39 | 42 | 41 | 42 | 42 | 43 |
| **Agp3** | 24 | 23 | 25 | 27 | 25 | 25 | 25 | 25 | 30 | 27 |  | 41 | 44 | 43 | 45 | 45 |
| **Put4** | 23 | 29 | 27 | 28. | 26 | 27 | 27 | 29 | 29 | 31 | 27 |  | 48 | 46 | 46 | 47 |
| **Dip5** | 27 | 27 | 27 | 29 | 27 | 29 | 29 | 30 | 30 | 30 | 30 | 35 |  | 48 | 50 | 51 |
| **Lyp1** | 23 | 26 | 28 | 30 | 29 | 28 | 29 | 30 | 33 | 30 | 29 | 32 | 35 |  | 71 | 72 |
| **Alp1** | 26 | 26 | 28 | 30 | 29 | 30 | 31 | 31 | 33 | 29 | 31 | 32 | 35 | 59 |  | 76 |
| **Can1** | 24 | 24 | 29 | 30 | 28 | 30 | 31 | 29 | 33 | 30 | 31 | 33 | 36 | 62 | 66 |  |

Sequence identity is shown in the left diagonal half, while sequence similarity is shown in the right diagonal half.

Percentages of sequence identity and similarity were calculated at the SIAS platform (<http://imed.med.ucm.es/Tools/sias.html>), developed by the Immunomedicine Group, Universidad Complutense Madrid.

**Table S2: Templates used for Phyre2-generated models of Ssy1.**

| PDB ID | Transporter | Organism | % Identity | Conformation | Function | Reference |
| --- | --- | --- | --- | --- | --- | --- |
| 6F2W | BasC | *Carnobacterium sp AT7* | 21 | Inward open, ligand bound | Alanine-Serine-Cysteine antiporter | (Errasti-Murugarren et al. 2019) |
| 3GIA | ApcT | *Methanocaldococcus jannaschii* | 20 | Inward facing, yet occluded, substrate-free | H+-coupled amino acid symporter | (Shaffer et al. 2009) |
| 6LID | b^0,+^AT | *Homo sapiens* | 18 | Inward open, substrate-free | Amino acid antiporter | (Yan et al. 2020b) |
| 7CCS | xCT | *Homo sapiens* | 18 | Inward open, substrate-free | Amino acid antiporter | (Oda et al. 2020) |
| 5OQT | GkApcT | *Geobacillus kaustophilus* | 19 | Inward-facing occluded, substrate-bound | H+-coupled amino acid symporter | (Jungnickel et al. 2018) |
| 3LRC | AdiC | *Escherichia coli*  *O157:H7* | 19 | Outward open, substrate-free | Arginine/agmatine antiporter | (Gao et al. 2009) |
| 4DJI | GadC | *Escherichia coli* | 16 | Inward-facing, closed by C-plug, substrate-free | Glutamate-GABA antiporter | (Ma et al. 2012) |
| 7CMH | LAT2 | *Homo sapiens* | 16 | Inward open, substrate-bound | Phe, Ile, Leu, Trp, Ala,  Ser and Thr transporter | (Yan et al. 2020a) |
| 6YUP | b^0,+^AT1 | *Homo sapiens* | 22 | Inward open, substrate-free | Neutral and basic amino acid antiporter | (Wu et al. 2020) |
| 6JMQ | LAT1-cd98hc | *Homo sapiens* | 17 | Inward open, substrate-free | Large neutral amino acid antiporter | (Lee et al. 2019) |
| 6IRT | LAT1-4f2hc | *Homo sapiens* | 15 | Inward open, substrate-bound | Large neutral amino acid antiporter | (Yan et al. 2019) |
| 5I6X | SERT | *Homo sapiens* | 14 | Outward open, inhibitor-bound | Serotonin transporter | (Coleman et al. 2016) |
| 4M48 | DAT | *Drosophila melanogaster* | 14 | Outward open, inhibitor-bound | Dopamine transporter | (Penmatsa et al. 2013) |
| 2A65 | LeuT | *Aquifex aeolicus* | 13 | Occluded, substrate bound | Na^+^-coupled Leu transporter | (Yamashita et al. 2005) |

The three-dimensional structure templates used for Ssy1 modeling were identified by analysis of the S. cerevisiae Ssy1 amino acid sequence on the Phyre2 server (Kelley al, 2015). They are ranked according to a raw alignment score calculated by the server with the best hit at the top.

**Table S3: Hydrogen-bond interactions between substrates and APC family amino acid transporters**

| **Transporter structure**  (PDB codes) | **Transporter residue**  **engaged in H-bonding**  **with the α-carboxy**  **group of the substrate** | **Corresponding**  **Ssy1 residue** | **Transporter residue**  **engaged in H-bonding**  **with the α-amino**  **group of the substrate** | **Corresponding**  **Ssy1 residue** |
| --- | --- | --- | --- | --- |
| AdiC + arginine (3OB6) and (3L1L)  (antiporter) | S26 | V297 | I23 | C294 |
|  | G27 | G298 | W202 | F512 |
|  |  |  | I205 | S515 |
| GkApcT + alanine (5OQT) f)  (H+symporter) | T43 | V297 | F231 | F512 |
|  | G44 | G298 | A232 | A513 |
|  |  |  | I234 | S515 |
| hLAT1 + BCH ^e)^ (6IRT)  (antiporter) | G65 | S296 | F252 ^a)^ | F512 |
|  | S66 | V297 | G255 | S515 |
| hLAT2 + leucine (7CMI)  (antiporter) | G55 | S296 | I53 | C294 |
|  | G57 | G298 | F243 ^a)^ | F512 |
|  |  |  | G246 | S515 |
| hLAT2 + tryptophan (7CMH)  (antiporter) | Position 1 ^c)^ G55 | S296 | Position 1 ^c)^ F243 | F512 |
|  | Position 1 ^c)^ S56 | V297 |  |  |
|  | Position 2 N395 | T713 ^d)^ | Position 2 ^c)^ N134 | L374 ^d)^ |
| BasC + 2-AIB (6F2W)  (antiporter) | A20 | V297 | V17 | C294 |
|  | G21 | G298 | F199 | F512 |
|  |  |  | A200 | A513 |
|  |  |  | D202 | S515 |
| AgcS + alanine (6CSE)  (Na+symporter) | G77 | S296 | A74 | A293 |
|  | T78 | V297 | T75 | C294 |
|  | Q170 | S380 | S274 | S510 |
|  |  |  | E276 ^b)^ | F512 |
| b^0,+^AT + arginine (6LI9)  (antiporter) | G45 | S296 | I43 | C294 |
|  | G47 | G298 | D233 | S515 |
| SLC38A9+ arginine  (6C08) | Y204 | G363 | T121 | V297 |
|  |  |  | S122 | G298 |

^a)^ Hydrophobic interaction. ^b)^ Protonation state may affect substrate binding. ^c)^ Two binding positions. ^d)^ H-bond to side chain not possible in Ssy1 since the side chain is different from LAT2. ^e)^ 2-amino-2-norbornanecarboxylic acid. ^f)^In GkApcT-M321S (6F34), arginine is positioned with the α-carboxy and amino termini making identical interactions to those observed with L-Ala, with the guanidinium group of the side chain extending down towards the cytosol (Jungnickel et al 2018).
